# Metal-Free Synthesis of Carbamoylated Chroman-4-Ones via Cascade Radical Annulation of 2-(Allyloxy)arylaldehydes with Oxamic Acids

**DOI:** 10.3390/molecules27207049

**Published:** 2022-10-19

**Authors:** Long-Yong Xie, Sha Peng, Li-Hua Yang, Xiao-Wen Liu

**Affiliations:** Key Laboratory of Comprehensive Utilization of Advantage Plants Resources of Southern Hunan, College of Chemistry and Bioengineering, Hunan University of Science and Engineering, Yongzhou 425100, China

**Keywords:** carbamoylation, chroman-4-ones, 2-(allyloxy)arylaldehydes, cascade annulation, metal-free

## Abstract

An efficient and straightforward approach for the synthesis of carbamoylated chroman-4-ones has been well-developed. The reaction is triggered through the generation of carbamoyl radicals from oxamic acids under metal-free conditions, which subsequently undergoes decarboxylative radical cascade cyclization on 2-(allyloxy)arylaldehydes to afford various amide-containing chroman-4-one scaffolds with high functional group tolerance and a broad substrate scope.

## 1. Introduction

**Scheme 1:**
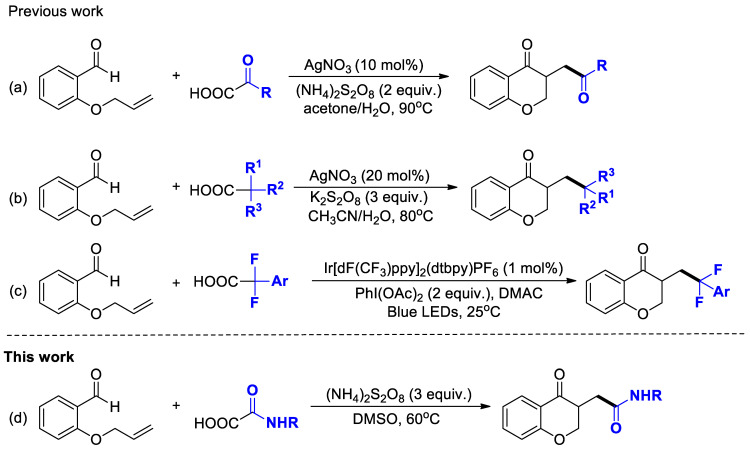
Decarboxylative cyclization of 2-(allyloxy)arylaldehydes using carboxylic acids as radical precursors (**a**) Silver-catalyzed cascade decarboxylative cyclization reaction between 2-(allyloxy)arylaldehydes and *α*-oxocarboxylic acids; (**b**) Silver nitrate-catalyzed cascade decarboxylation-cyclization of aliphatic acids with 2-(allyloxy)arylaldehydes; (**c**) Iridium-catalyzed cascade decarboxylative radical annulation of 2-(allyloxy)arylaldehydes with difluoroacetic acids; (**d**) (NH_4_)_2_S_2_O_8_-mediated decarboxylative cyclization of 2-(allyloxy)arylaldehydes with oxamic acids.

Chroman-4-one is one of the most important structural motifs that occur in pharmaceuticals and natural products that exhibit various biological activities, such as antibacterial, antioxidant, SIRT2 inhibitors, anti-HIV and estrogenic properties [1,2,3,4]. In the past few years, great efforts have been devoted to access such compounds [5,6]. For example, the base-promoted condensation of 2-hydroxyacetophenones with aldehydes, the *N*-heterocyclic carbine (NHC)-catalyzed intramolecular Stetter reaction, and the 1,4-conjugate addition to chromones with various nucleophiles are among the representative methods [7,8,9,10,11,12,13]. However, these methods often encounter some shortcomings, such as harsh reaction conditions, multi-step production and limited substrate scopes. Recently, the cascade radical annulation of 2-(allyloxy)arylaldehydes triggered by diverse radicals, including alkyl [14,15,16,17,18,19], acyl [20,21], trifluoromethyl [22,23], phosphoryl [22,23,24,25], and sulfonyl radicals [26,27,28] has been emerging as an atom- and step-economical approach for the construction of various functionalized chroman-4-ones. Among these methodologies, decarboxylative radical annulation of 2-(allyloxy)arylaldehydes using different types of carboxylic acids as radical precursors has made great achievements. In 2017, Wu et al. first reported a silver-catalyzed cascade decarboxylative cyclization reaction between 2-(allyloxy)arylaldehydes and *α*-oxocarboxylic acids directly accessing carbonyl-incorporated chroman-4-ones (Scheme 1a) [29]. Later, Yu and colleagues developed a silver nitrate-catalyzed cascade decarboxylation-cyclization process of aliphatic acids with 2-(allyloxy)arylaldehydes toward alkylated chroman-4-ones (Scheme 1b) [30]. Recently, Zhu and colleagues presented a method for difluoroalkylated chroman-4-ones via iridium-catalyzed and visible-light-induced cascade decarboxylative radical annulation of 2-(allyloxy)arylaldehydes with difluoroacetic acids as the difluoroalkylation reagents (Scheme 1c) [31]. Despite these progresses, and considering that most of the carboxylic acids are air-stable, readily available, and low cost, developing a more practical and efficient radical decarboxylative-cyclization reaction of 2-(allyloxy)arylaldehydes is desirable and of great significance.

On the other hand, amides are extremely important because of the ubiquitous existence of amide motifs in many pharmaceuticals, agrochemicals, natural products, and functional materials [32,33,34,35]. During the past few decades, various efficient synthetic methods have been widely explored for the construction of amide units [34,36,37,38]. Traditionally, synthesis of amides relies on the condensation reactions of carboxylic acids, acyl chlorides or anhydrides with various amines [39,40]. These approaches need the pre-installation of a carboxyl group in the substrate. Doubtlessly, the direct introduction of a carbamoyl group to organic molecules represents a more efficient strategy. In this study, we speculate whether carbamoyl radicals could participate in the cascade annulation process with o-(allyloxy)arylaldehydes to construct amide-containing chroman-4-ones. To the best of our knowledge, the carbamoyl-radical-triggered cascade radical cyclization of 2-(allyloxy)arylaldehydes toward amide-functionalized chroman-4-ones has never been reported.

For this reason, and because of the demand for practical and environmentally friendly approaches to various functionalized chroman-4-ones, we herein disclose a (NH_4_)_2_S_2_O_8_-mediated protocol for selective intermolecular radical decarboxylative cyclization of 2-(allyloxy)arylaldehydes with oxamic acids to access diverse carbamoylated chroman-4-one derivatives under metal-free conditions (Scheme 1d).

## 2. Results and Discussion

Initially, 2-(allyloxy)benzaldehyde (**1a**) and 2-oxo-2-(phenylamino)acetic acid (**2a**) were used as the model substrates to optimize the reaction conditions (Table 1). When the reaction was preceded using (NH_4_)_2_S_2_O_8_ as the oxidant and DMSO as the solvent, at 70 °C under a N_2_ atmosphere for 12 h, the desired product **3aa** was obtained at a 78% isolated yield (Entry 1). Some other common solvents including CH_3_CN, DMF, DCE, THF, acetone, and H_2_O were also investigated. To our surprise, only DMSO was effective for the current reaction and the desired product **3aa** was not detected with other examined solvents (Entries 2–7). Furthermore, various oxidants for this transformation were tested. (NH_4_)_2_S_2_O_8_ was found to be the best oxidant, whereas other oxidants, such as Na_2_S_2_O_8_, K_2_S_2_O_8_, TBHP, PhI(OAc)_2_, and Selectfluor did not generate the target product (Entry 1 vs. Entries 8–12). Specially, in contrast to (NH_4_)_2_S_2_O_8__,_ Na_2_S_2_O_8_ and K_2_S_2_O_8_ showed no activities in the current reaction; the solubility of these oxidants in DMSO may explain why (NH_4_)_2_S_2_O_8_ is efficient for the current reaction compared to Na_2_S_2_O_8_ and K_2_S_2_O_8_. We found that (NH_4_)_2_S_2_O_8_ was completely soluble in DMSO in our reaction system, while Na_2_S_2_O_8_ and K_2_S_2_O_8_ were only slightly soluble in DMSO. By decreasing the reaction temperature from 70 °C to 60 °C, a slight improved yield of **3aa** was achieved (Entry 13 vs. Entry 1), while further reducing the temperature to 50 °C or increasing to 80 °C resulted in lower yields (Entries 14–15 vs. Entry 13). In addition, reducing the amount of (NH_4_)_2_S_2_O_8_ or **2a** was not beneficial for the reaction and produced a lower yield (Entries 16–17). When the reaction was carried out in an open air, a 68% yield for **3aa** was obtained, indicating that a N_2_ atmosphere is crucial for improving the yield (Entry 18 vs. Entry 13). Additionally, in the absence of the oxidant, no reaction occurred, suggesting that an oxidant is essential for the current reaction (Entry 19).

**Scheme 2:**
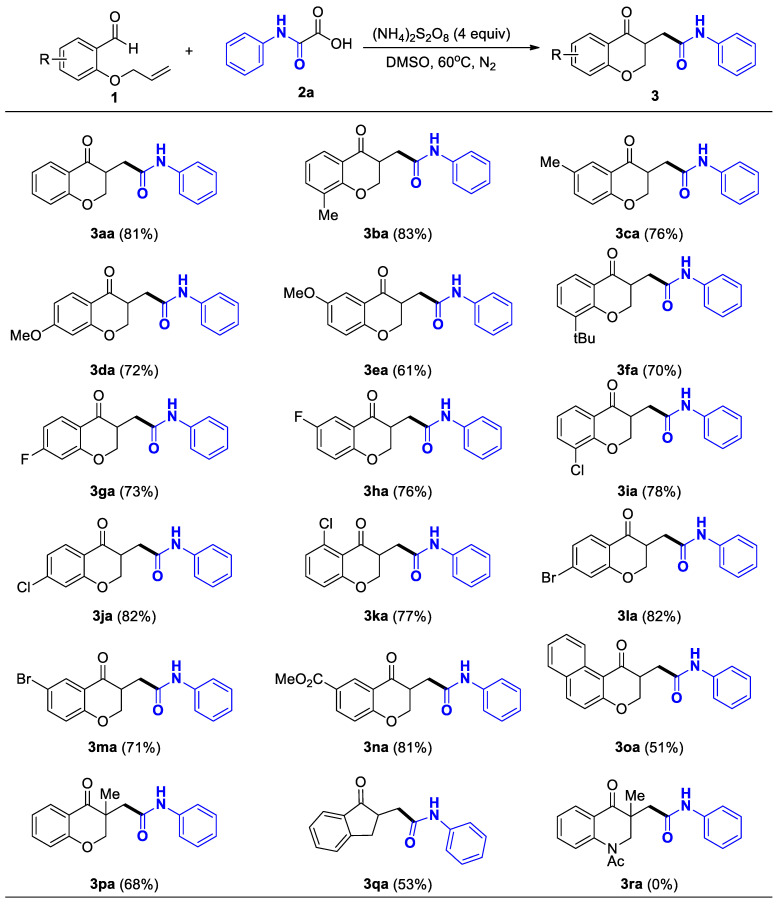
Preparation of **3aa**–**3ra**. Conditions: **1** (0.3 mmol), **2a** (0.9 mmol), (NH_4_)_2_S_2_O_8_ (1.2 mmol), DMSO (2 mL), 60 °C, under N_2_ atmosphere for 12 h. Isolated yields are given.

With the optimal reaction conditions established (Table 1, entry 13), we first explored the generality of the reaction by employing various 2-(allyloxy)arylaldehydes with 2-oxo-2-((phenylamino)acetic acid (**2a**). As depicted in Scheme 2, 2-(allyloxy)benzaldehydes bearing either electron-donating groups (Me, t-Bu and OMe) or electron-withdrawing groups (F, Cl, Br, CO_2_Me) all proceeded smoothly, affording the desired products at moderate to good yields (**3aa**–**3na**) (Supplementary Materials). Furthermore, the naphthalene-derived substrate could also undergo transformation to obtain **3oa** at a moderate yield. To our delight, substrate **1p** bearing a methyl group close to C=C bond and 2-allylbenzaldehyde **1q** also reacted well, providing product **3pa** and **3qa** at 68% and 53% yields, respectively. However, *N*-allyl-*N*-(2-formylphenyl)acetamide failed to generate the expected product (**3ra**).

**Scheme 3:**
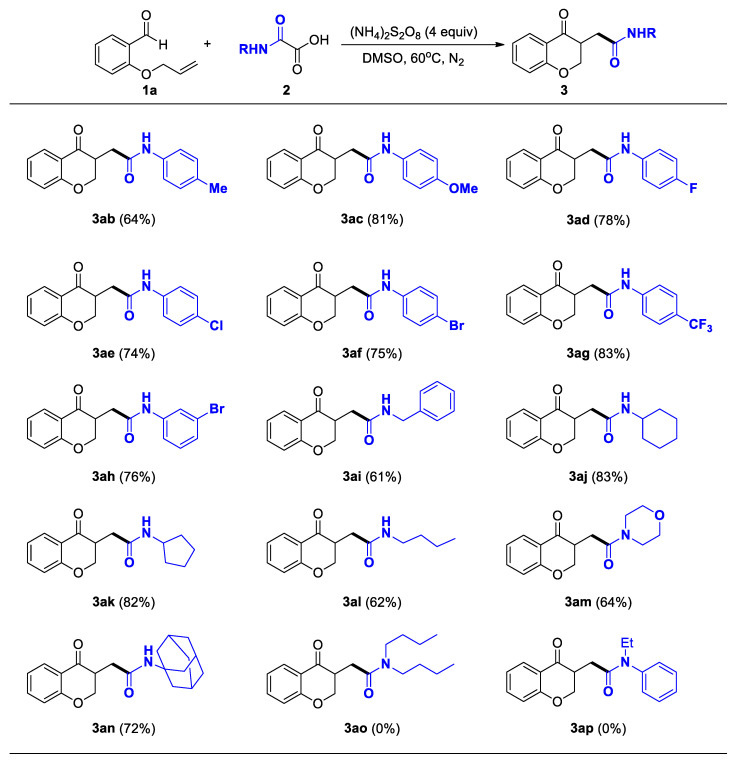
Preparation of **3ab**–**3ap**. Conditions: **1a** (0.3 mmol), **2** (0.9 mmol), (NH_4_)_2_S_2_O_8_ (1.2 mmol), DMSO (2 mL), 60 °C, under N_2_ atmosphere for 12 h. Isolated yields are given.

We next investigated the scope of this decarboxylative radical cyclization by varying oxamic acids with o-(allyloxy)aryl-aldehydes (**1a**), as shown in Scheme 3. *N*-aryl oxamic acids with electron-donating and electron-withdrawing groups all provided the desired products at 63–83% yields. Some important functional groups, such as alkyl (**3ab**), alkoxyl (**3ac**), halide (**3ad**–**3af** and **3ah**), and CF_3_ (**3ag**) groups at different benzene rings positions were well-compatible. Furthermore, *N*-alkyl oxamic acids were also suitable substrates. Various alkyl groups, including benzyl (**3ai**), cyclohexyl (**3aj**), cyclopentyl (**3ak**), butyl (**3al**), and adamantly (**3an**), smoothly proceeded to provide the desired products at good yields. However, using **2o** and **2p** as substrates, no desired products (**3ao** and **3ap**) were detected and most of substrate **1a** was recycled. GC-Ms showed that **2o** and **2p** were almost converted to the corresponding *N*,*N*-dibutylformamide and *N*-ethyl-*N*-phenylformamide via the release of CO_2_. In addition, we also tried using 2-oxo-2-phenylacetic, pivalic, and 2,2-difluoro-2-phenylacetic acids as substrates instead of oxamic acid **2,** but no desired decarboxylative cyclization products were obtained.

**Scheme 4:**
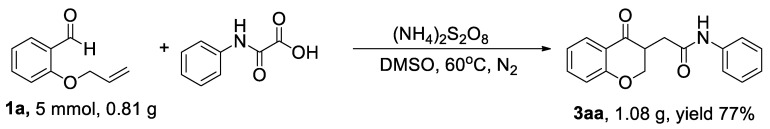
Gram-scale synthesis of **3aa**.

To demonstrate the scalability of this protocol, a gram-scale reaction was conducted by using substrate **1a** (5 mmol, 0.81 g) with **2a** under the standard reaction conditions (Scheme 4). As anticipated, the desired product **3aa** was obtained at a 77% isolated yield, which suggests that the present reaction is a practical method for the synthesis of various carbamoylated chroman-4-ones.

**Scheme 5:**
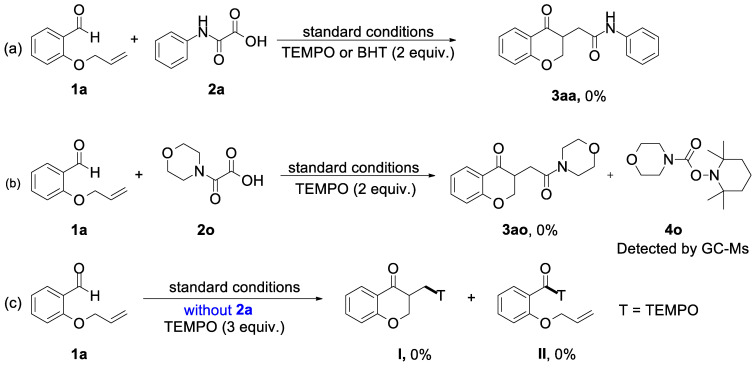
Control experiments (**a**) Radical inhibition experiment using **TEMPO** or **BHT** as radical inhibitor; (**b**) Radical capture experiment between **1a** and **2o** using TEMPO as a radical scavenger; (**c**) Reaction between **1a** and **TEMPO** under standard conditions.

To better understand the cascade annulation process, several control experiments were carried out (Scheme 5). When the reaction was performed in the presence of 2 equiv. of radical scavengers (TEMPO or BHT) under the standard reaction conditions, the desired product **3aa** was not observed (Scheme 5a), suggesting that a free-radical pathway might be involved in the current transformation. In addition, in conducting the reaction between **1a** and **2o** in the presence of 2 equiv. of TEMPO, TEMPO-adduct **4o** was detected by GC-MS analysis (Scheme 5b), which implied that carbamoyl radicals generated during the reaction. Furthermore, radical adducts **I** and **II** were not detected upon adding 3 equiv. of TEMPO in the absence of **2a** under the standard conditions (Scheme 5c), indicating that a radical–radical coupling process could be ruled out.

**Scheme 6:**
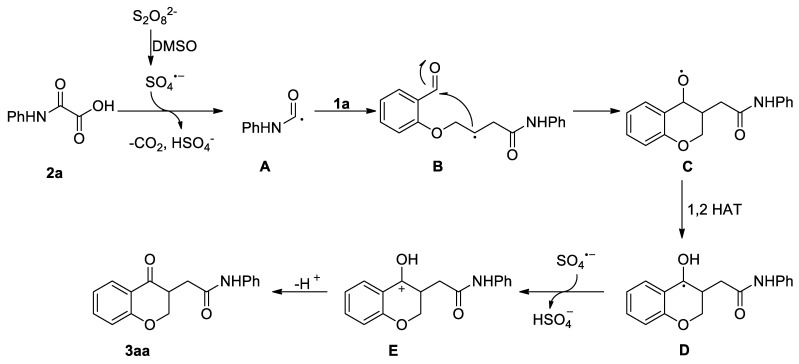
Possible mechanism.

On the basis of the above control experiment and the recent literature [41,42,43,44,45,46,47,48,49,50], a plausible reaction pathway for this carbamoylation reaction is proposed. As shown in Scheme 6, initially, the radical anion SO_4_^−•^ generates via the decomposition of (NH_4_)_2_S_2_O_8_ in DMSO. The resulting sulfate radical anion SO_4_^−•^ performs hydrogen atom transfer (HAT) with 2-oxo-2-(phenylamino)acetic acid (**2a**) to form the carbamoyl radical A alongside emission of CO_2_. The radical A attacks the carbon–carbon double bond of **1a** to form radical intermediate **B.** Then, the radical intermediate **B** cyclizes to afford an oxygen radical **C**, which undergoes a 1,2-hydrogen atom transfer (HAT) process to deliver the benzyl radical **D.** Finally, the further oxidation of intermediate **D** results in the corresponding carbocation **E** with the remaining sulfate radical anion SO_4_^−•^**,** followed by deprotonation to generate the target product **3aa**.

## 3. Materials and Methods

### 3.1. General Information

Unless otherwise specified, all reagents and solvents were obtained from commercial suppliers and used without further purification. All reagents were weighed and handled in air at room temperature. ^1^H-NMR spectra were recorded at 400 MHz and ^13^C-NMR spectra were recorded at 100 MHz by using a German Bruker Avance 400 spectrometer. Chemical shifts were calibrated using residual undeuterated solvent as an internal reference (^1^H-NMR: CDCl_3_ 7.26 ppm, ^13^C-NMR: CDCl_3_ 77.0 ppm). The following abbreviations were used to describe peak splitting patterns when appropriate: s = singlet, d = doublet, t = triplet, q = quartet, m = multiplet. Mass spectra were performed on a spectrometer operating on ESI-TOF.

### 3.2. General Procedure for the Preparation of Carbamoylated Chroman-4-Ones

To a solution of 2-(allyloxy)arylaldehyde **1** (0.3 mmol) and oxamic acid **2** (0.9 mmol) in DMSO (2 mL), (NH_4_)_2_S_2_O_8_ (1.2 mmol) was added. The reaction mixture was stirred at 60 °C under N_2_ atmosphere conditions. The progress of the reaction was monitored by TLC. The reaction typically finished within 12 h. After completion, water (10 mL) was added and the mixture was extracted with EtOAc (10 mL × 3); the solvent was then removed under vacuum. The residue was purified by flash column chromatography using a mixture of petroleum ether and ethyl acetate as eluent to generate the desired products **3**.

### 3.3. Gram-Scale Synthesis of ***3aa***

To a solution of 2-(allyloxy)benzaldehyde **1a** (0.81 g, 5 mmol) and 2-oxo-2-(phenylamino)acetic acid **2a** (2.48 g, 15 mmol) in DMSO (30 mL), (NH_4_)_2_S_2_O_8_ (4.56 g, 20 mmol) was added. The reaction mixture was stirred at 60 °C under N_2_ atmosphere conditions for 12 h. After completion, water (50 mL) was added and the mixture was extracted with EtOAc (50 mL × 3); the solvent was then removed under vacuum. The residue was purified by flash column chromatography using a mixture of petroleum ether and ethyl acetate as eluent to produce 1.08 g of **3aa**, yielding 77%.

### 3.4. Characterization Data of Products ***3aa**–**3qa*** and ***3ab**–**3an***

2-(4-oxochroman-3-yl)-N-phenylacetamide (**3aa**):

^1^H-NMR (400 MHz, Chloroform-*d*) δ 8.13 (s, 1 H), 7.89 (d, *J* = 7.8 Hz, 1 H), 7.60–7.44 (m, 3 H), 7.31 (t, *J* = 7.5 Hz, 2 H), 7.10 (t, *J* = 7.3 Hz, 1 H), 7.06–6.93 (m, 2 H), 4.68 (dd, *J* = 11.2, 5.3 Hz, 1 H), 4.31 (t, *J* = 11.9 Hz, 1 H), 3.46–3.37 (m, 1 H), 2.92 (dd, *J* = 15.0, 5.9 Hz, 1 H), 2.49 (dd, *J* = 15.0, 5.9 Hz, 1 H); ^13^C-NMR (100 MHz, Chloroform-*d*) δ 194.3, 168.6, 161.9, 137.7, 136.4, 129.0, 127.3, 124.3, 121.5, 120.3, 119.8, 117.9, 70.5, 42.9, 33.7; HR-MS (ESI): *m/z* [M + H]^+^ calcd for C_17_H_16_NO_3_: 282.1125; found: 282.1128.

2-(8-methyl-4-oxochroman-3-yl)-*N*-phenylacetamide (**3ba**):

^1^H-NMR (400 MHz, Chloroform-*d*) δ 8.22 (s, 1 H), 7.74 (d, *J* = 7.8 Hz, 1 H), 7.52 (d, *J* = 7.9 Hz, 2 H), 7.38–7.27 (m, 3 H), 7.09 (t, *J* = 7.3 Hz, 1 H), 6.92 (t, *J* = 7.6 Hz, 1 H), 4.71 (dd, *J* = 11.2, 5.3 Hz, 1 H), 4.28 (t, *J* = 11.9 Hz, 1 H), 3.42–3.35 (m, 1 H), 2.91 (dd, *J* = 15.0, 6.0 Hz, 1 H), 2.48 (dd, *J* = 15.0, 5.9 Hz, 1 H), 2.23 (s, 3 H); ^13^C-NMR (100 MHz, Chloroform-*d*) δ 194.7, 168.7, 160.2, 137.8, 137.2, 128.9, 127.3, 124.9, 124.3, 120.9, 119.9, 119.8, 70.3, 42.8, 33.8, 15.5; HR-MS (ESI): *m/z* [M + H]^+^ calcd for C_18_H_18_NO_3_: 296.1281; found: 296.1285.

2-(6-methyl-4-oxochroman-3-yl)-*N*-phenylacetamide (**3ca**):

^1^H-NMR (400 MHz, Chloroform-*d*) δ 8.13 (s, 1 H), 7.68 (s, 1 H), 7.52 (d, *J* = 7.9 Hz, 2 H), 7.31 (t, *J* = 7.1 Hz, 3 H), 7.10 (t, *J* = 7.3 Hz, 1 H), 6.88 (d, *J* = 8.4 Hz, 1 H), 4.65 (dd, *J* = 11.2, 5.3 Hz, 1 H), 4.27 (t, *J* = 11.8 Hz, 1 H), 3.43–3.34 (m, 1 H), 2.90 (dd, *J* = 15.0, 6.0 Hz, 1 H), 2.49 (dd, *J* = 15.0, 5.9 Hz, 1 H), 2.30 (s, 3 H); ^13^C-NMR (100 MHz, Chloroform-*d*) δ 194.6, 168.6, 160.0, 137.8, 137.5, 131.0, 129.0, 126.9, 124.3, 119.8, 119.9, 117.7, 70.5, 43.0, 33.9, 20.4; HR-MS (ESI): *m/z* [M + H]^+^ calcd for C_18_H_18_NO_3_: 296.1281; found: 296.1287.

2-(7-methoxy-4-oxochroman-3-yl)-*N*-phenylacetamide (**3da**):

^1^H-NMR (400 MHz, Chloroform-*d*) δ 8.36 (s, 1 H), 7.82 (d, *J* = 8.8 Hz, 1 H), 7.53 (d, *J* = 7.9 Hz, 2 H), 7.30 (t, *J* = 7.7 Hz, 2 H), 7.09 (t, *J* = 7.3 Hz, 1 H), 6.59 (d, *J* = 8.8 Hz, 1 H), 6.41 (s, 1 H), 4.65 (dd, *J* = 11.1, 5.3 Hz, 1 H), 4.28 (t, *J* = 11.8 Hz, 1 H), 3.83 (s, 3 H), 3.38–3.29 (m, 1 H), 2.91 (dd, *J* = 15.0, 6.1 Hz, 1 H), 2.46 (dd, *J* = 15.0, 5.8 Hz, 1 H); ^13^C-NMR (100 MHz, Chloroform-*d*) δ 192.9, 168.8, 166.4, 164.0, 137.8, 129.1, 128.9, 124.2, 119.8, 114.1, 110.4, 100.6, 70.8, 55.7, 42.5, 34.0; HR-MS (ESI): *m/z* [M + H]^+^ calcd for C_18_H_18_NO_4_: 312.1230; found: 312.1233.

2-(6-methoxy-4-oxochroman-3-yl)-*N*-phenylacetamide (**3ea**):

^1^H-NMR (400 MHz, Chloroform-*d*) δ 8.04 (s, 1 H), 7.51 (t, *J* = 9.9 Hz, 2 H), 7.32 (t, *J* = 7.5 Hz, 3 H), 7.11 (t, *J* = 7.3 Hz, 2 H), 6.92 (d, *J* = 9.0 Hz, 1 H), 4.64 (dd, *J* = 11.2, 5.2 Hz, 1 H), 4.29 (t, *J* = 11.8 Hz, 1 H), 3.80 (s, 3 H), 3.42–3.35 (m, 1 H), 2.90 (dd, *J* = 15.0, 5.9 Hz, 1 H), 2.51 (dd, *J* = 15.0, 5.9 Hz, 1 H); ^13^C-NMR (100 MHz, Chloroform-*d*) δ 194.3, 168.5, 156.7, 154.1, 137.7, 129.0, 125.7, 124.4, 120.1, 119.8, 119.2, 107.6, 70.6, 55.8, 43.0, 33.9; HR-MS (ESI): *m/z* [M + H]^+^ calcd for C_18_H_18_NO_4_: 312.1230; found: 312.1232.

2-(8-(tert-butyl)-4-oxochroman-3-yl)-*N*-phenylacetamide (**3fa**):

^1^H-NMR (400 MHz, Chloroform-*d*) δ 8.25 (s, 1 H), 7.81 (d, *J* = 7.8 Hz, 1 H), 7.51 (dd, *J* = 13.8, 7.8 Hz, 3 H), 7.31 (t, *J* = 7.6 Hz, 2 H), 7.09 (t, *J* = 7.3 Hz, 1 H), 6.96 (t, *J* = 7.7 Hz, 1 H), 4.74 (dd, *J* = 11.1, 5.3 Hz, 1 H), 4.28 (t, *J* = 11.9 Hz, 1 H), 3.45–3.36 (m, 1 H), 2.93 (dd, *J* = 15.0, 5.9 Hz, 1 H), 2.50 (dd, *J* = 15.0, 5.9 Hz, 1 H), 1.38 (s, 9 H); ^13^C-NMR (100 MHz, Chloroform-*d*) δ 195.1, 168.8, 161.1, 139.1, 137.8, 133.4, 128.9, 125.4, 124.3, 121.1, 121.0, 119.8, 70.0, 42.8, 34.9, 33.9, 29.5; HR-MS (ESI): *m/z* [M + H]^+^ calcd for C_21_H_24_NO_3_: 338.1751; found: 338.1747.

2-(7-fluoro-4-oxochroman-3-yl)-*N*-phenylacetamide (**3ga**):

^1^H-NMR (400 MHz, Chloroform-*d*) δ 8.03 (s, 1 H), 7.96–7.84 (m, 1 H), 7.51 (d, *J* = 7.9 Hz, 2 H), 7.31 (t, *J* = 7.7 Hz, 2 H), 7.10 (t, *J* = 7.3 Hz, 1 H), 6.75 (t, *J* = 8.3 Hz, 1 H), 6.67 (d, *J* = 9.7 Hz, 1 H), 4.70 (dd, *J* = 11.2, 5.4 Hz, 1 H), 4.33 (t, *J* = 11.9 Hz, 1 H), 3.47–3.35 (m, 1 H), 2.91 (dd, *J* = 15.1, 5.8 Hz, 1 H), 2.49 (dd, *J* = 15.1, 6.1 Hz, 1 H); ^13^C-NMR (100 MHz, Chloroform-*d*) δ 192.7, 168.4, 167.6 (d, *J*_C-F_ = 256.0 Hz), 163.6 (d, *J*_C-F_ = 14.0 Hz), 137.7, 130.0 (d, *J*_C-F_ = 12.0 Hz), 129.0, 124.4, 119.8, 117.3 (d, *J*_C-F_ = 23.0 Hz), 110.1(d, *J*_C-F_ = 23.0 Hz), 104.7 (d, *J*_C-F_ = 24.0 Hz), 70.9, 42.6, 33.5; ^19^F-NMR (376 MHz, Chloroform-*d*) δ −99.6; HR-MS (ESI): *m/z* [M + H]^+^ calcd for C_17_H_15_FNO_3_: 300.1030; found: 300.1036.

2-(6-fluoro-4-oxochroman-3-yl)-*N*-phenylacetamide (**3ha**):

^1^H-NMR (400 MHz, Chloroform-*d*) δ 8.04 (s, 1 H), 7.51 (d, *J* = 7.0 Hz, 3 H), 7.30 (t, *J* = 7.4 Hz, 2 H), 7.20 (d, *J* = 7.8 Hz, 1 H), 7.10 (t, *J* = 7.2 Hz, 1 H), 6.96 (dd, *J* = 9.0, 3.9 Hz, 1 H), 4.67 (dd, *J* = 11.2, 5.3 Hz, 1 H), 4.31 (t, *J* = 11.9 Hz, 1 H), 3.45–3.36 (m, 1 H), 2.91 (dd, *J* = 15.2, 5.5 Hz, 1 H), 2.51 (dd, *J* = 15.2, 6.3 Hz, 1 H); ^13^C-NMR (100 MHz, Chloroform-*d*) δ 193.4, 168.4, 158.2, 157.2 (d, *J*_C-F_ = 241.0 Hz), 137.6, 129.0, 124.4, 123.9 (d, *J*_C-F_ = 25.0 Hz), 119.8, 119.6 (d, *J*_C-F_ = 7.0 Hz), 114.3, 112.2 (d, *J*_C-F_ = 23.0 Hz), 70.6, 42.8, 33.5; ^19^F-NMR (376 MHz, Chloroform-*d*) δ −121.2; HR-MS (ESI): *m/z* [M + H]^+^ calcd for C_17_H_15_FNO_3_: 300.1030; found: 300.1034.

2-(8-chloro-4-oxochroman-3-yl)-*N*-phenylacetamide (**3ia**):

^1^H-NMR (400 MHz, Chloroform-*d*) δ 7.97 (s, 1 H), 7.80 (d, *J* = 7.8 Hz, 1 H), 7.57 (d, *J* = 7.7 Hz, 1 H), 7.50 (d, *J* = 7.8 Hz, 2 H), 7.30 (t, *J* = 7.6 Hz, 2 H), 7.10 (t, *J* = 7.3 Hz, 1 H), 6.97 (t, *J* = 7.8 Hz, 1 H), 4.80 (dd, *J* = 11.2, 5.3 Hz, 1 H), 4.40 (t, *J* = 11.9 Hz, 1 H), 3.49–3.38 (m, 1 H), 2.89 (dd, *J* = 15.2, 5.6 Hz, 1 H), 2.52 (dd, *J* = 15.3, 6.2 Hz, 1 H); ^13^C-NMR (101 MHz, Chloroform-*d*) δ 193.2, 168.2, 157.3, 137.6, 136.3, 129.0, 125.9, 124.4, 122.7, 121.6, 121.6, 119.9, 70.9, 42.6, 33.4; HR-MS (ESI): *m/z* [M + H]^+^ calcd for C_17_H_15_ClNO_3_: 316.0735; found: 316.0740.

2-(7-chloro-4-oxochroman-3-yl)-*N*-phenylacetamide (**3ja**):

^1^H-NMR (400 MHz, Chloroform-*d*) δ 8.03 (s, 1 H), 7.81 (d, *J* = 8.8 Hz, 1 H), 7.50 (d, *J* = 7.8 Hz, 2 H), 7.30 (t, *J* = 7.4 Hz, 2 H), 7.10 (t, *J* = 7.3 Hz, 1 H), 7.00 (d, *J* = 6.6 Hz, 2 H), 4.68 (dd, *J* = 11.1, 5.3 Hz, 1 H), 4.31 (t, *J* = 11.9 Hz, 1 H), 3.44–3.36 (m, 1 H), 2.90 (dd, *J* = 15.2, 5.5 Hz, 1 H), 2.48 (dd, *J* = 15.2, 6.1 Hz, 1 H); ^13^C-NMR (100 MHz, Chloroform-*d*) δ 193.1, 168.4, 162.2, 142.2, 137.6, 129.0, 128.5, 124.4, 122.4, 119.8, 118.9, 118.1, 70.7, 42.7, 33.4; HR-MS (ESI): *m/z* [M + H]^+^ calcd for C_17_H_15_ClNO_3_: 316.0735; found: 316.0737.

2-(5-chloro-4-oxochroman-3-yl)-*N*-phenylacetamide (**3ka**):

^1^H-NMR (400 MHz, Chloroform-*d*) δ 8.06 (s, 1 H), 7.51 (d, *J* = 8.0 Hz, 2 H), 7.36–7.27 (m, 3 H), 7.09 (t, *J* = 7.3 Hz, 1 H), 7.03 (d, *J* = 7.8 Hz, 1 H), 6.90 (d, *J* = 8.4 Hz, 1 H), 4.67 (dd, *J* = 11.2, 5.4 Hz, 1 H), 4.30 (t, *J* = 12.0 Hz, 1 H), 3.49–3.40 (m, 1 H), 2.91 (dd, *J* = 15.1, 5.9 Hz, 1 H), 2.48 (dd, *J* = 15.1, 6.1 Hz, 1 H); ^13^C-NMR (100 MHz, Chloroform-*d*) δ 192.2, 168.5, 163.2, 137.7, 135.0, 134.4, 128.9, 124.7, 124.4, 119.9, 117.7, 117.0, 70.0, 43.5, 33.4; HR-MS (ESI): *m/z* [M + H]^+^ calcd for C_17_H_15_ClNO_3_: 316.0735; found: 316.0743.

2-(7-bromo-4-oxochroman-3-yl)-*N*-phenylacetamide (**3la**):

^1^H-NMR (400 MHz, Chloroform-*d*) δ 7.98 (s, 1 H), 7.73 (d, *J* = 8.4 Hz, 1 H), 7.50 (d, *J* = 7.8 Hz, 2 H), 7.31 (t, *J* = 7.6 Hz, 2 H), 7.22–7.06 (m, 3 H), 4.68 (dd, *J* = 11.1, 5.4 Hz, 1 H), 4.31 (t, *J* = 11.9 Hz, 1 H), 3.44–3.36 (m, 1 H), 2.90 (dd, *J* = 15.2, 5.6 Hz, 1 H), 2.48 (dd, *J* = 15.2, 6.1 Hz, 1 H); ^13^C-NMR (100 MHz, Chloroform-*d*) δ 193.2, 168.3, 162.0, 137.6, 130.8, 129.0, 128.5, 125.2, 124.5, 121.1, 119.8, 119.3, 70.7, 42.7, 33.4; HR-MS (ESI): *m/z* [M + H]^+^ calcd for C_17_H_15_ClNO_3_: 316.0735; found: 316.0739.

2-(6-bromo-4-oxochroman-3-yl)-*N*-phenylacetamide (**3ma**):

^1^H-NMR (400 MHz, Chloroform-*d*) δ 7.99 (s, 1 H), 7.91 (s, 1 H), 7.56 (d, *J* = 8.7 Hz, 1 H), 7.31 (t, *J* = 7.6 Hz, 2 H), 7.11 (t, *J* = 7.3 Hz, 1 H), 6.89 (d, *J* = 8.8 Hz, 1 H), 4.70 (dd, *J* = 11.2, 5.3 Hz, 1 H), 4.31 (t, *J* = 12.0 Hz, 1 H), 3.46–3.35 (m, 1 H), 2.91 (dd, *J* = 15.2, 5.5 Hz, 1 H), 2.50 (dd, *J* = 15.2, 6.3 Hz, 1 H); ^13^C-NMR (100 MHz, Chloroform-*d*) δ 192.9, 168.2, 160.8, 138.9, 137.6, 129.7, 129.0, 124.5, 121.6, 120.0, 119.8, 114.2, 70.5, 42.7, 33.4; HR-MS (ESI): *m/z* [M + H]^+^ calcd for C_17_H_15_BrNO_3_: 360.0230; found: 360.0227.

methyl 4-oxo-3-(2-oxo-2-(phenylamino)ethyl)chromane-6-carboxylate (**3na**):

^1^H-NMR (400 MHz, Chloroform-*d*) δ 8.57 (s, 1 H), 8.14 (d, *J* = 8.7 Hz, 1 H), 8.05 (s, 1 H), 7.51 (d, *J* = 7.9 Hz, 2 H), 7.30 (t, *J* = 7.7 Hz, 2 H), 7.10 (t, *J* = 7.4 Hz, 1 H), 7.02 (d, *J* = 8.7 Hz, 1 H), 4.76 (dd, *J* = 11.3, 5.5 Hz, 1 H), 4.36 (t, *J* = 12.0 Hz, 1 H), 3.90 (s, 3 H), 3.49–3.40 (m, 1 H), 2.95 (dd, *J* = 15.3, 5.5 Hz, 1 H), 2.51 (dd, *J* = 15.3, 6.4 Hz, 1 H); ^13^C-NMR (100 MHz, Chloroform-*d*) δ 193.1, 168.3, 165.9, 164.9, 137.6, 136.9, 129.8, 129.0, 124.4, 123.7, 119.8, 118.3, 70.6, 52.2, 42.7, 33.3; HR-MS (ESI): *m/z* [M + H]^+^ calcd for C_19_H_18_NO_5_: 340.1179; found: 340.1172.

2-(1-oxo-2,3-dihydro-1H-benzo[f]chromen-2-yl)-*N*-phenylacetamide (**3oa**):

^1^H-NMR (400 MHz, Chloroform-*d*) δ 9.42 (d, *J* = 8.7 Hz, 1 H), 8.21 (s, 1 H), 7.94 (d, *J* = 9.0 Hz, 1 H), 7.76 (d, *J* = 8.0 Hz, 1 H), 7.64 (t, *J* = 7.7 Hz, 1 H), 7.55 (d, *J* = 7.9 Hz, 2 H), 7.44 (t, *J* = 7.5 Hz, 1 H), 7.32 (t, *J* = 7.5 Hz, 2 H), 7.10 (d, *J* = 8.8 Hz, 2 H), 4.76 (dd, *J* = 11.1, 5.4 Hz, 1 H), 4.43 (t, *J* = 11.8 Hz, 1 H), 3.54–3.45 (m, 1 H), 2.95 (dd, *J* = 14.9, 6.2 Hz, 1 H), 2.55 (dd, *J* = 14.8, 5.6 Hz, 1 H); ^13^C-NMR (100 MHz, Chloroform-*d*) δ 195.1, 168.9, 164.1, 138.0, 137.8, 131.5, 129.8, 129.2, 129.0, 128.5, 125.6, 125.0, 124.3, 119.8, 118.6, 111.9, 70.4, 43.4, 34.2; HR-MS (ESI): *m/z* [M + H]^+^ calcd for C_21_H_18_NO_3_: 332.1281; found: 332.1278.

2-(3-methyl-4-oxochroman-3-yl)-*N*-phenylacetamide (**3pa**):

^1^H-NMR (400 MHz, Chloroform-*d*) δ 8.33 (s, 1 H), 7.92 (d, *J* = 7.8 Hz, 1 H), 7.51 (t, *J* = 6.9 Hz, 3 H), 7.30 (t, *J* = 7.7 Hz, 2 H), 7.07 (dt, *J* = 15.4, 7.4 Hz, 2 H), 6.99 (d, *J* = 8.4 Hz, 1 H), 4.53 (d, *J* = 11.6 Hz, 1 H), 4.33 (d, *J* = 11.6 Hz, 1 H), 2.78 (d, *J* = 14.1 Hz, 1 H), 2.56 (d, *J* = 14.1 Hz, 1 H), 1.37 (s, 3 H); ^13^C-NMR (100 MHz, Chloroform-*d*) δ 197.5, 167.8, 161.2, 137.7, 136.4, 128.9, 127.9, 124.3, 121.7, 119.8, 119.2, 117.8, 74.6, 44.3, 41.5, 19.5; HR-MS (ESI): *m/z* [M + H]^+^ calcd for C_18_H_18_NO_3_: 296.1281; found: 296.1276.

2-(1-oxo-2,3-dihydro-1H-inden-2-yl)-*N*-phenylacetamide (**3qa**):

^1^H-NMR (400 MHz, Chloroform-*d*) δ 8.28 (s, 1 H), 7.77 (d, *J* = 7.6 Hz, 1 H), 7.61 (t, *J* = 7.4 Hz, 1 H), 7.52 (d, *J* = 7.9 Hz, 2 H), 7.46 (d, *J* = 7.7 Hz, 1 H), 7.38 (t, *J* = 7.6 Hz, 1 H), 7.29 (t, *J* = 7.8 Hz, 2 H), 7.08 (t, *J* = 7.0 Hz, 1 H), 3.51 (dd, *J* = 17.1, 8.0 Hz, 1 H), 3.14 (dt, *J* = 12.6, 6.7 Hz, 1 H), 3.00 (dt, *J* = 17.4, 4.9 Hz, 2 H), 2.65 (dd, *J* = 15.3, 7.2 Hz, 1 H); ^13^C-NMR (100 MHz, Chloroform-*d*) δ 208.4, 169.4, 153.6, 137.9, 136.0, 135.2, 129.7, 128.9, 127.6, 126.6, 124.0, 119.8, 44.3, 38.3, 33.3; HR-MS (ESI): *m/z* [M + H]^+^ calcd for C_17_H_16_NO_2_: 266.1176; found: 266.1182.

2-(4-oxochroman-3-yl)-*N*-(p-tolyl)acetamide (**3ab**):

^1^H-NMR (400 MHz, Chloroform-*d*) δ 7.95 (s, 1 H), 7.90 (d, *J* = 7.9 Hz, 1 H), 7.49 (t, *J* = 7.7 Hz, 1 H), 7.39 (d, *J* = 8.2 Hz, 2 H), 7.11 (d, *J* = 8.0 Hz, 2 H), 7.07–6.95 (m, 2 H), 4.68 (dd, *J* = 11.2, 5.4 Hz, 1 H), 4.31 (t, *J* = 11.9 Hz, 1 H), 3.41 (dq, *J* = 11.8, 5.7 Hz, 1 H), 2.90 (dd, *J* = 15.0, 5.8 Hz, 1 H), 2.48 (dd, *J* = 15.0, 6.1 Hz, 1 H), 2.30 (s, 3 H); ^13^C-NMR (100 MHz, Chloroform-*d*) δ 194.2, 168.4, 161.9, 136.3, 135.2, 134.0, 129.4, 127.4, 121.5, 120.4, 119.9, 117.9, 70.5, 43.0, 33.7, 20.8; HR-MS (ESI): *m/z* [M + H]^+^ calcd for C_18_H_18_NO_3_: 296.1281; found: 296.1280.

*N*-(4-methoxyphenyl)-2-(4-oxochroman-3-yl)acetamide (**3ac**):

^1^H-NMR (400 MHz, Chloroform-*d*) δ 7.96 (s, 1 H), 7.89 (d, *J* = 7.8 Hz, 1 H), 7.48 (d, *J* = 7.2 Hz, 1 H), 7.41 (d, *J* = 8.9 Hz, 2 H), 7.05–6.95 (m, 2 H), 6.84 (d, *J* = 8.9 Hz, 2 H), 4.68 (dd, *J* = 11.3, 5.4 Hz, 1 H), 4.31 (t, *J* = 11.9 Hz, 1 H), 3.78 (s, 3 H), 3.44–3.36 (m, 1 H), 2.89 (dd, *J* = 15.0, 5.9 Hz, 1 H), 2.47 (dd, *J* = 15.0, 6.2 Hz, 1 H); ^13^C-NMR (101 MHz, Chloroform-*d*) δ 194.2, 168.3, 161.9, 156.4, 136.3, 130.8, 127.4, 121.7, 121.5, 120.4, 117.9, 114.1, 70.5, 55.5, 43.0, 33.5; HR-MS (ESI): *m/z* [M + H]^+^ calcd for C_18_H_18_NO_4_: 312.1230; found: 312.1235.

*N*-(4-fluorophenyl)-2-(4-oxochroman-3-yl)acetamide (**3ad**):

^1^H-NMR (400 MHz, Chloroform-*d*) δ 8.14 (s, 1 H), 7.89 (d, *J* = 7.8 Hz, 1 H), 7.61–7.38 (m, 3 H), 7.14–6.89 (m, 4 H), 4.67 (dd, *J* = 11.2, 5.4 Hz, 1 H), 4.31 (t, *J* = 11.9 Hz, 1 H), 3.46–3.35 (m, 1 H), 2.89 (dd, *J* = 15.0, 6.2 Hz, 1 H), 2.49 (dd, *J* = 15.0, 5.6 Hz, 1 H); ^13^C-NMR (100 MHz, Chloroform-*d*) δ 194.4, 168.6, 161.9, 159.3 (d, *J*_C-F_ = 243.0 Hz), 136.5, 133.7 (d, *J*_C-F_ = 3.0 Hz), 127.4, 121.7, 121.6, 120.3, 118.0, 115.6 (d, *J*_C-F_ = 22.0 Hz), 70.5, 43.0, 33.7; ^19^F-NMR (376 MHz, Chloroform-*d*) δ −117.9; HR-MS (ESI): *m/z* [M + H]^+^ calcd for C_17_H_15_FNO_3_: 300.1030; found: 300.1034.

*N*-(4-chlorophenyl)-2-(4-oxochroman-3-yl)acetamide (**3ae**):

^1^H-NMR (400 MHz, Chloroform-*d*) δ 8.24 (s, 1 H), 7.90 (d, *J* = 7.8 Hz, 1 H), 7.52–7.45 (m, 3 H), 7.31–7.25 (m, 2 H), 7.04 (t, *J* = 7.5 Hz, 1 H), 6.99 (d, *J* = 8.4 Hz, 1 H), 4.67 (dd, *J* = 11.3, 5.4 Hz, 1H), 4.31 (t, *J* = 12.0 Hz, 1H), 3.46–3.34 (m, 1 H), 2.89 (dd, *J* = 15.0, 6.3 Hz, 1 H), 2.49 (dd, *J* = 15.0, 5.5 Hz, 1 H); ^13^C-NMR (100 MHz, Chloroform-*d*) δ 194.5, 168.6, 161.9, 136.5, 136.3, 129.3, 129.0, 127.4, 121.6, 121.0, 120.3, 118.0, 70.5, 42.9, 33.9; HR-MS (ESI): *m/z* [M + H]^+^ calcd for C_17_H_15_ClNO_3_: 316.0735; found: 316.0733.

*N*-(4-bromophenyl)-2-(4-oxochroman-3-yl)acetamide (**3af**):

^1^H-NMR (400 MHz, Chloroform-*d*) δ 8.19 (s, 1 H), 7.90 (d, *J* = 7.8 Hz, 1 H), 7.52–7.35 (m, 5 H), 7.14–6.91 (m, 2 H), 4.67 (dd, *J* = 11.2, 5.4 Hz, 1 H), 4.31 (t, *J* = 12.0 Hz, 1 H), 3.45–3.37 (m, 1 H), 2.88 (dd, *J* = 15.0, 6.4 Hz, 1 H), 2.49 (dd, *J* = 15.0, 5.3 Hz, 1 H); ^13^C-NMR (100 MHz, Chloroform-*d*) δ 194.5, 168.6, 161.9, 136.8, 136.6, 131.9, 127.4, 121.6, 121.3, 120.3, 118.0, 116.9, 70.5, 42.9, 34.0; HR-MS (ESI): *m/z* [M + H]^+^ calcd for C_17_H_15_BrNO_3_: 360.0230; found: 360.0234.

2-(4-oxochroman-3-yl)-*N*-(4-(trifluoromethyl)phenyl)acetamide (**3ag**):

^1^H-NMR (400 MHz, Chloroform-*d*) δ 8.50 (s, 1 H), 7.90 (d, *J* = 7.9 Hz, 1 H), 7.65 (d, *J* = 8.3 Hz, 2 H), 7.53–7.45 (m, 3 H), 7.09–6.95 (m, 2 H), 4.67 (dd, *J* = 11.2, 5.5 Hz, 1 H), 4.32 (t, *J* = 12.0 Hz, 1 H), 3.49–3.37 (m, 1 H), 2.91 (dd, *J* = 15.0, 6.5 Hz, 1 H), 2.52 (dd, *J* = 15.0, 5.2 Hz, 1 H); ^13^C NMR (100 MHz, Chloroform-*d*) δ 194.6, 168.9, 161.9, 140.8, 136.7, 127.4, 126.2 (q, *J*_C-F_ = 4.0 Hz), 125.8, 124.0 (q, *J*_C-F_ = 270.0 Hz), 121.7, 120.2, 119.3, 118.0, 70.4, 42.9, 34.0; ^19^F-NMR (376 MHz, Chloroform-*d*) δ −62.1; HR-MS (ESI): *m/z* [M + H]^+^ calcd for C_18_H_15_F_3_NO_3_: 350.0999; found: 350.0994.

*N*-(3-bromophenyl)-2-(4-oxochroman-3-yl)acetamide (**3ah**): 

^1^H-NMR (400 MHz, Chloroform-*d*) δ 8.27 (s, 1 H), 7.90 (d, *J* = 7.8 Hz, 1 H), 7.80 (s, 1 H), 7.51 (t, *J* = 7.7 Hz, 1 H), 7.43 (d, *J* = 7.8 Hz, 1 H), 7.30–7.16 (m, 2 H), 7.11–6.92 (m, 2 H), 4.67 (dd, *J* = 11.2, 5.4 Hz, 1 H), 4.31 (t, *J* = 12.0 Hz, 1 H), 3.44–3.35 (m, 1 H), 2.89 (dd, *J* = 15.0, 6.3 Hz, 1 H), 2.50 (dd, *J* = 14.9, 5.3 Hz, 1 H); ^13^C-NMR (100 MHz, Chloroform-*d*) δ 194.5, 168.7, 161.9, 139.0, 136.6, 130.2, 127.4, 127.3, 122.6, 122.6, 121.6, 120.2, 118.2, 118.0, 70.4, 42.9, 33.9; HR-MS (ESI): *m/z* [M+H]^+^ calcd for C_17_H_15_BrNO_3_: 360.0230; found: 360.0232.

*N*-benzyl-2-(4-oxochroman-3-yl)acetamide (**3ai**):

^1^H-NMR (400 MHz, Chloroform-*d*) δ 7.85 (d, *J* = 7.8 Hz, 1 H), 7.47 (t, *J* = 7.7 Hz, 1 H), 7.36–7.22 (m, 5 H), 7.06–6.88 (m, 2 H), 6.29 (s, 1 H), 4.64 (dd, *J* = 11.2, 5.3 Hz, 1 H), 4.44 (d, *J* = 5.7 Hz, 2 H), 4.27 (t, *J* = 11.7 Hz, 1 H), 3.39–3.31 (m, 1 H), 2.77 (dd, *J* = 15.1, 5.4 Hz, 1 H), 2.34 (dd, *J* = 15.1, 6.8 Hz, 1 H); ^13^C-NMR (100 MHz, Chloroform-*d*) δ 193.8, 170.1, 161.8, 138.0, 136.1, 128.7, 127.7, 127.5, 127.3, 121.4, 120.4, 117.9, 70.5, 43.7, 42.9, 32.4; HR-MS (ESI): *m/z* [M + H]^+^ calcd for C_18_H_18_NO_3_: 296.1281; found: 296.1288.

*N*-cyclohexyl-2-(4-oxochroman-3-yl)acetamide (**3aj**):

^1^H-NMR (400 MHz, Chloroform-*d*) δ 7.87 (d, *J* = 7.8 Hz, 1 H), 7.47 (t, *J* = 7.8 Hz, 1 H), 7.12–6.86 (m, 2 H), 5.83 (s, 1 H), 4.64 (dd, *J* = 11.3, 5.2 Hz, 1 H), 4.26 (t, *J* = 11.7 Hz, 1 H), 3.91–3.66 (m, 1 H), 3.43–3.23 (m, 1 H), 2.70 (dd, *J* = 14.9, 5.3 Hz, 1 H), 2.29 (dd, *J* = 15.0, 6.9 Hz, 1 H), 1.75 - 1.54 (m, 4 H), 1.43–1.08 (m, 6 H); ^13^C-NMR (100 MHz, Chloroform-*d*) δ 193.9, 169.2, 161.8, 136.1, 127.3, 121.4, 120.4, 117.9, 70.5, 48.4, 43.0, 33.1, 33.0, 32.7, 25.5; HR-MS (ESI): *m/z* [M + H]^+^ calcd for C_17_H_22_NO_3_: 288.1594; found: 288.1586.

*N*-cyclopentyl-2-(4-oxochroman-3-yl)acetamide (**3ak**):

^1^H-NMR (400 MHz, Chloroform-*d*) δ 7.87 (d, *J* = 7.8 Hz, 1 H), 7.48 (t, *J* = 7.8 Hz, 1 H), 7.11–6.87 (m, 2 H), 5.93 (s, 1 H), 4.64 (dd, *J* = 11.3, 5.3 Hz, 1 H), 4.26 (t, *J* = 11.7 Hz, 1 H), 4.18 (q, *J* = 6.9 Hz, 1 H), 3.36–3.26 (m, 1 H), 2.70 (dd, *J* = 15.0, 5.5 Hz, 1 H), 2.28 (dd, *J* = 15.0, 6.8 Hz, 1 H), 1.97 (dd, *J* = 11.3, 4.5 Hz, 2 H), 1.84–1.61 (m, 4 H), 1.45–1.31 (m, 2 H); ^13^C-NMR (100 MHz, Chloroform-*d*) δ 193.9, 169.7, 161.8, 136.1, 127.3, 121.4, 120.4, 117.9, 70.5, 51.3, 43.0, 33.1, 33.0, 32.6, 23.7; HR-MS (ESI): *m/z* [M + H]^+^ calcd for C_16_H_20_NO_3_: 274.1438; found: 274.1433.

*N*-butyl-2-(4-oxochroman-3-yl)acetamide (**3al**):

^1^H-NMR (400 MHz, Chloroform-*d*) δ 7.87 (d, *J* = 7.8 Hz, 1 H), 7.48 (t, *J* = 7.6 Hz, 1 H), 7.09–6.93 (m, 2 H), 5.93 (s, 1 H), 4.64 (dd, *J* = 11.2, 5.2 Hz, 1 H), 4.27 (t, *J* = 11.7 Hz, 1 H), 3.33 (dt, *J* = 12.0, 5.9 Hz, 1 H), 3.25 (q, *J* = 6.7 Hz, 2 H), 2.72 (dd, *J* = 15.0, 5.4 Hz, 1 H), 2.30 (dd, *J* = 15.0, 6.8 Hz, 1 H), 1.53–1.44 (m, 2 H), 1.39–1.28 (m, 2 H), 0.92 (t, *J* = 7.3 Hz, 3 H); ^13^C-NMR (100 MHz, Chloroform-*d*) δ 193.9, 170.1, 161.8, 136.1, 127.3, 121.4, 120.4, 117.9, 70.5, 43.0, 39.4, 32.6, 31.6, 20.0, 13.7; HR-MS (ESI): *m/z* [M + H]^+^ calcd for C_15_H_20_NO_3_: 262.1238; found: 262.1442.

3-(2-morpholino-2-oxoethyl)chroman-4-one (**3am**):

^1^H-NMR (400 MHz, Chloroform-d) δ 7.89 (d, *J* = 7.8 Hz, 1 H), 7.48 (t, *J* = 7.7 Hz, 1 H), 7.08–6.92 (m, 2 H), 4.67 (dd, *J* = 11.0, 5.2 Hz, 1 H), 4.31 (t, *J* = 11.3 Hz, 1 H), 3.76–3.38 (m, 9 H), 3.01 (dd, *J* = 16.6, 3.5 Hz, 1 H), 2.35 (dd, *J* = 16.6, 8.6 Hz, 1 H); ^13^C-NMR (100 MHz, Chloroform-d) δ 193.6, 168.7, 161.8, 136.0, 127.3, 121.4, 120.6, 117.9, 70.7, 66.8, 66.5, 45.9, 42.6, 42.1, 28.8; HR-MS (ESI): *m/z* [M + H]^+^ calcd for C_15_H_18_NO_4_: 276.1230; found: 276.1237.

N-(adamantan-1-yl)-2-(4-oxochroman-3-yl)acetamide (**3an**):

^1^H-NMR (400 MHz, Chloroform-d) δ 7.87 (d, *J* = 7.9 Hz, 1 H), 7.47 (t, *J* = 7.7 Hz, 1 H), 7.08–6.81 (m, 2 H), 5.57 (s, 1 H), 4.63 (dd, *J* = 11.2, 5.2 Hz, 1 H), 4.27 (t, *J* = 11.6 Hz, 1 H), 3.34–3.22 (m, 1 H), 2.65 (dd, *J* = 15.0, 5.1 Hz, 1 H), 2.23 (dd, *J* = 14.9, 7.1 Hz, 1 H), 2.14–1.93 (m, 9 H), 1.82–1.51 (m, 6 H); ^13^C-NMR (100 MHz, Chloroform-d) δ 193.9, 169.2, 161.8, 136.0, 127.3, 121.4, 120.5, 117.8, 70.5, 52.1, 43.1, 41.5, 36.3, 33.5, 29.4; HR-MS (ESI): *m/z* [M + H]^+^ calcd for C_21_H_26_NO_3_: 340.1907; found: 340.1904.

## 4. Conclusions

In summary, we developed a convenient and straightforward decarboxylative radical cascade cyclization of 2-(allyloxy)arylaldehydes and oxamic acids, leading to biological carbamoylated chroman-4-one scaffolds. The present reaction has the advantages of a readily available substrate, metal-free conditions, operational simplicity, a broad substrate scope, and favorable functional group compatibility, thus providing an attractive and practical approach for the synthesis of amide-functionalized chroman-4-ones.

## Figures and Tables

**Table 1:** Optimization of reaction conditions *^a^*.

Entry	Oxidant	Solvent	Temp	Yield of 3aa *^b^*
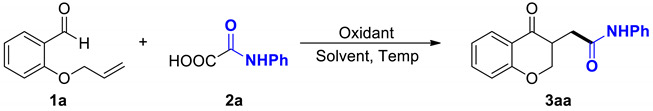				
1	(NH_4_)_2_S_2_O_8_	DMSO	70	78%
2	(NH_4_)_2_S_2_O_8_	CH_3_CN	70	0%
3	(NH_4_)_2_S_2_O_8_	DMF	70	0%
4	(NH_4_)_2_S_2_O_8_	DCE	70	0%
5	(NH_4_)_2_S_2_O_8_	THF	70	0%
6	(NH_4_)_2_S_2_O_8_	H_2_O	70	0%
7	(NH_4_)_2_S_2_O_8_	Acetone	70	0%
8	K_2_S_2_O_8_	DMSO	70	0%
9	Na_2_S_2_O_8_	DMSO	70	0%
10	TBHP	DMSO	70	0%
11	Selectfluor	DMSO	70	0%
12	PhI(OAc)_2_	DMSO	70	0%
13	(NH_4_)_2_S_2_O_8_	DMSO	60	81%
14	(NH_4_)_2_S_2_O_8_	DMSO	50	53%
15	(NH_4_)_2_S_2_O_8_	DMSO	80	72%
16 *^c^*	(NH_4_)_2_S_2_O_8_	DMSO	60	67%
17 *^d^*	(NH_4_)_2_S_2_O_8_	DMSO	60	71%
18 *^e^*	(NH_4_)_2_S_2_O_8_	DMSO	60	68%
19		DMSO	60	0%

*^a^* General conditions, unless otherwise noted: **1a** (0.3 mmol, 1 equiv.), **2a** (0.9 mmol, 3 equiv.), oxidant (1.2 mmol, 4 equiv.), solvent (2 mL), under N_2_ atmosphere for 12 h. *^b^* Isolated yields. *^c^* Oxidant (3.0 equiv.). *^d^* 2 eqiv. of **2a** was used. *^e^* under air conditions.

## Data Availability

The data presented in this study are available on request from the corresponding author.

## Supplementary Materials

The following supporting information can be downloaded at: https://www.mdpi.com/article/10.3390/molecules27207049/s1, Copies of the ^1^H-NMR and ^13^C-NMR for compounds **3aa**–**3qa** and **3ab**–**3an** can be found in Supplementary Materials.

## Sample Availability

Not available.
