# Supplementary material for: Metal-Free Synthesis of Carbamoylated Chroman-4-Ones via Cascade Radical Annulation of 2-(Allyloxy)arylaldehydes with Oxamic Acids
Source: Molecules. 2022 Oct 19;27(20):7049. doi: 10.3390/molecules27207049 (PMC9609457; doi:10.3390/molecules27207049)
Supplement: Supplementary file 1 [file molecules-27-07049-s001.zip › molecules-1940470-supplementary.pdf]

Metal Free Synthesis of Carbamoylated Chroman-4-Ones via Cascade Radical Annulation of 2-(Allyloxy)arylaldehydes with Oxamic Acids

Long-Yong Xie [1]*, Sha Peng [1], Li-Hua Yang [1], and Xiao-Wen Liu [1]*

College of Chemistry and Bioengineering, Hunan University of Science and Engineering, Yongzhou 425100, China

E-mail: xielongyong@huse.edu.cn

^{1}H and ^{13}C NMR spectra of products

2-(4-oxochroman-3-yl)-*N*-phenylacetamide (3aa)

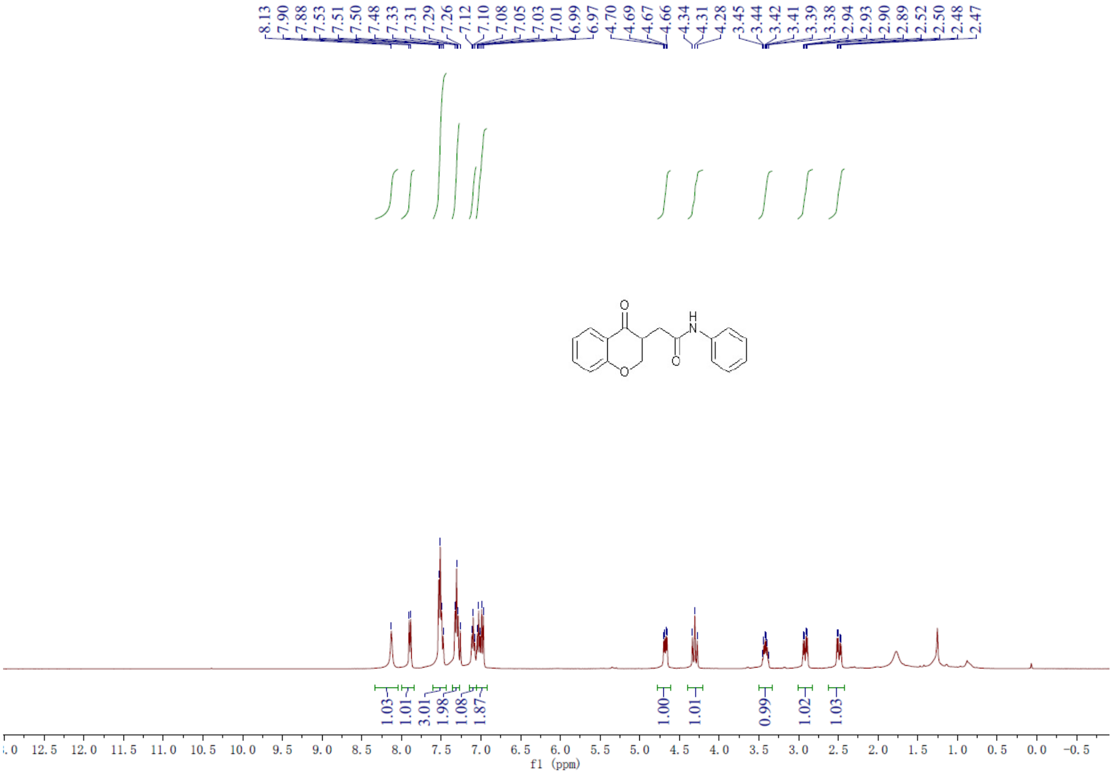

^{1}H spectra of **3aa**

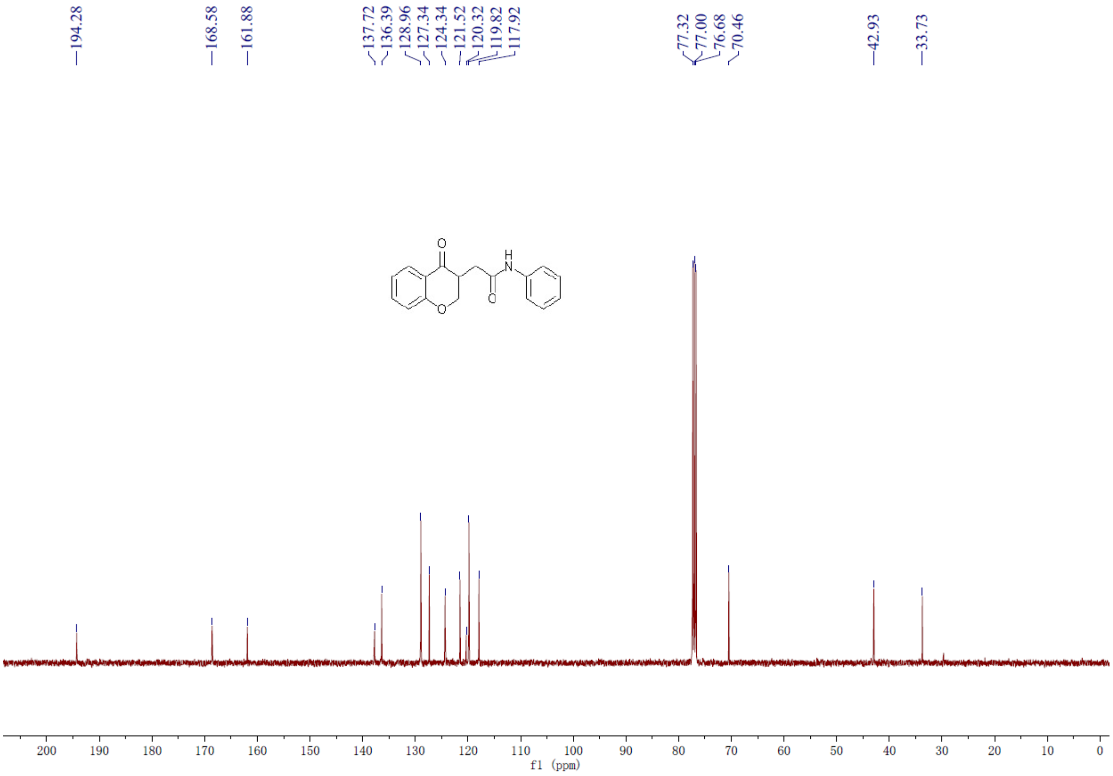

^{13}C spectra of **3aa**

2-(8-methyl-4-oxochroman-3-yl)-*N*-phenylacetamide (3ba)

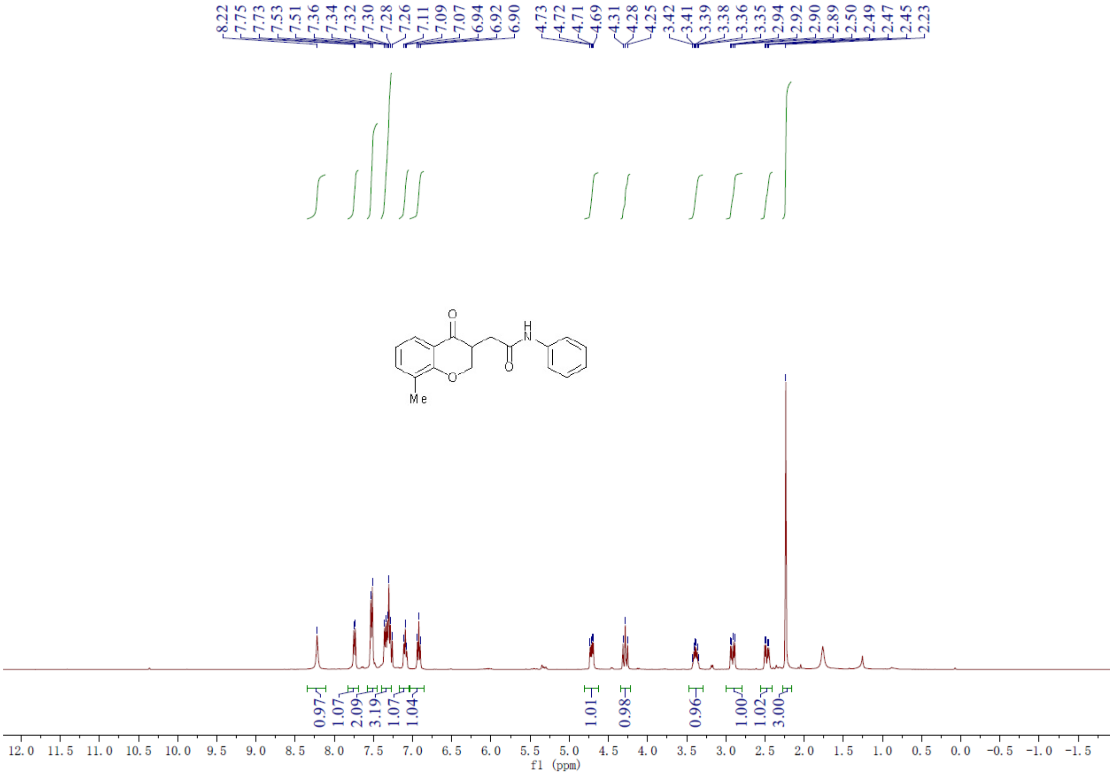

^{1}H spectra of **3ba**

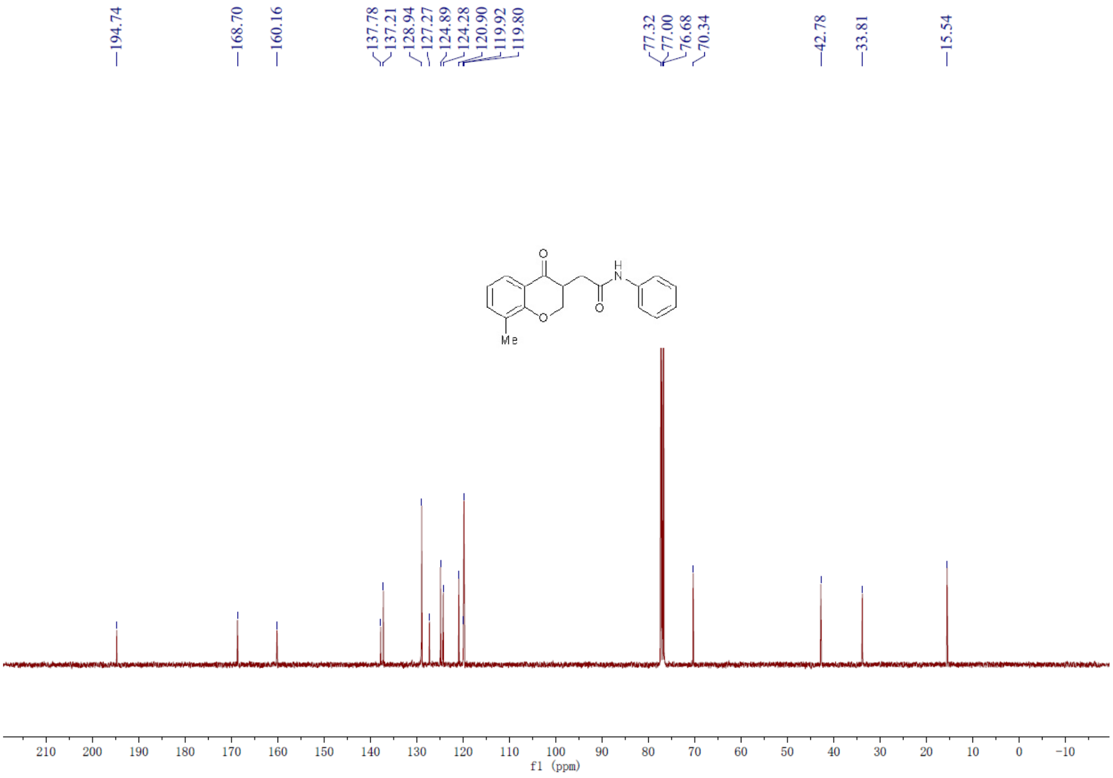

^{13}C spectra of **3ba**

2-(6-methyl-4-oxochroman-3-yl)-*N*-phenylacetamide (3ca)

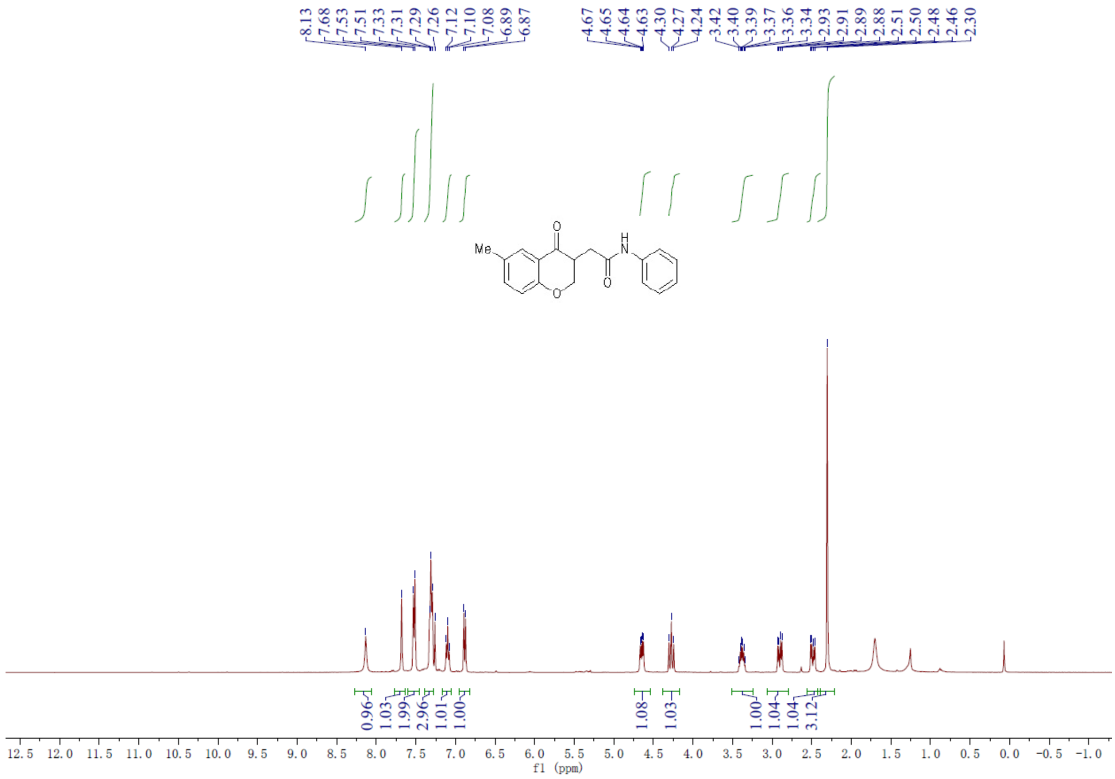

^{1}H spectra of **3ca**

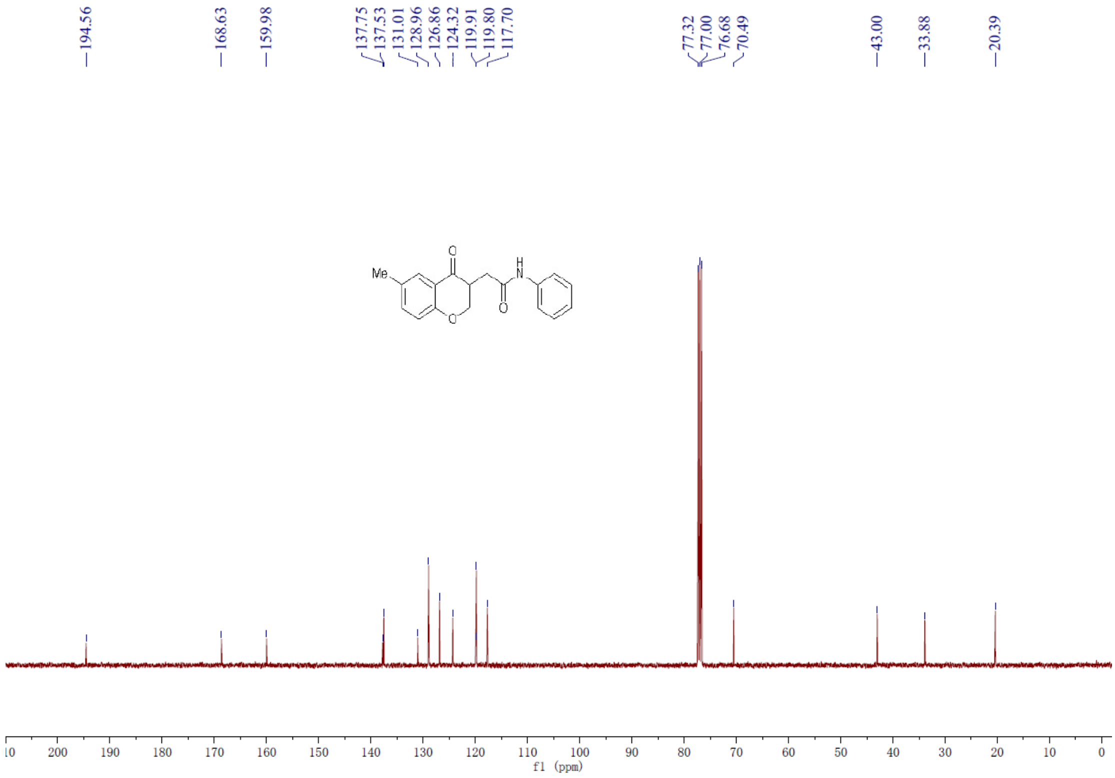

^{13}C spectra of **3ca**

2-(7-methoxy-4-oxochroman-3-yl)-*N*-phenylacetamide (3da)

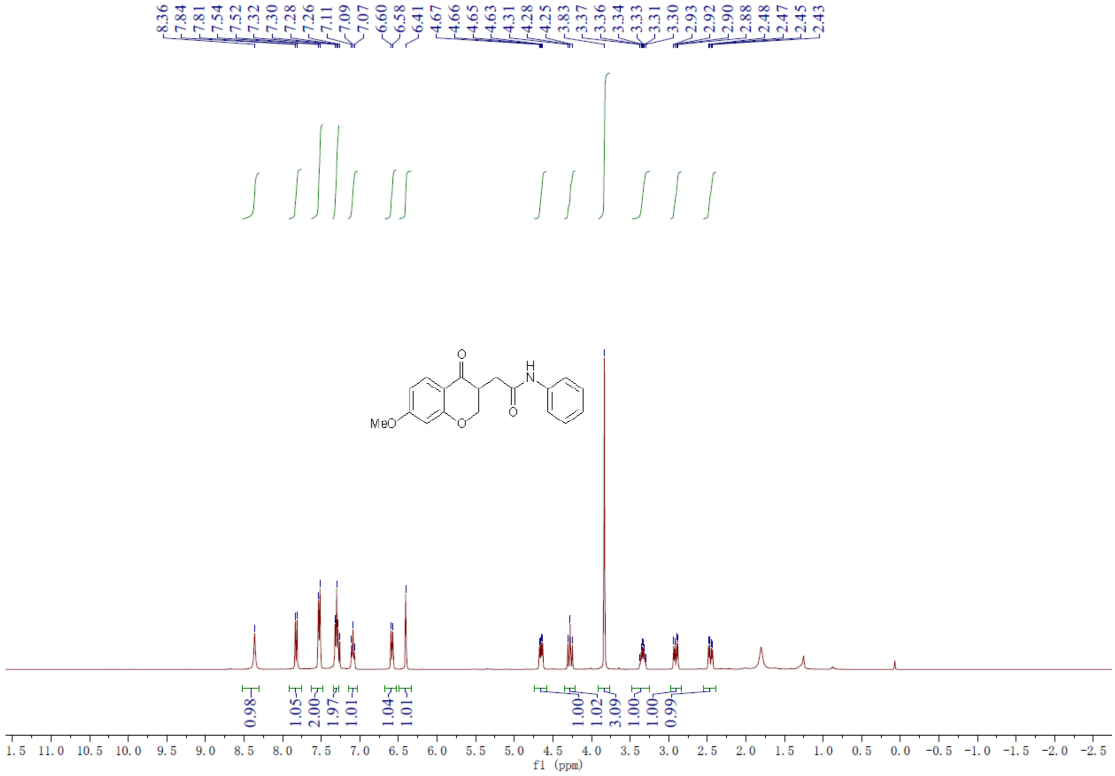

^{1}H spectra of **3da**

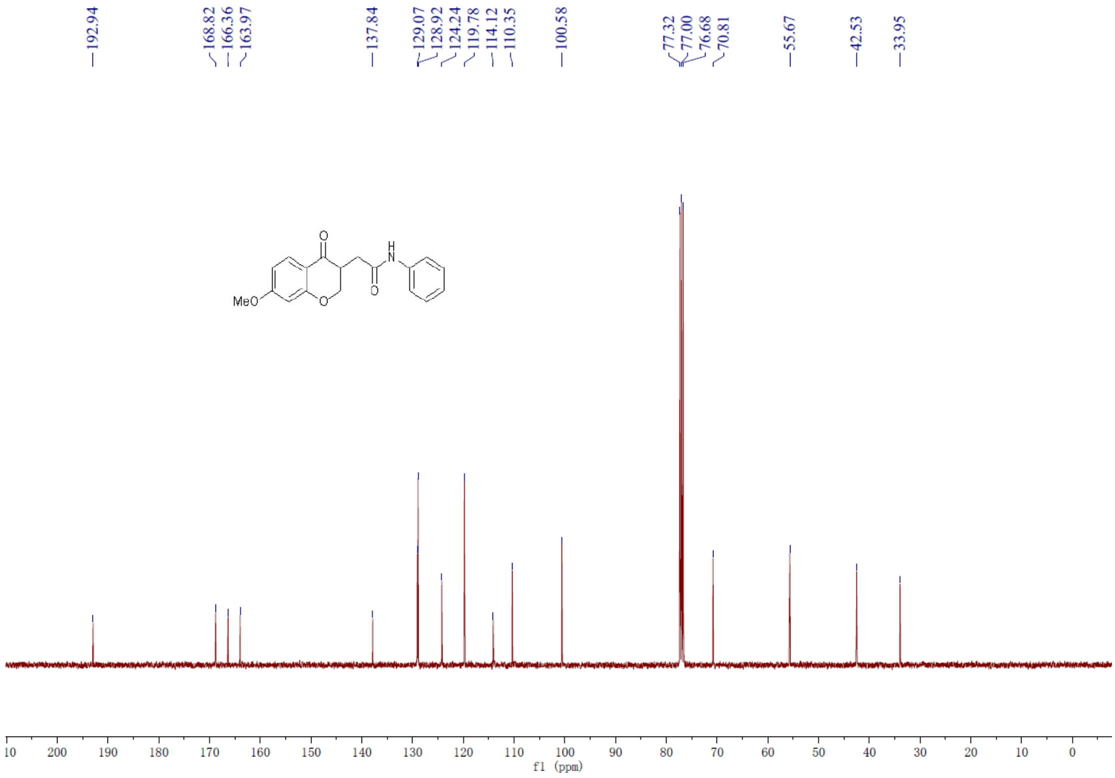

^{13}C spectra of **3da**

2-(6-methoxy-4-oxochroman-3-yl)-*N*-phenylacetamide (3ea)

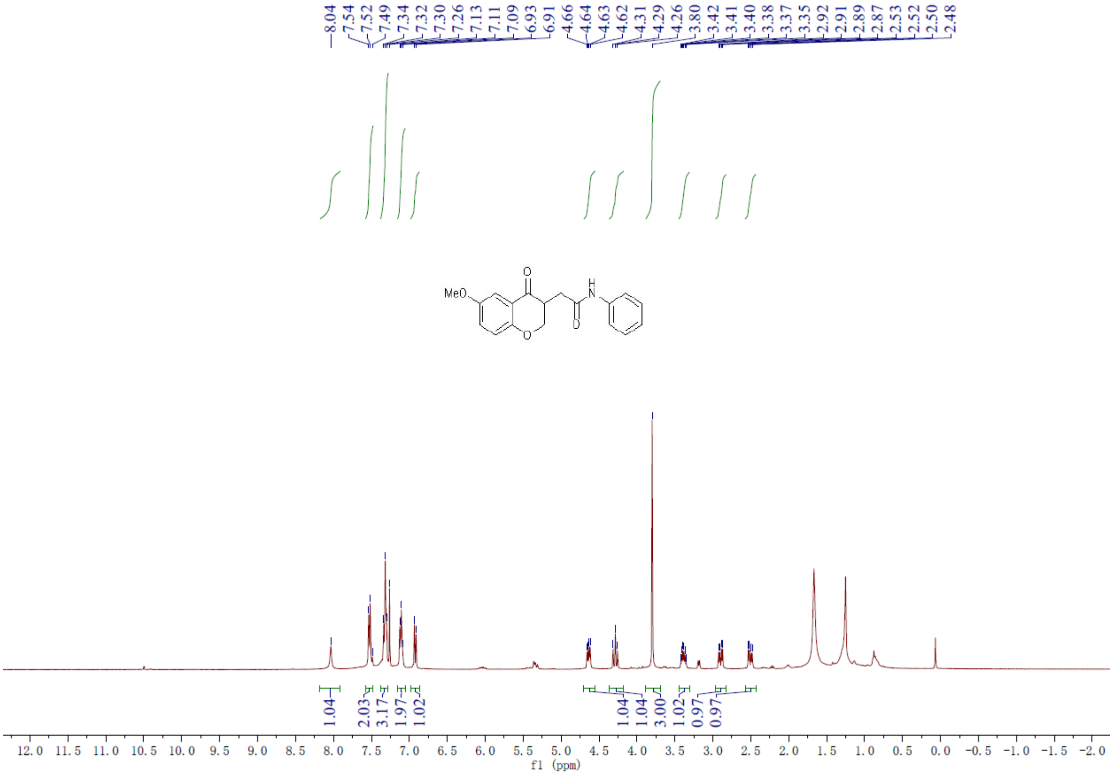

^{1}H spectra of **3ea**

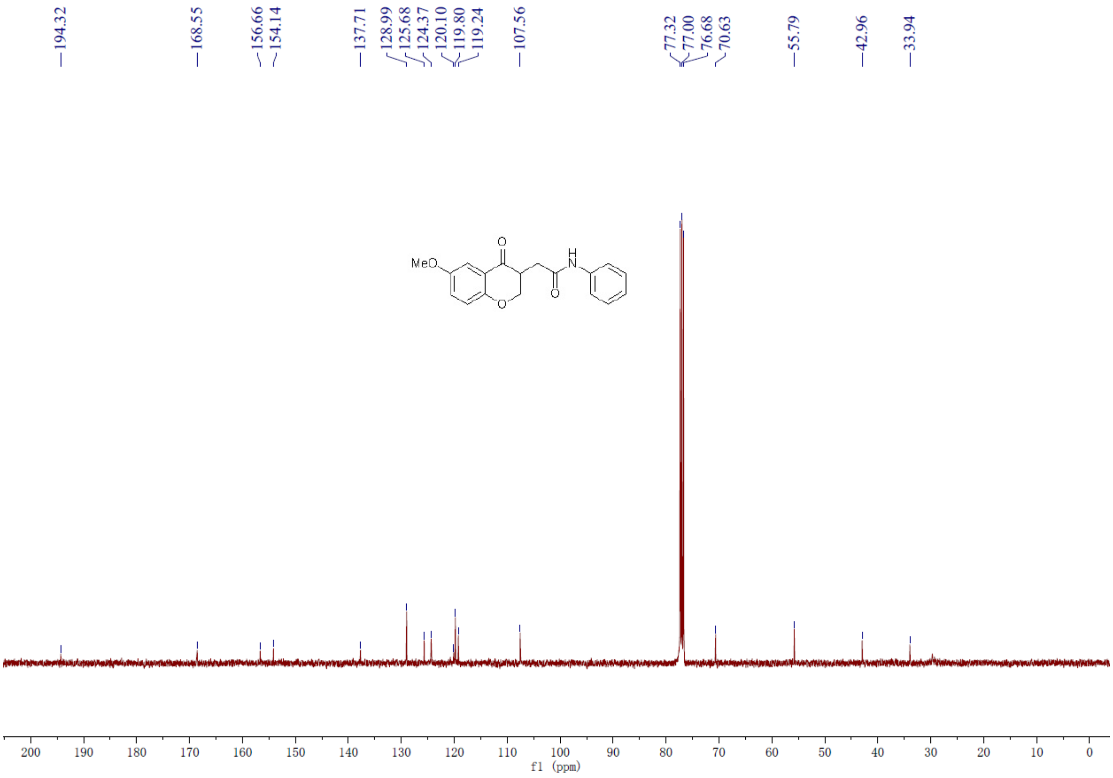

^{13}C spectra of **3ea**

2-(8-(tert-butyl)-4-oxochroman-3-yl)-*N*-phenylacetamide (3fa)

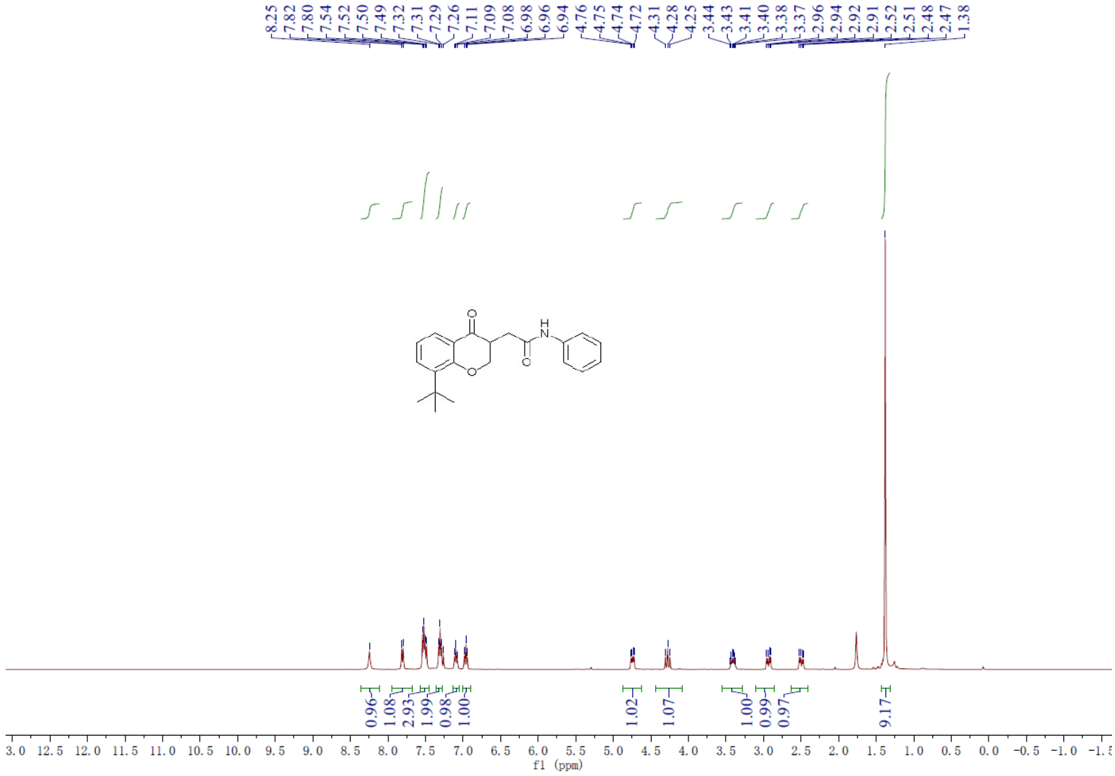

^{1}H spectra of **3fa**

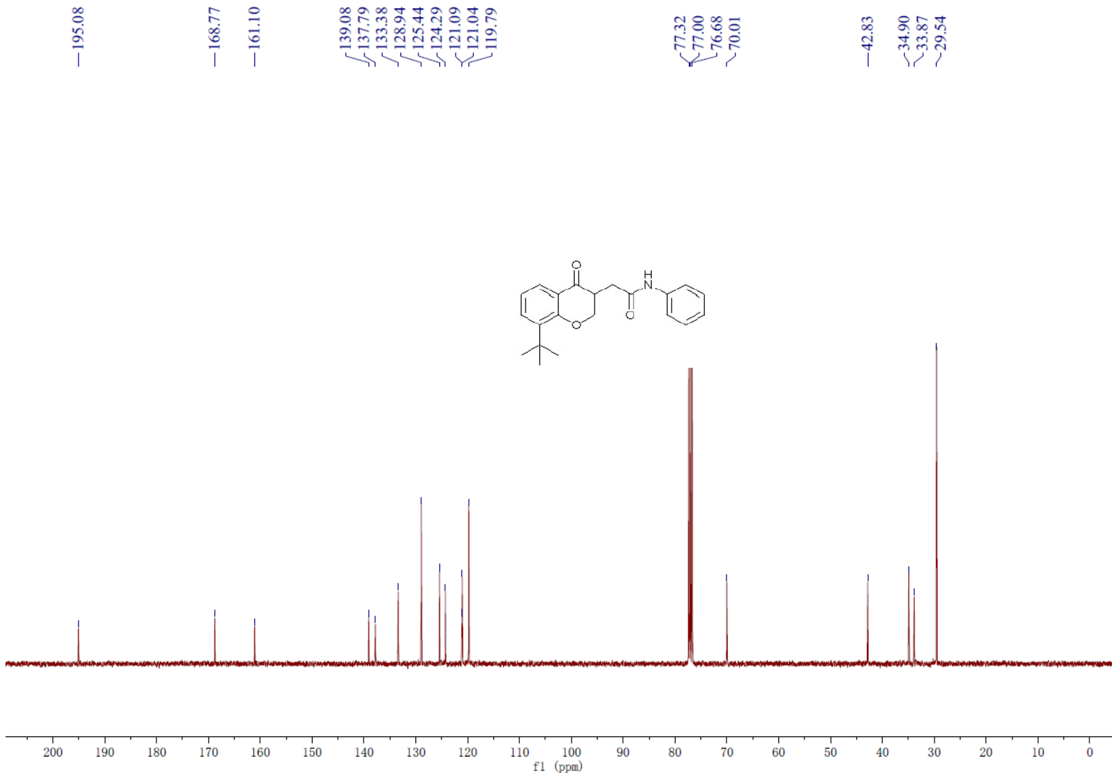

^{13}C spectra of **3fa**

2-(7-fluoro-4-oxochroman-3-yl)-*N*-phenylacetamide (3ga)

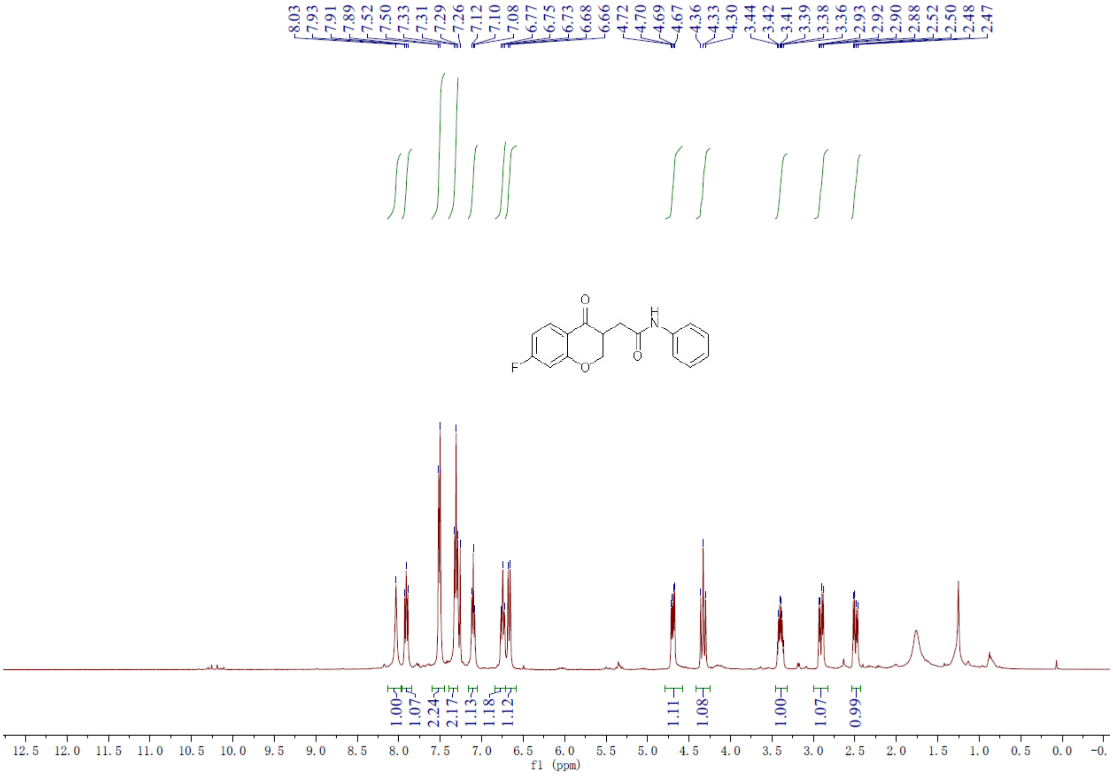

^{1}H spectra of **3ga**

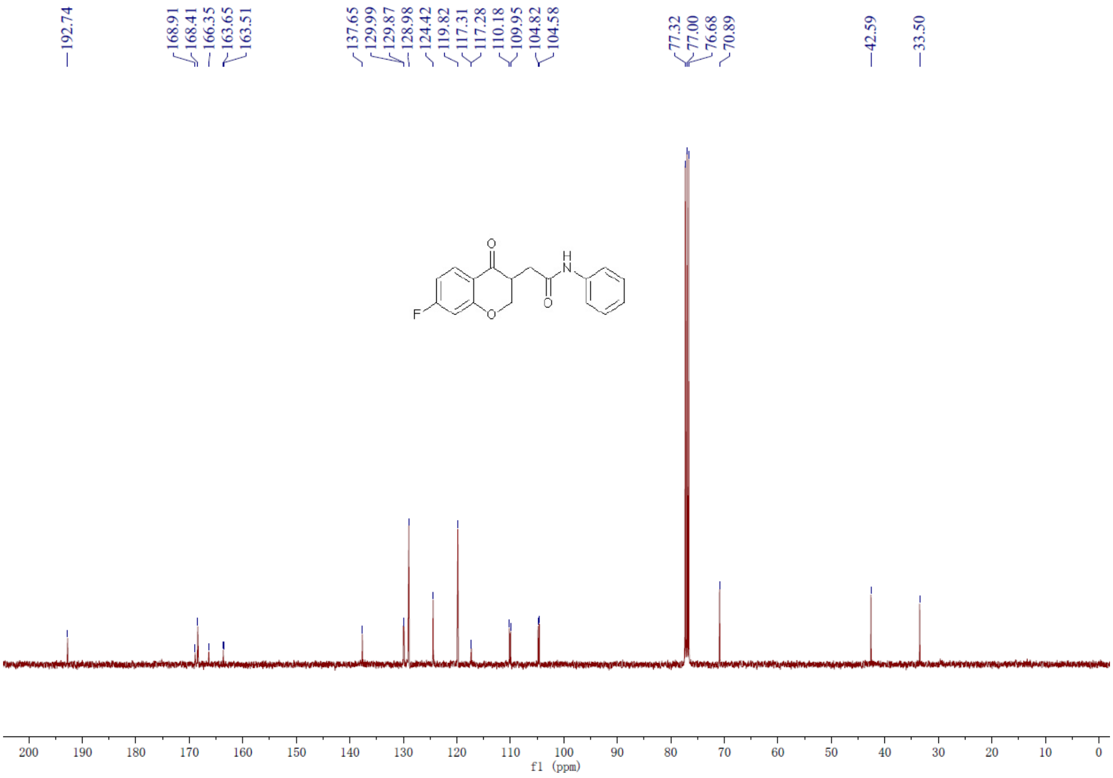

^{13}C spectra of **3ga**

2-(6-fluoro-4-oxochroman-3-yl)-*N*-phenylacetamide (3ha)

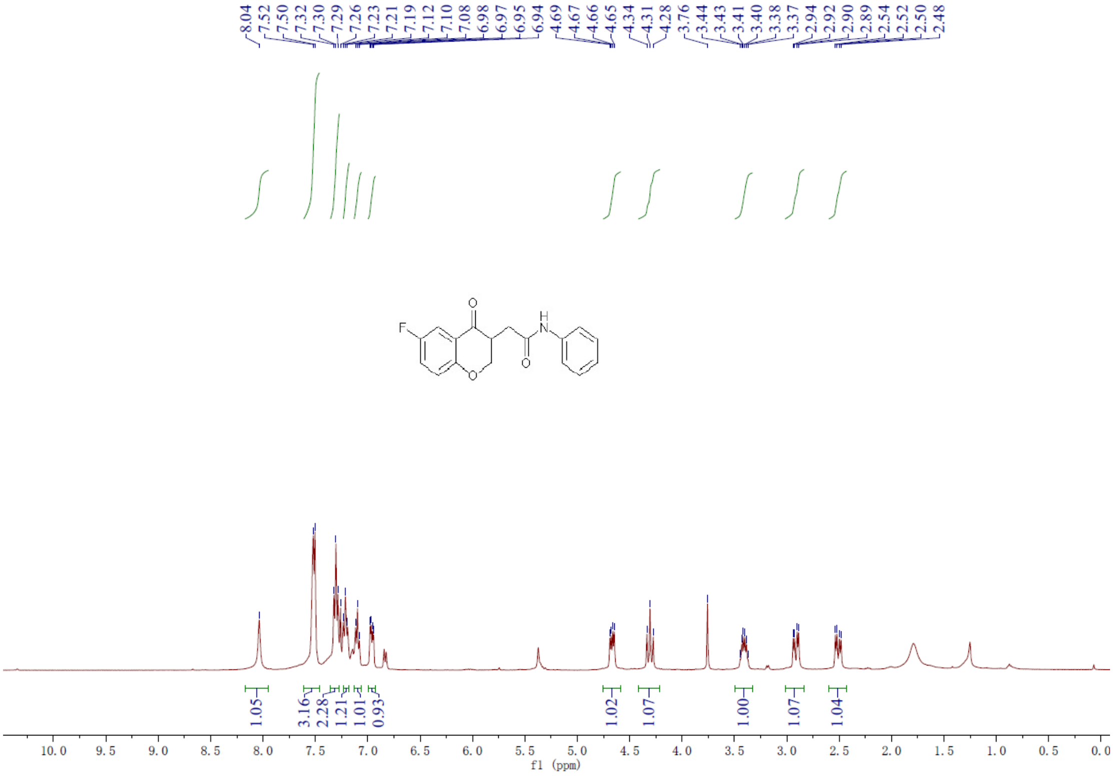

^{1}H spectra of **3ha**

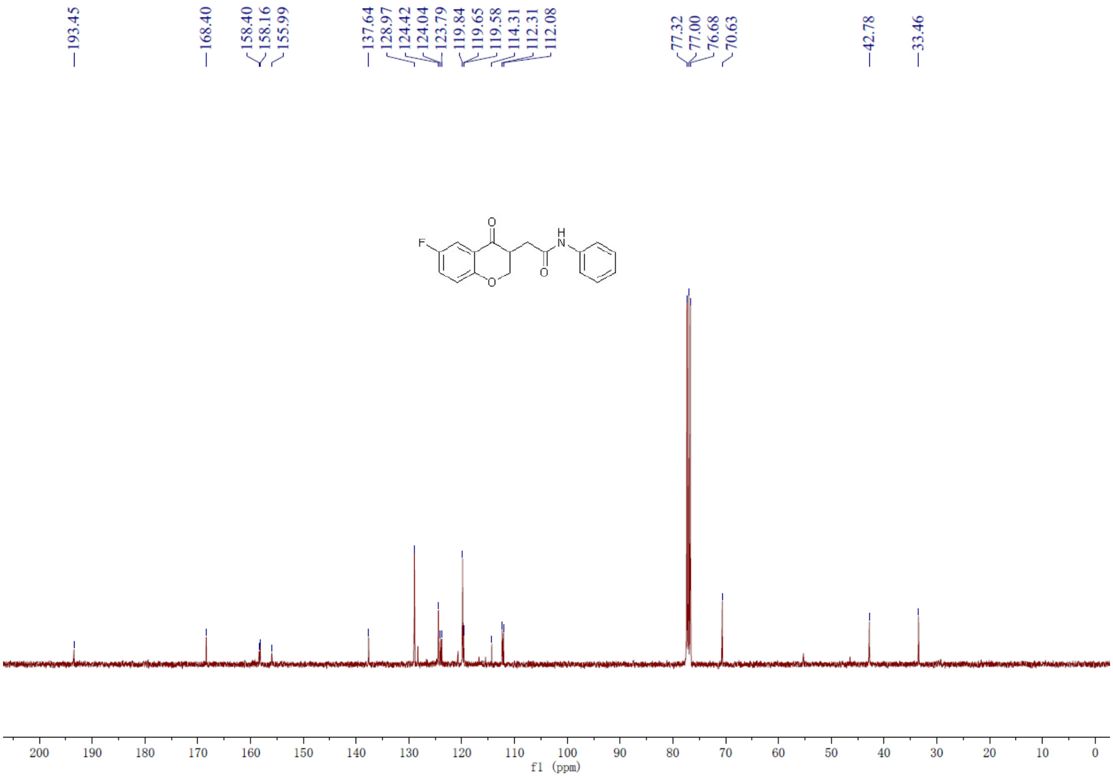

^{13}C spectra of **3ha**

2-(8-chloro-4-oxochroman-3-yl)-*N*-phenylacetamide (3ia)

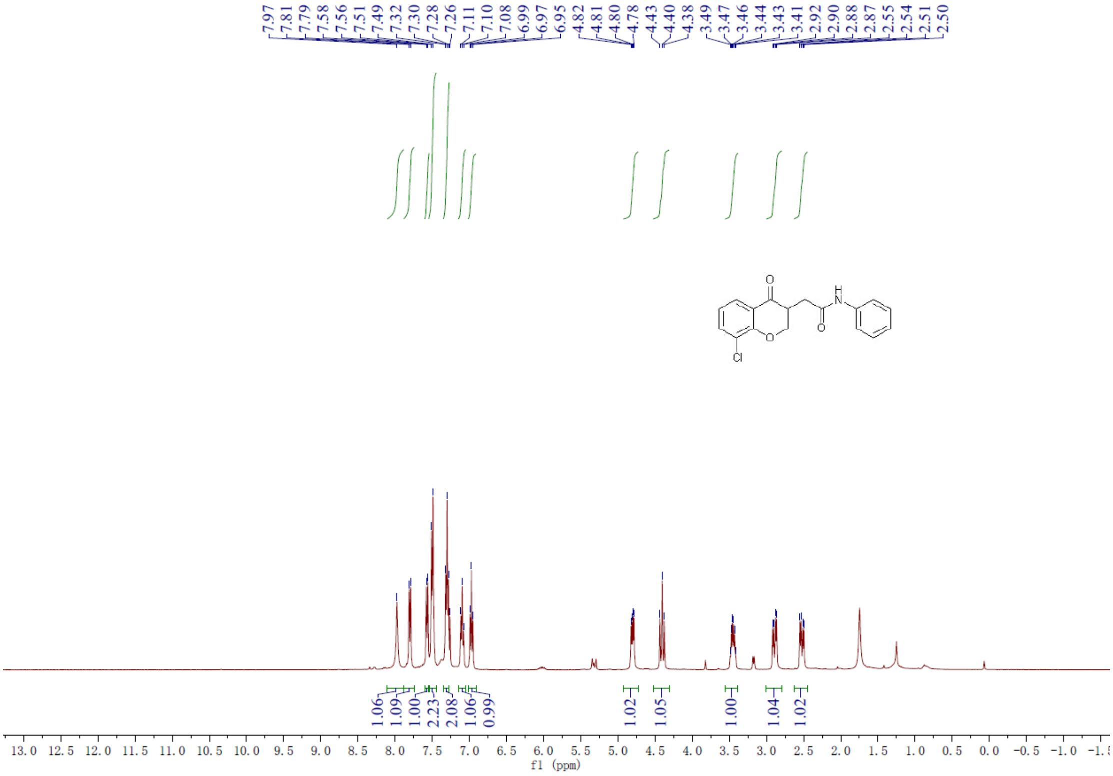

^{1}H spectra of **3ia**

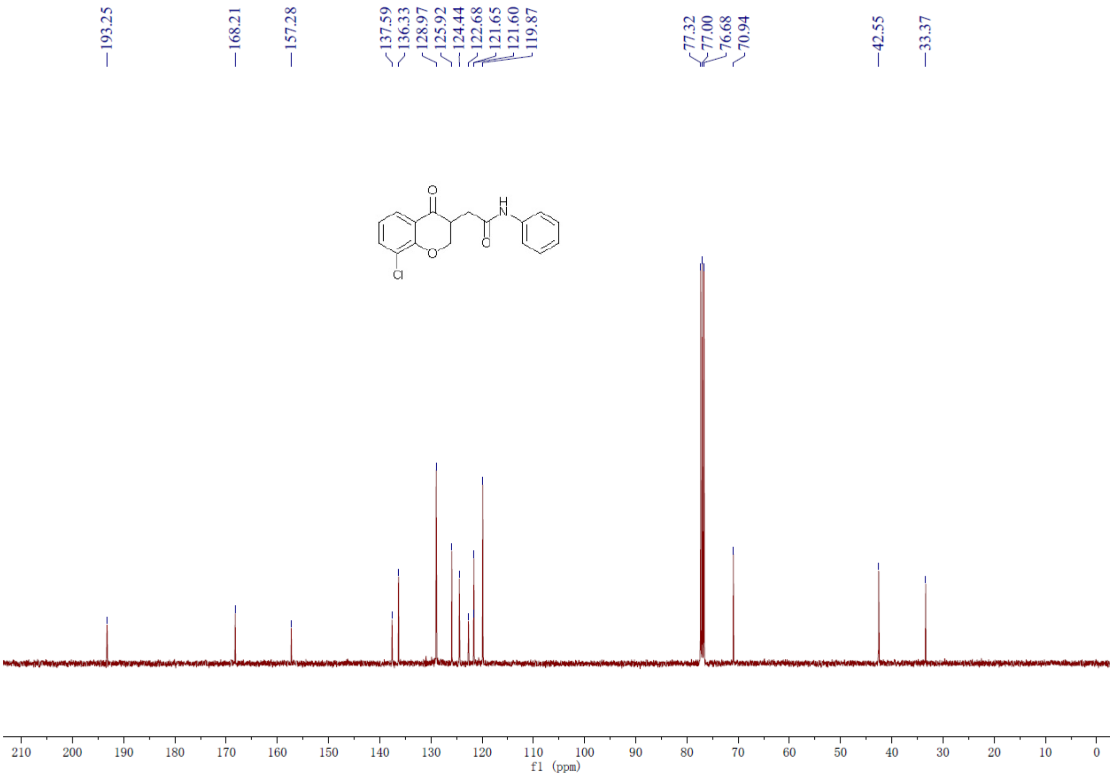

^{13}C spectra of **3ia**

2-(7-chloro-4-oxochroman-3-yl)-*N*-phenylacetamide (3ja)

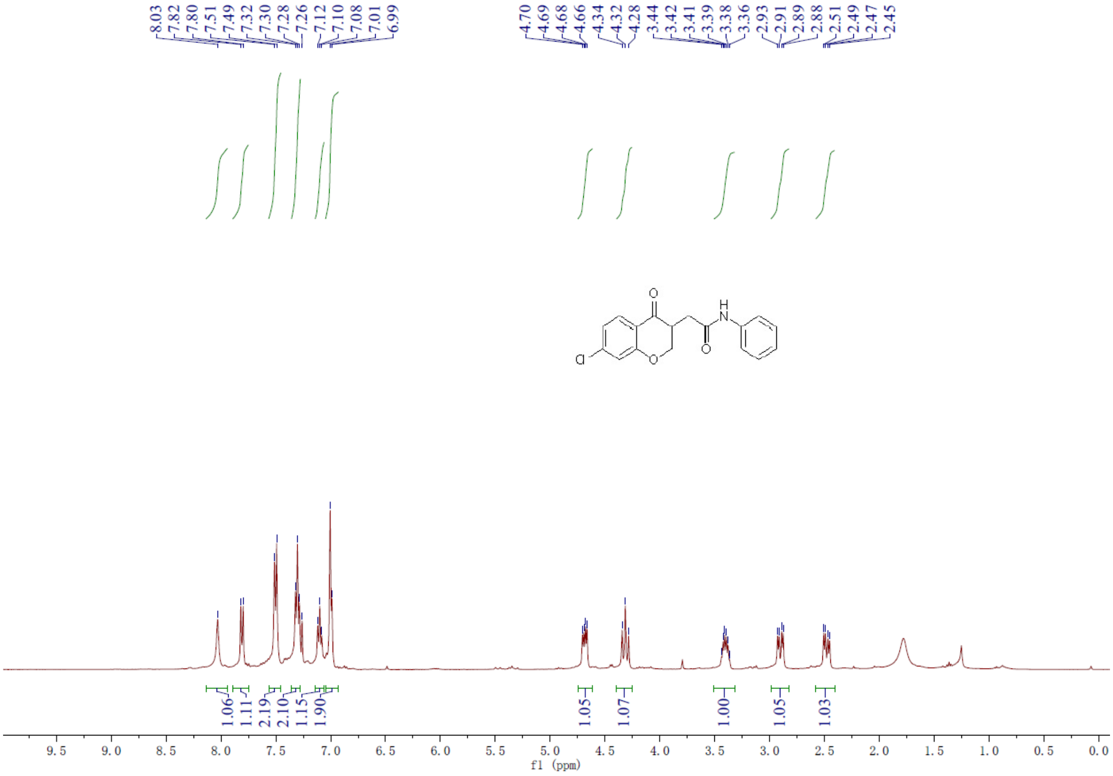

^{1}H spectra of **3ja**

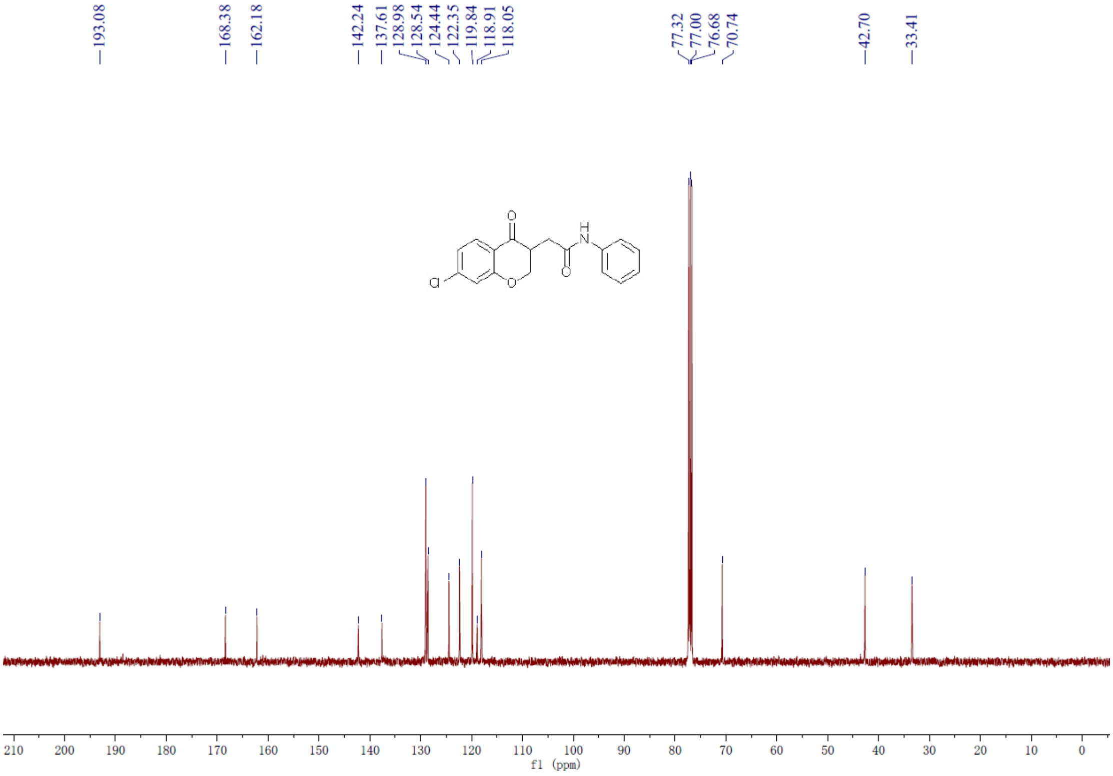

^{13}C spectra of **3ja**

2-(5-chloro-4-oxochroman-3-yl)-*N*-phenylacetamide (3ka)

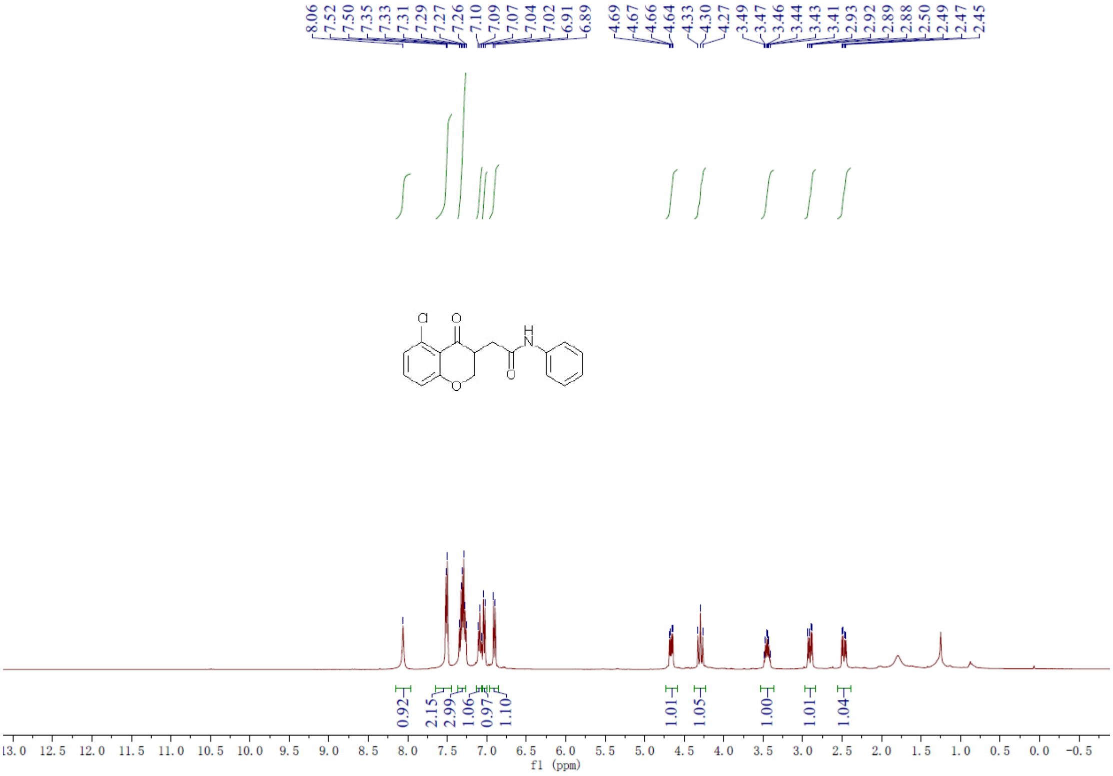

^{1}H spectra of **3ka**

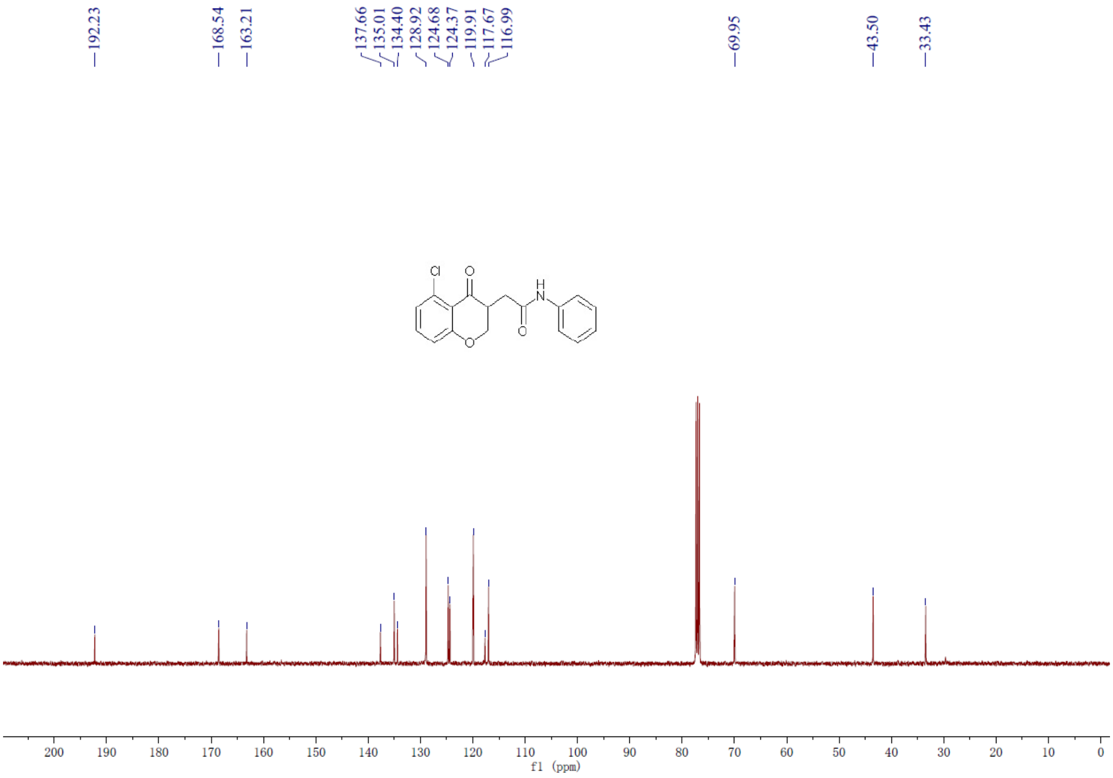

^{13}C spectra of **3ka**

2-(7-bromo-4-oxochroman-3-yl)-*N*-phenylacetamide (3la)

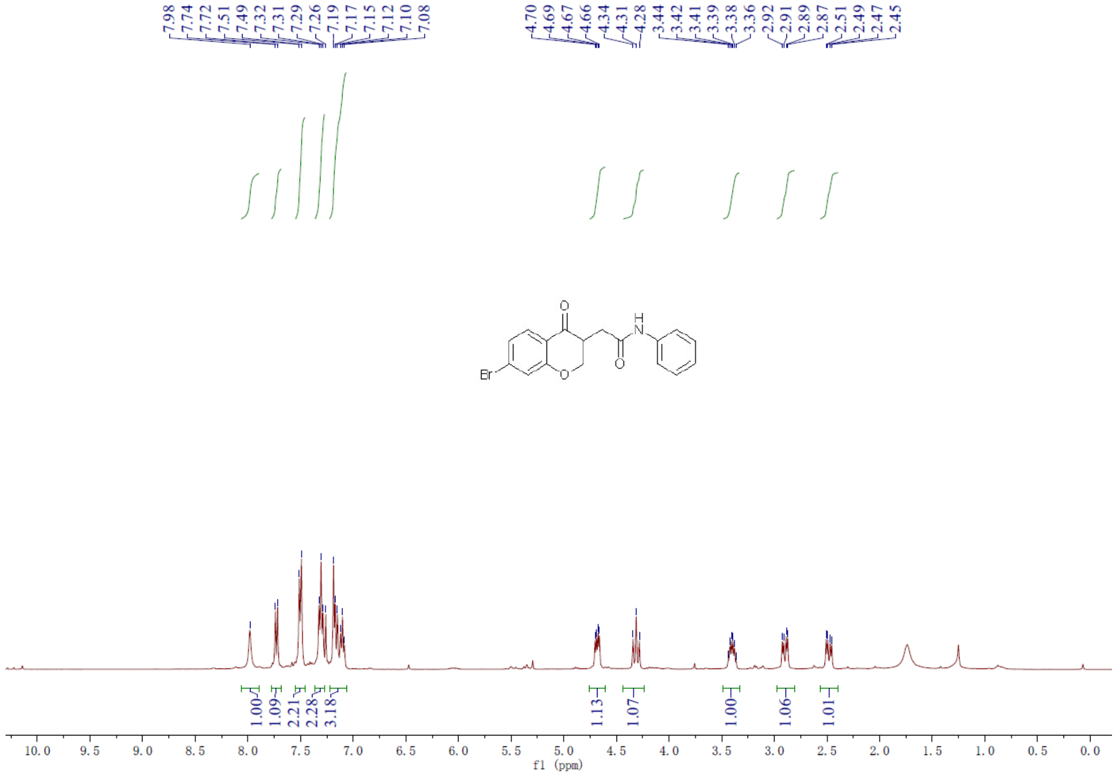

^{1}H spectra of **3la**

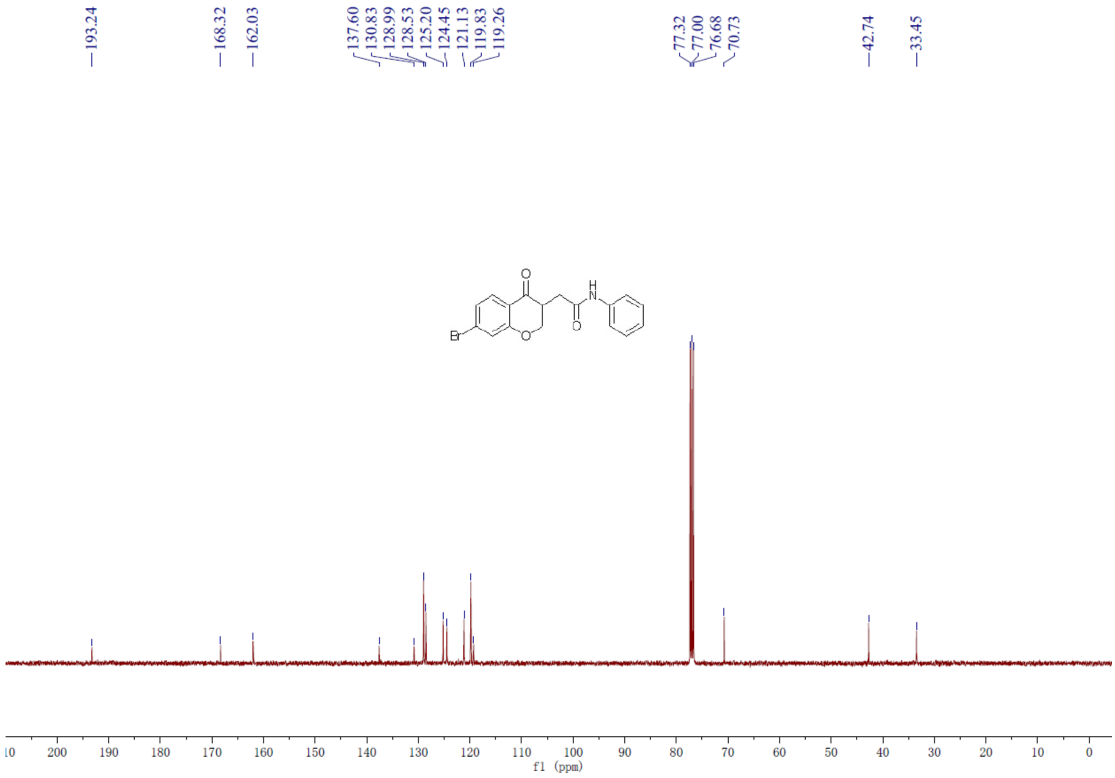

^{13}C spectra of **3la**

2-(6-bromo-4-oxochroman-3-yl)-*N*-phenylacetamide (3ma)

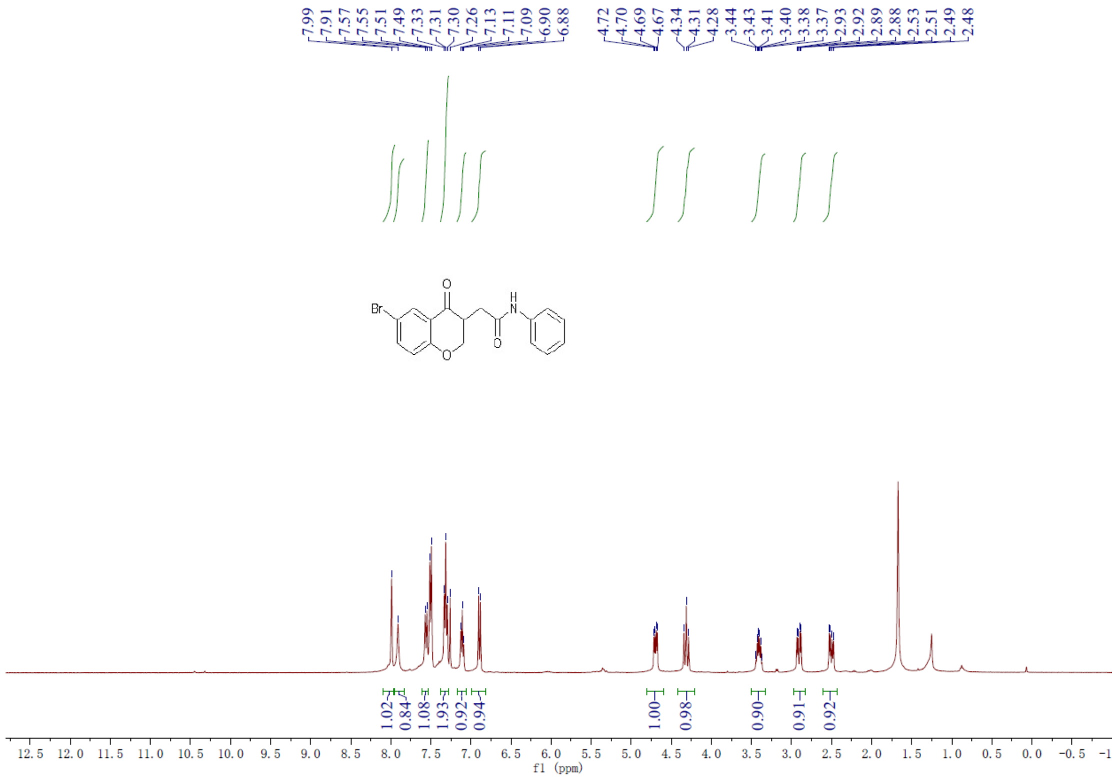

^{1}H spectra of **3ma**

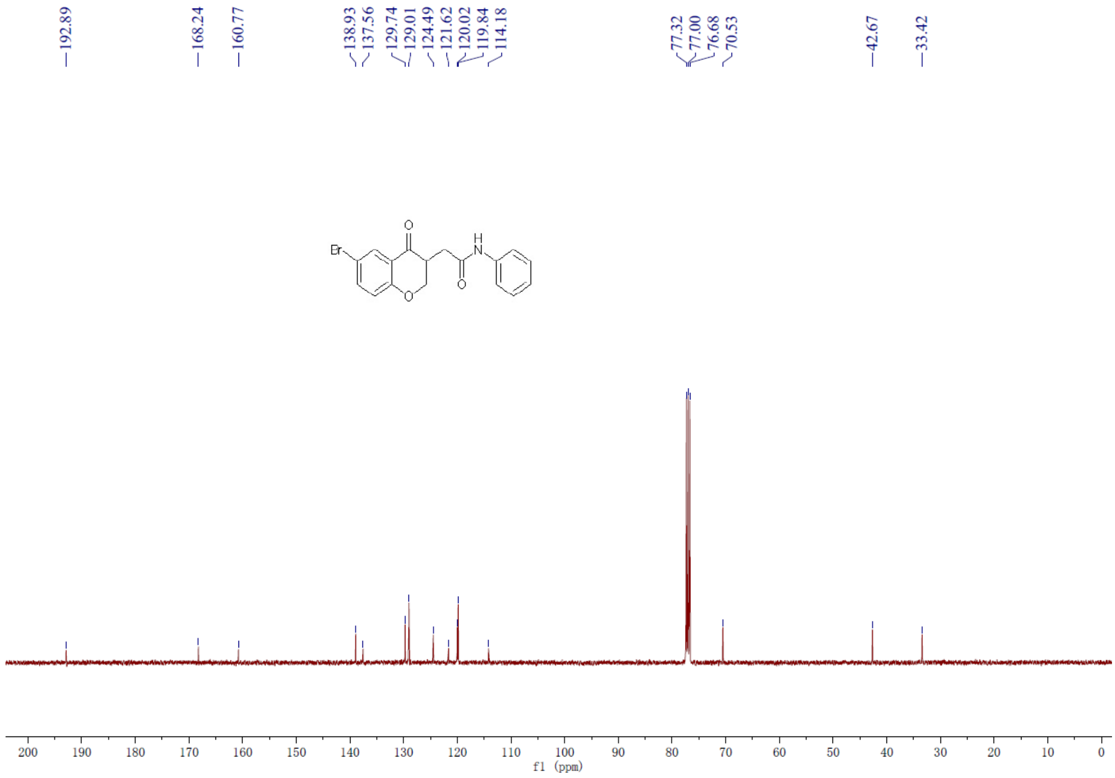

^{13}C spectra of **3ma**

methyl 4-oxo-3-(2-oxo-2-(phenylamino)ethyl)chroman-6-carboxylate (3na)

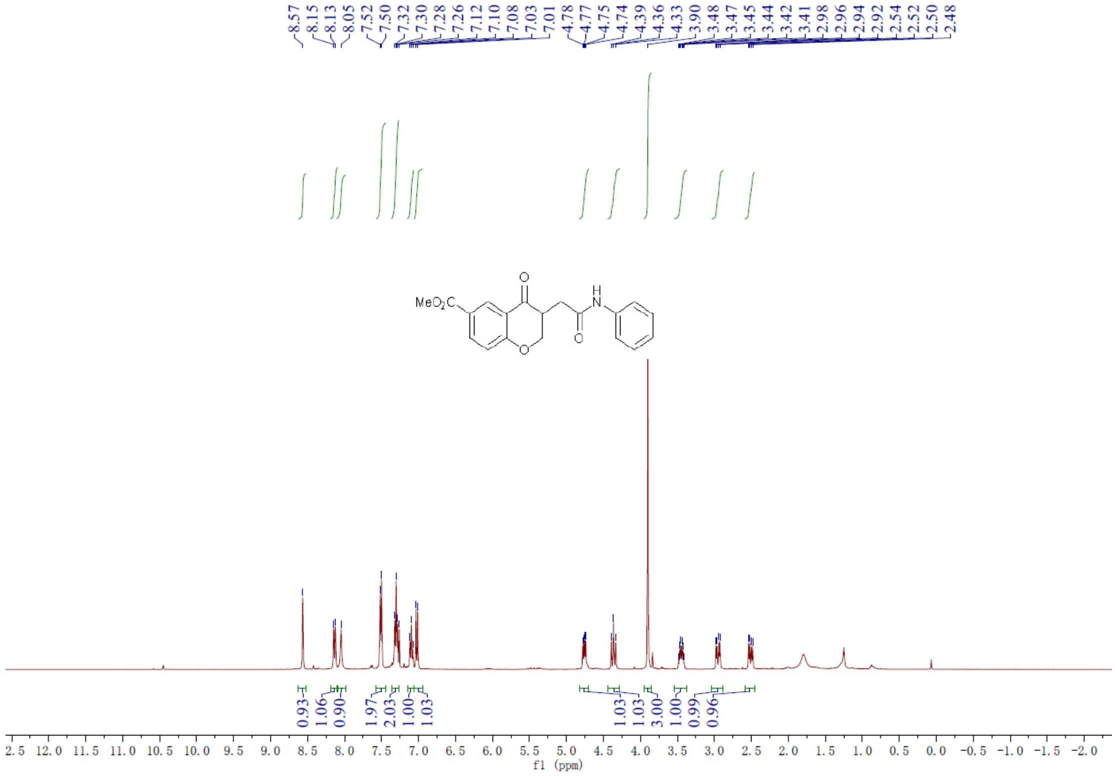

^{1}H spectra of **3na**

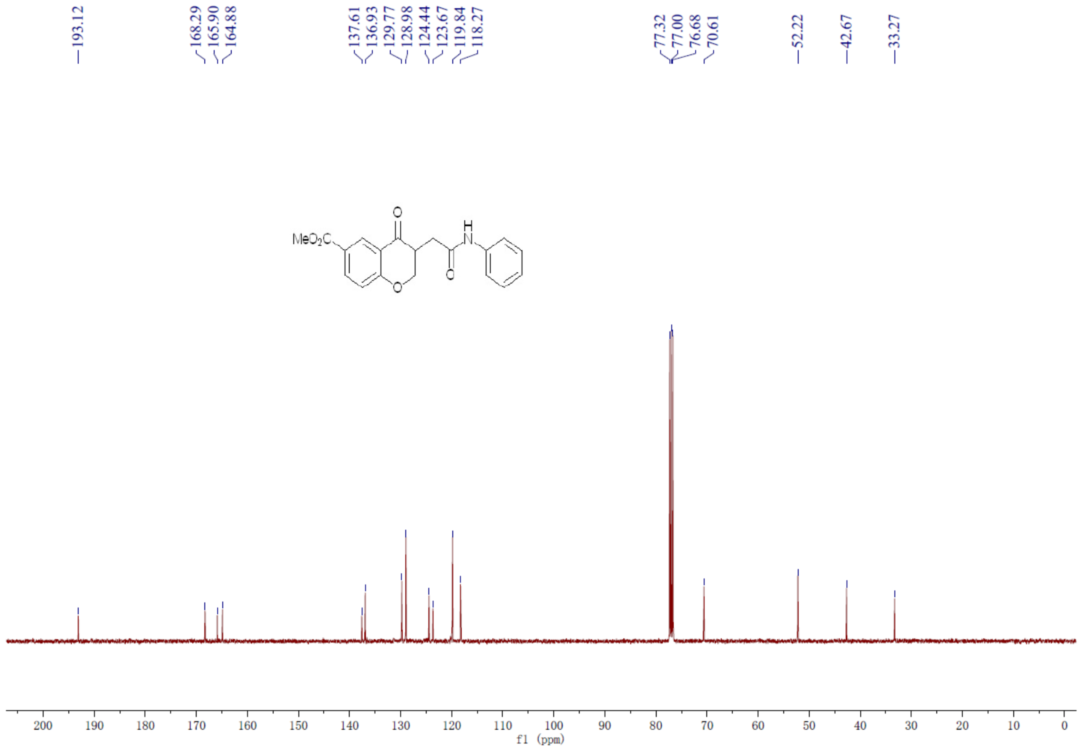

^{13}C spectra of **3na**

2-(1-oxo-2,3-dihydro-1H-benzo[f]chromen-2-yl)-*N*-phenylacetamide (3oa)

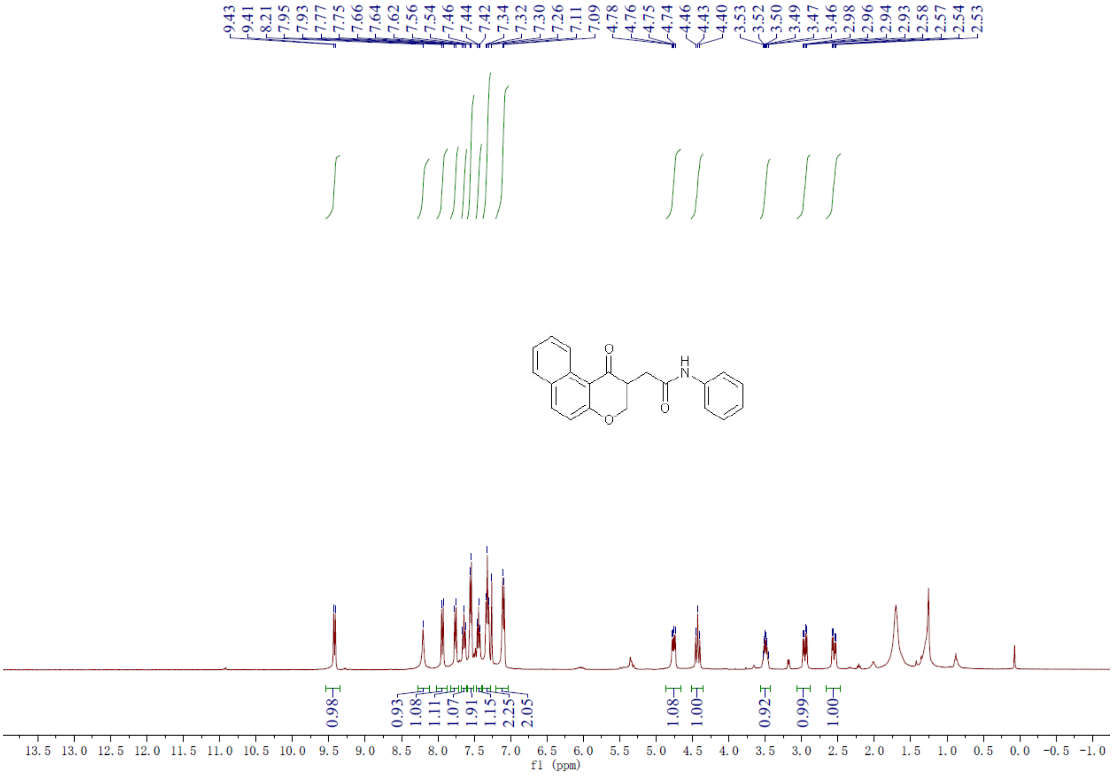

^{1}H spectra of **3oa**

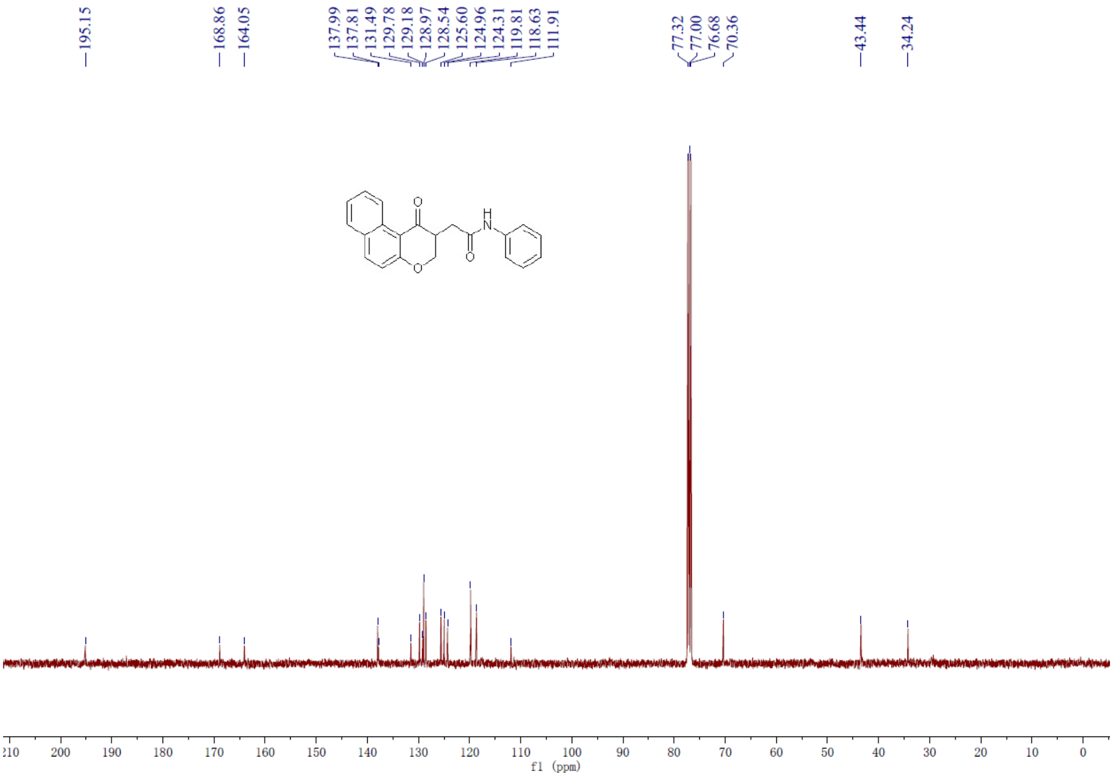

^{13}C spectra of **3oa**

2-(3-methyl-4-oxochroman-3-yl)-*N*-phenylacetamide (3pa)

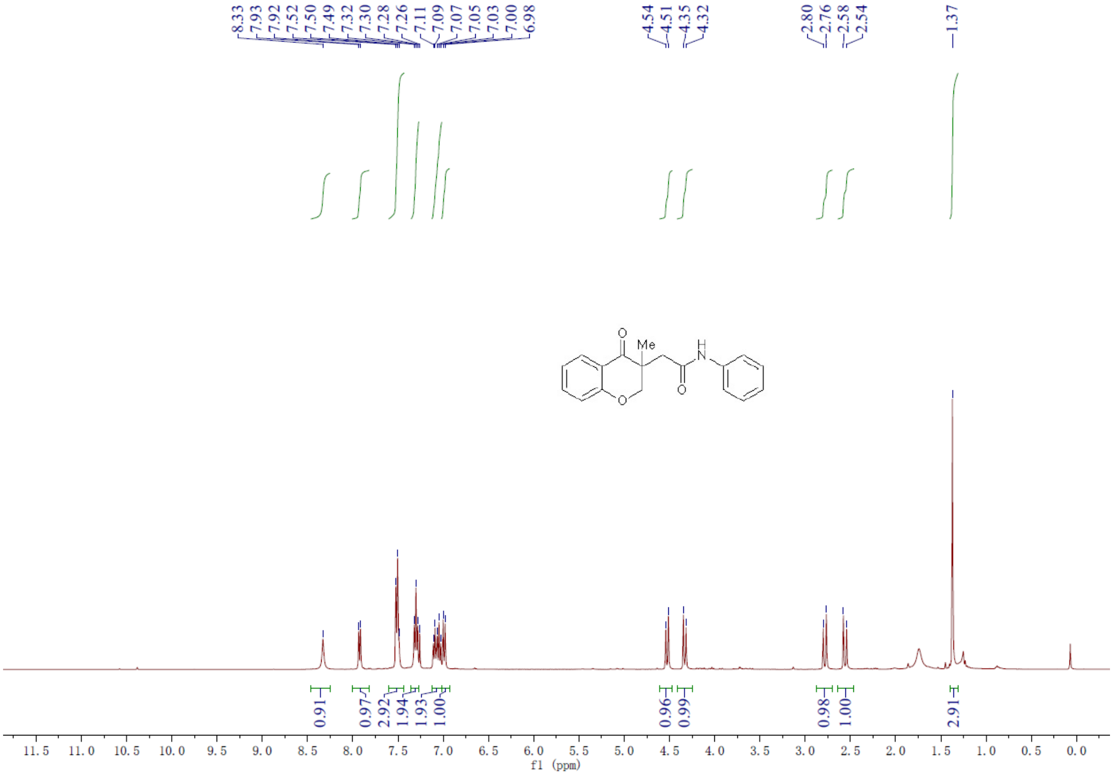

^{1}H spectra of **3pa**

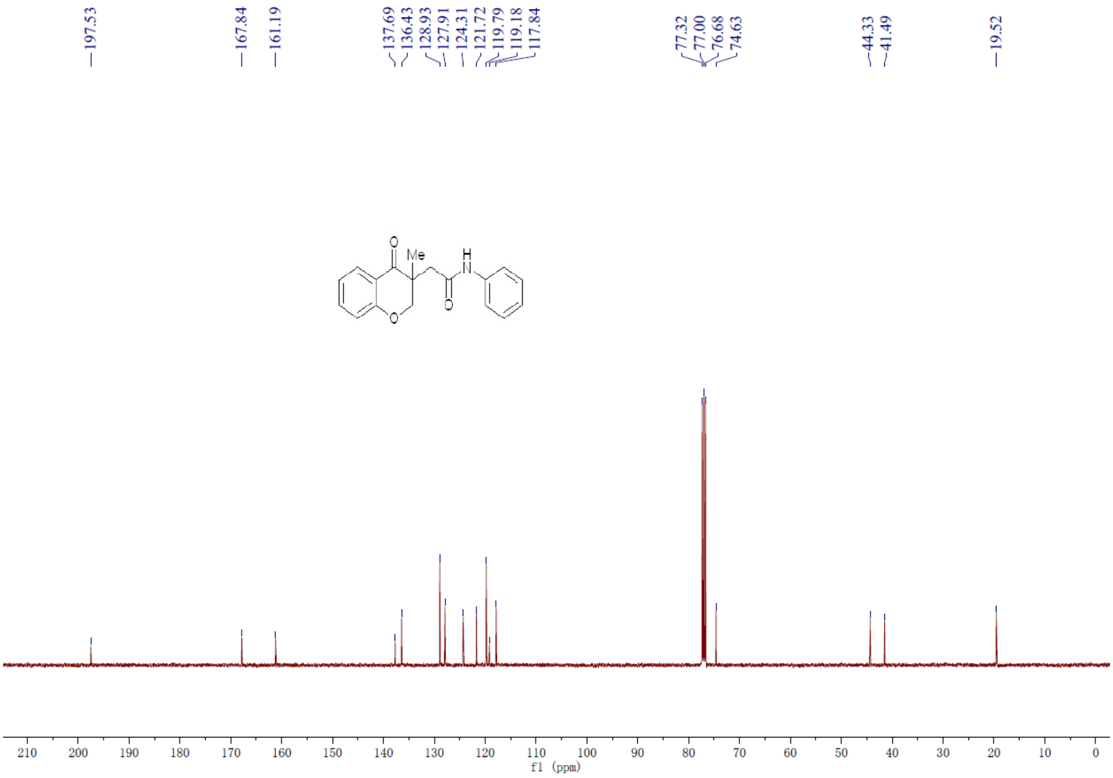

^{13}C spectra of **3pa**

2-(1-oxo-2,3-dihydro-1H-inden-2-yl)-*N*-phenylacetamide (3qa)

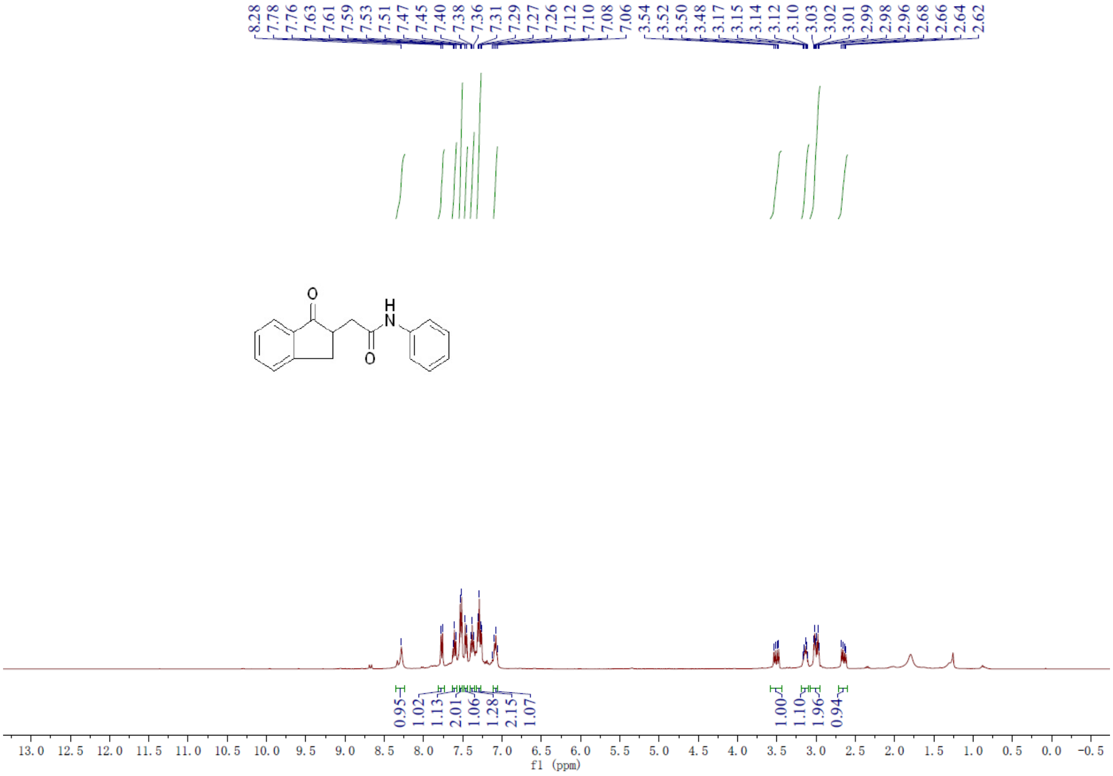

^{1}H spectra of **3qa**

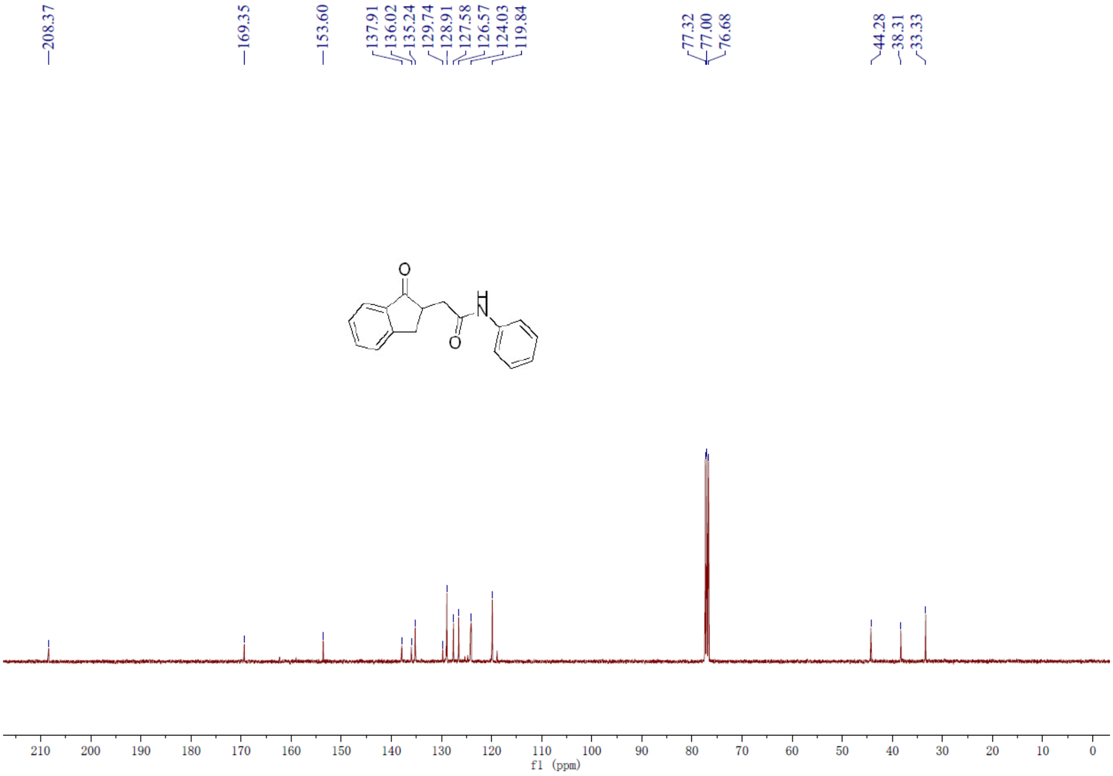

^{13}C spectra of **3qa**

2-(4-oxochroman-3-yl)-*N*-(p-tolyl)acetamide (3ab)

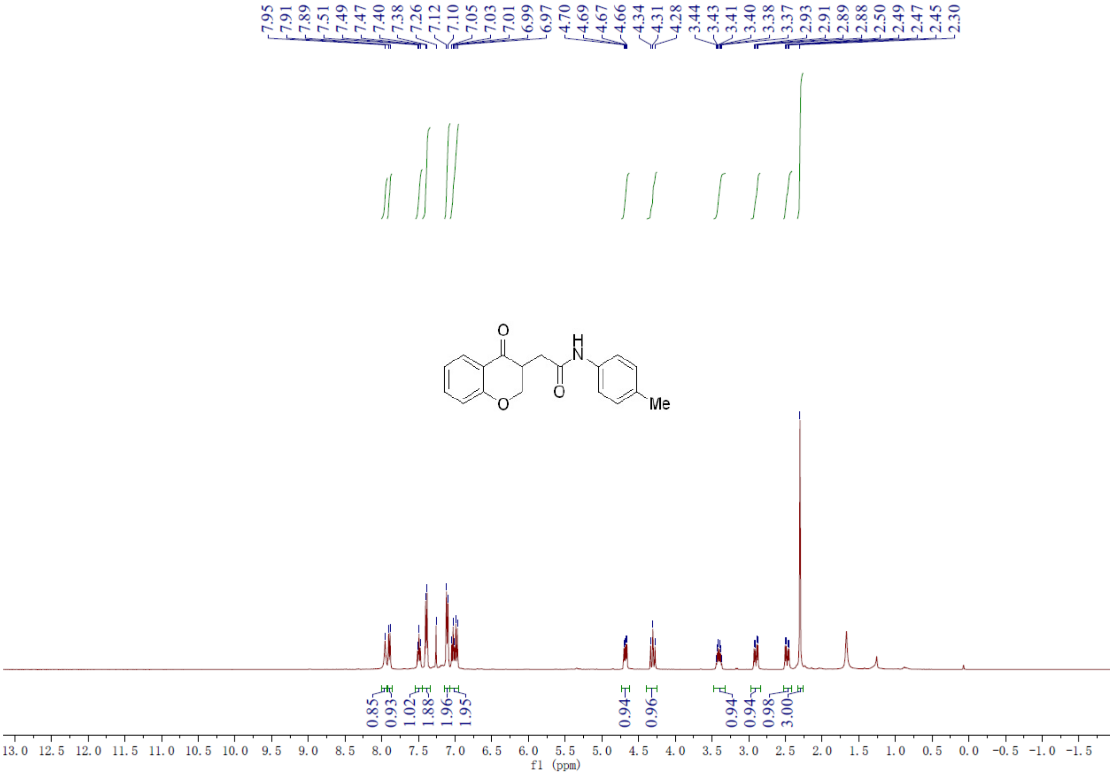

^{1}H spectra of **3ab**

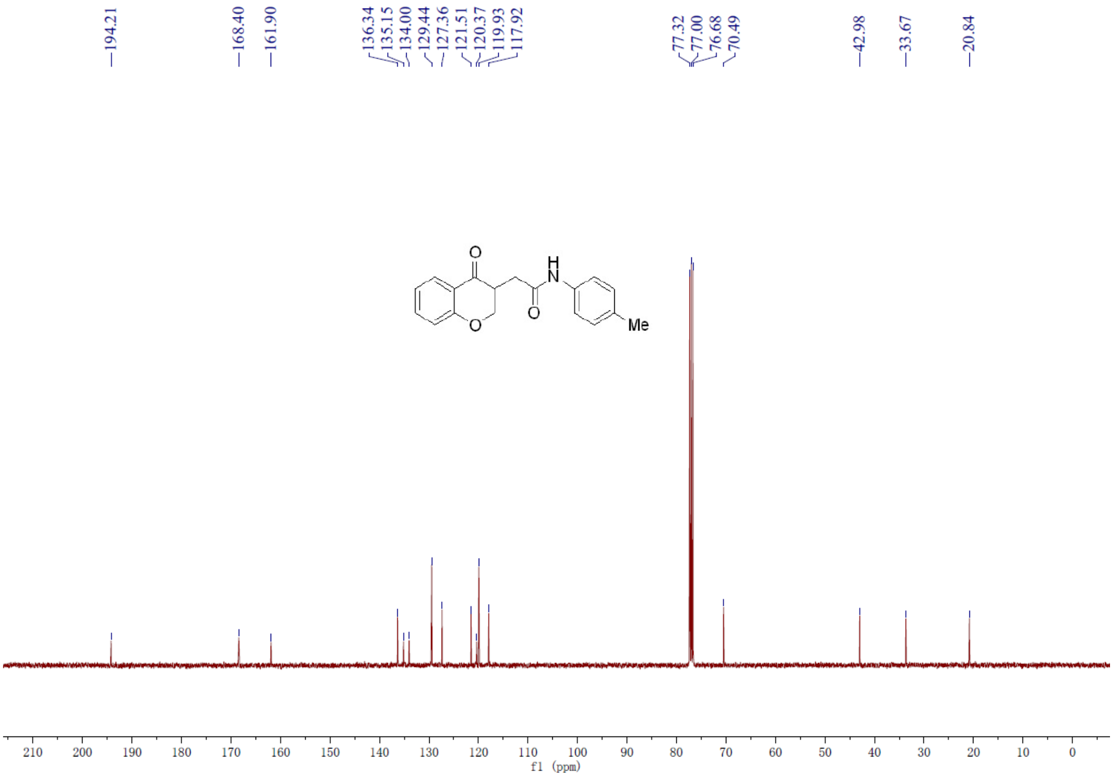

^{13}C spectra of **3ab**

_N_-(4-methoxyphenyl)-2-(4-oxochroman-3-yl)acetamide (3ac)

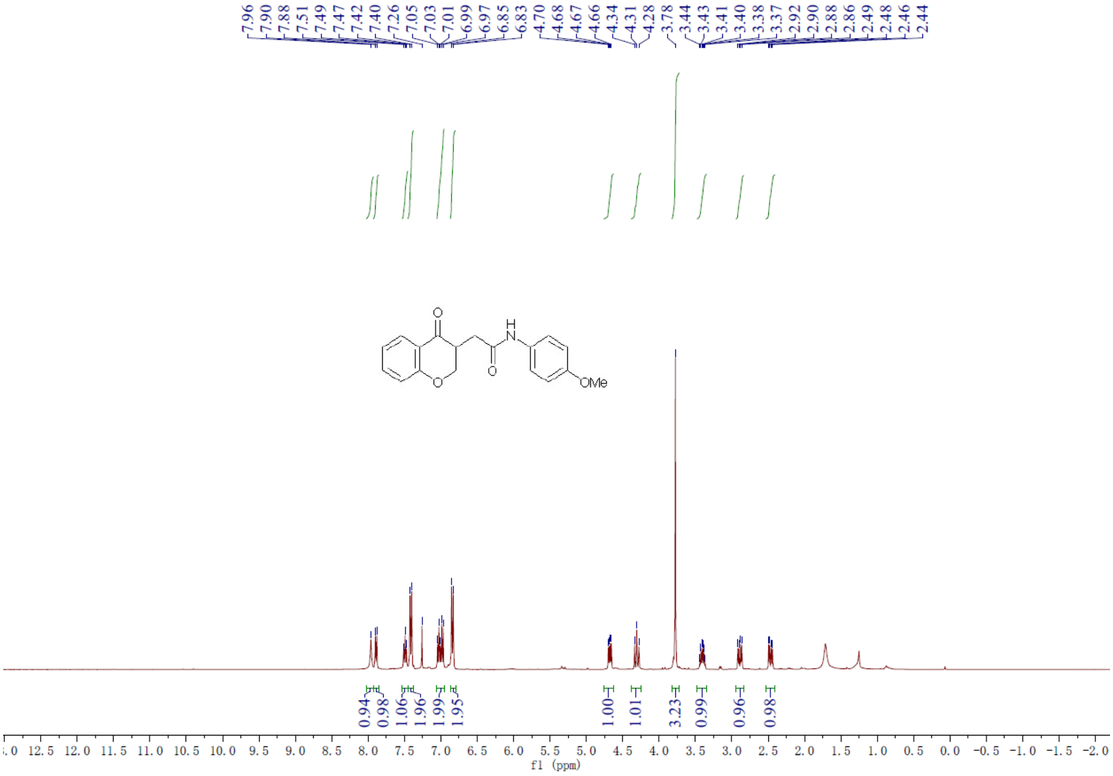

^{1}H spectra of **3ac**

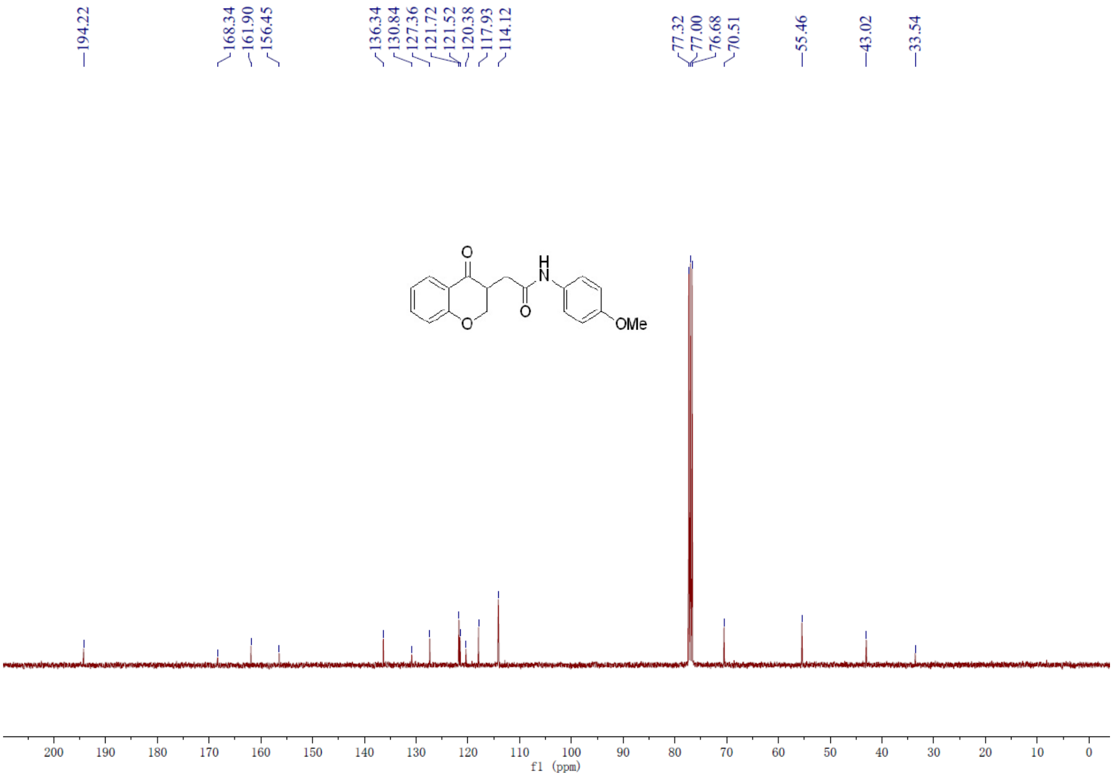

^{13}C spectra of **3ac**

***N*-(4-fluorophenyl)-2-(4-oxochroman-3-yl)acetamide (3ad)**

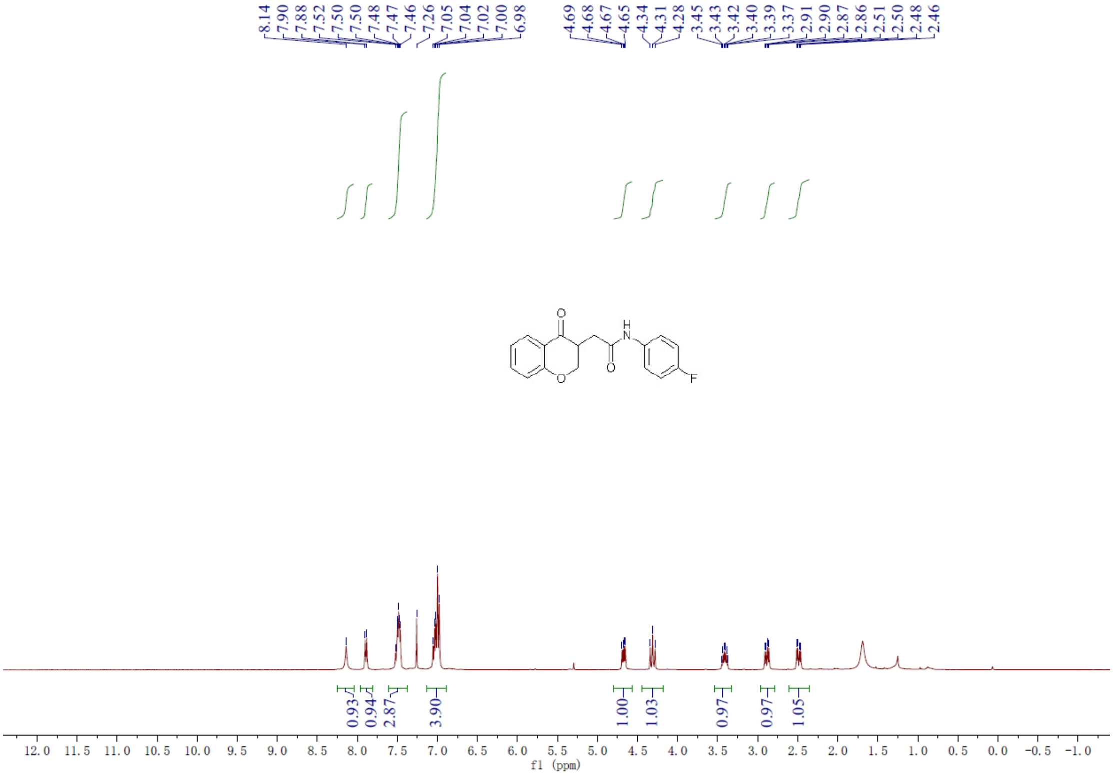

^{1}H spectra of **3ad**

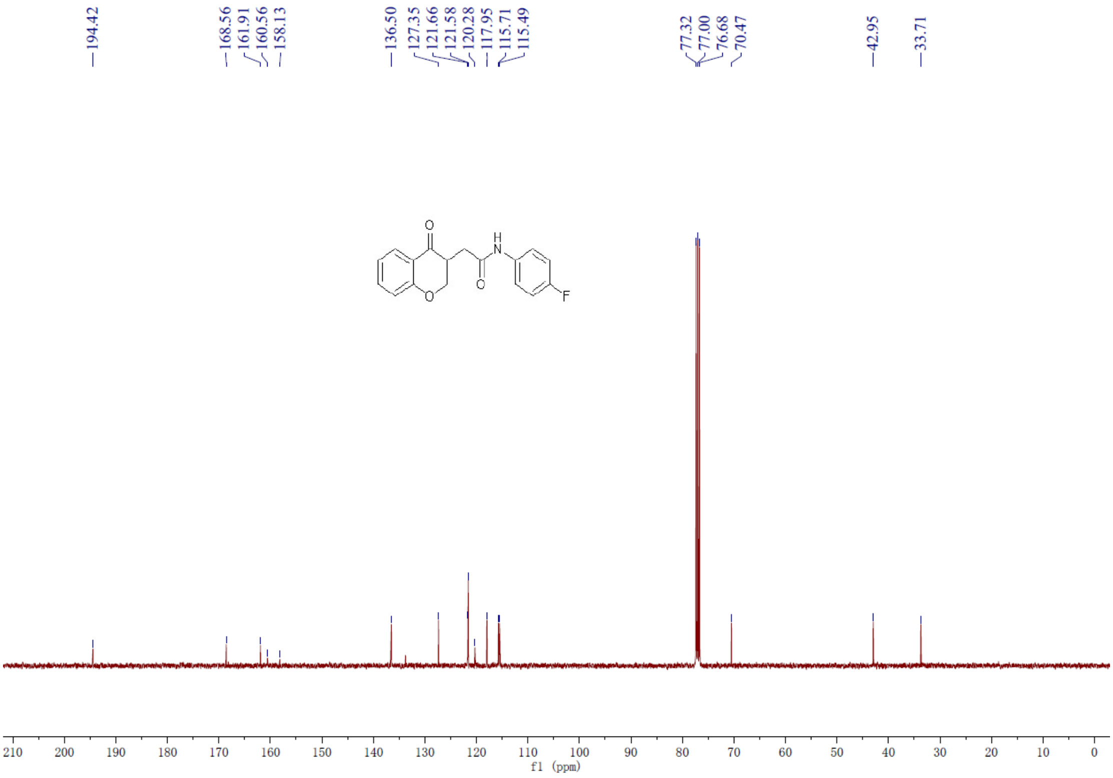

^{13}C spectra of **3ad**

N-(4-chlorophenyl)-2-(4-oxochroman-3-yl)acetamide (3ae)

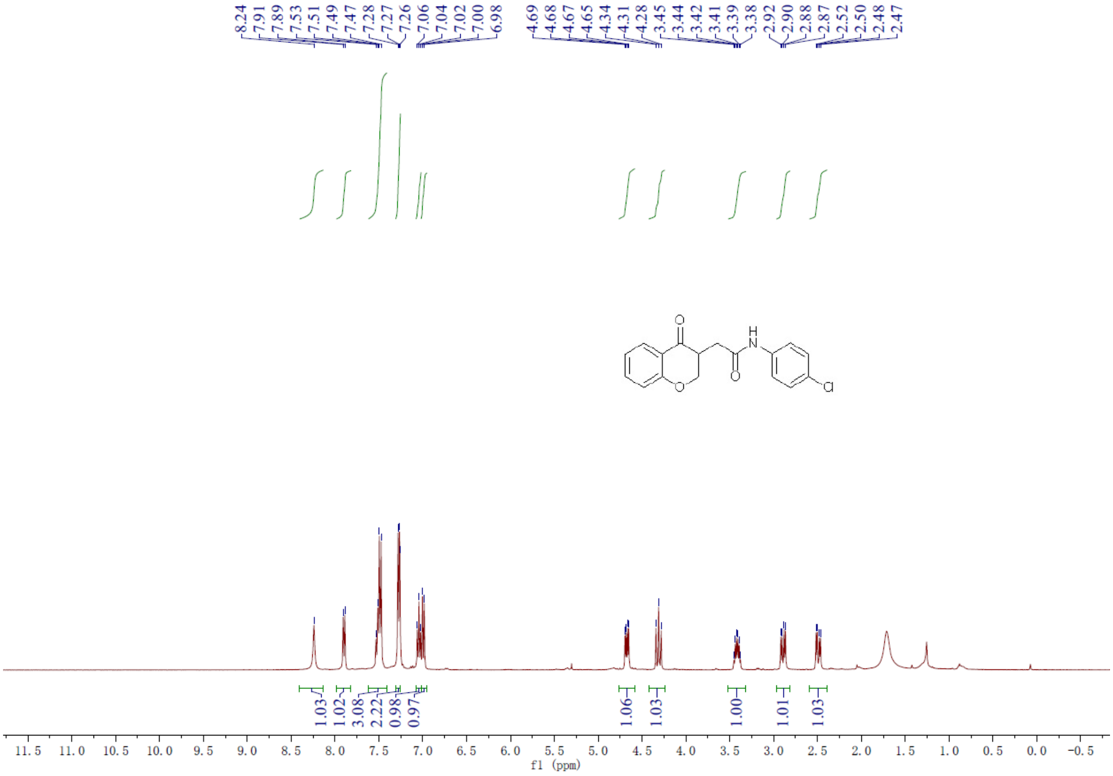

^{1}H spectra of **3ae**

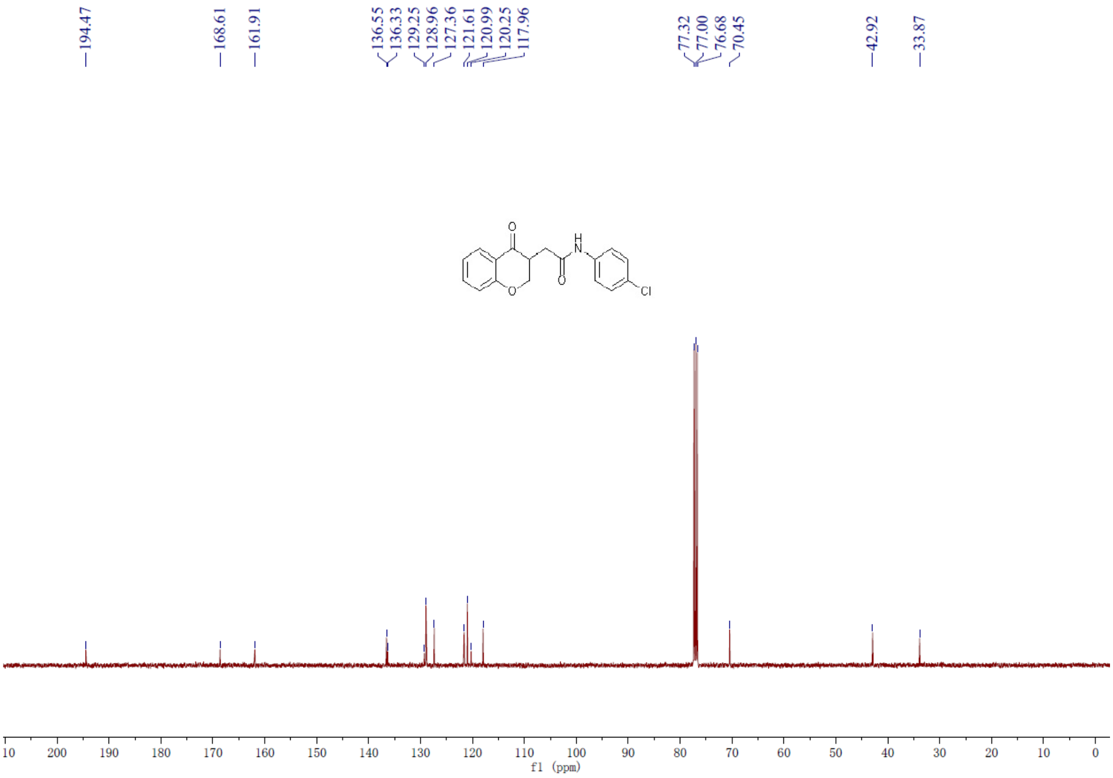

^{13}C spectra of **3ae**

***N*-(4-bromophenyl)-2-(4-oxochroman-3-yl)acetamide (3af)**

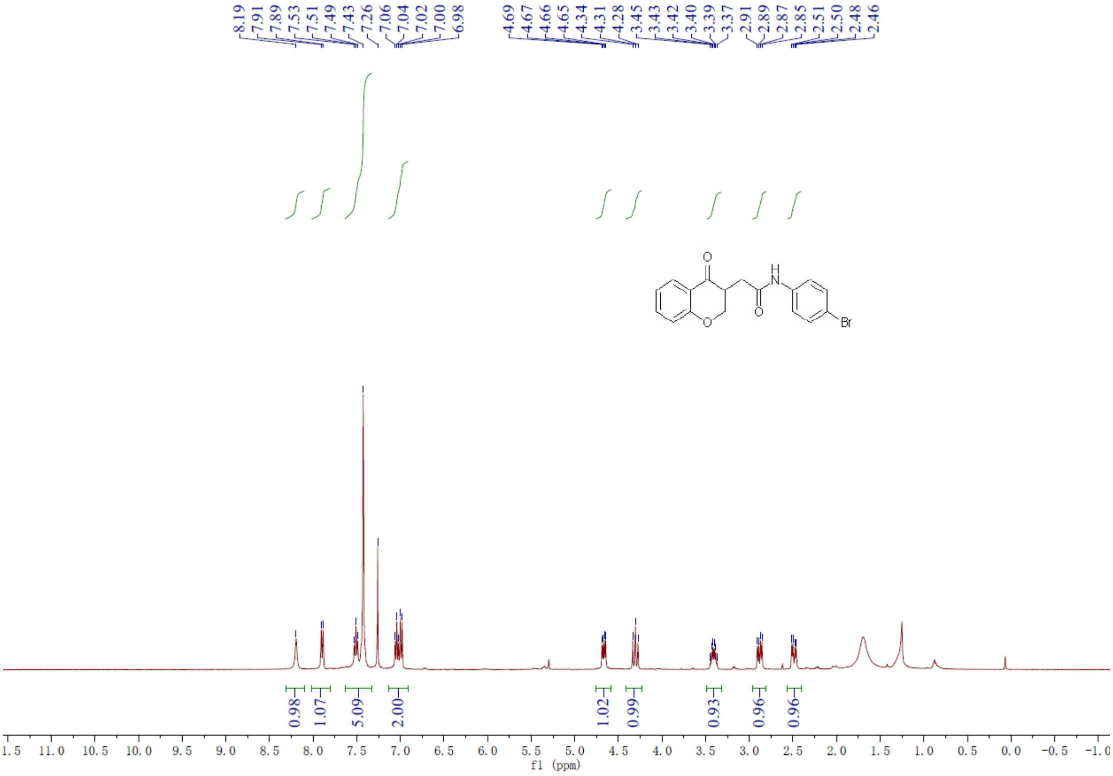

^{1}H spectra of **3af**

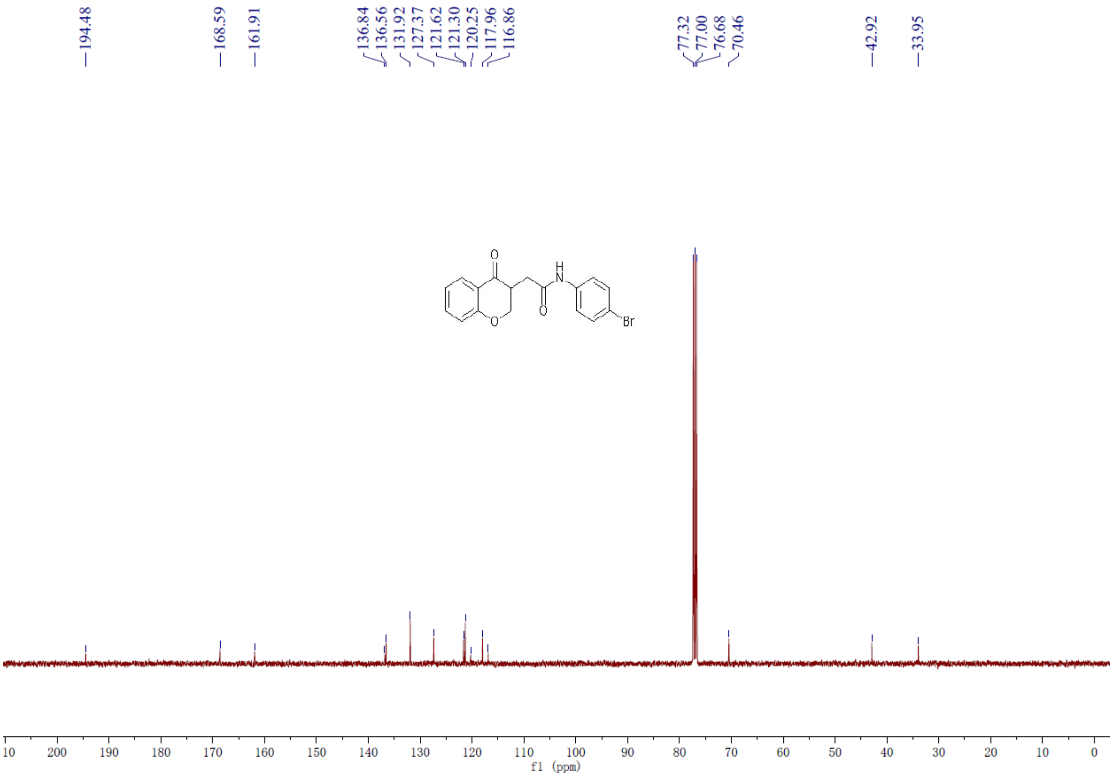

^{13}C spectra of **3af**

2-(4-oxochroman-3-yl)-*N*-(4-(trifluoromethyl)phenyl)acetamide (3ag)

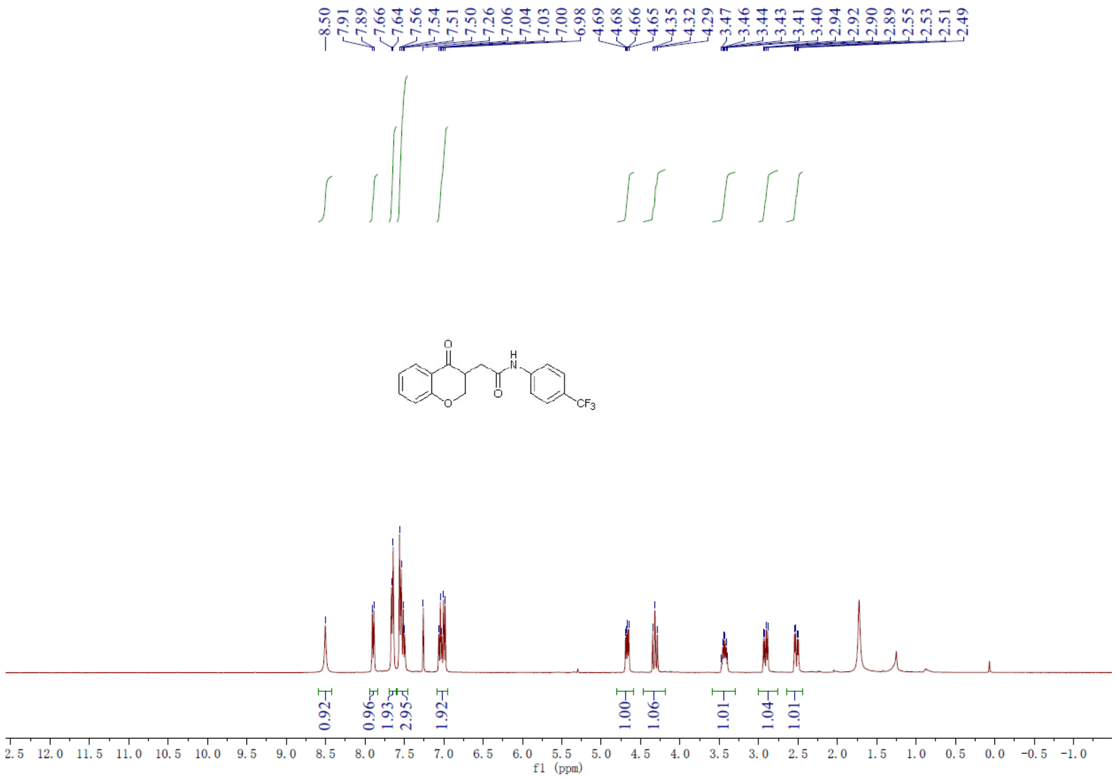

^{1}H spectra of **3ag**

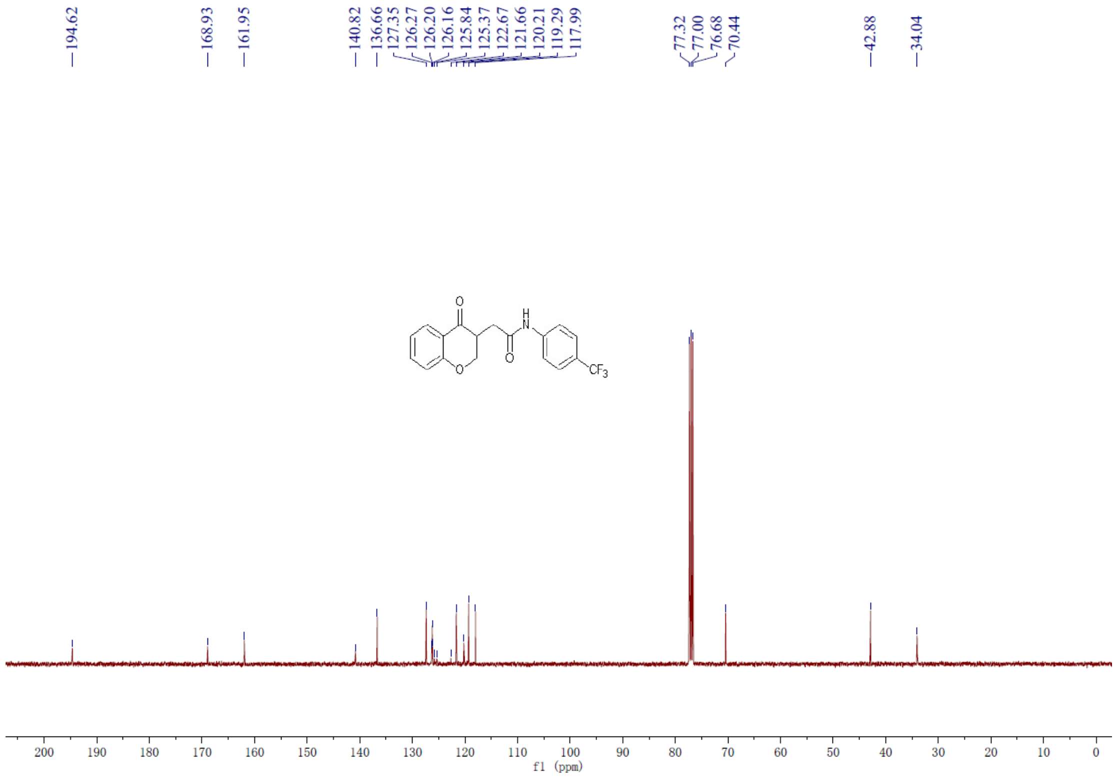

^{13}C spectra of **3ag**

_N_-(3-bromophenyl)-2-(4-oxochroman-3-yl)acetamide (3ah)

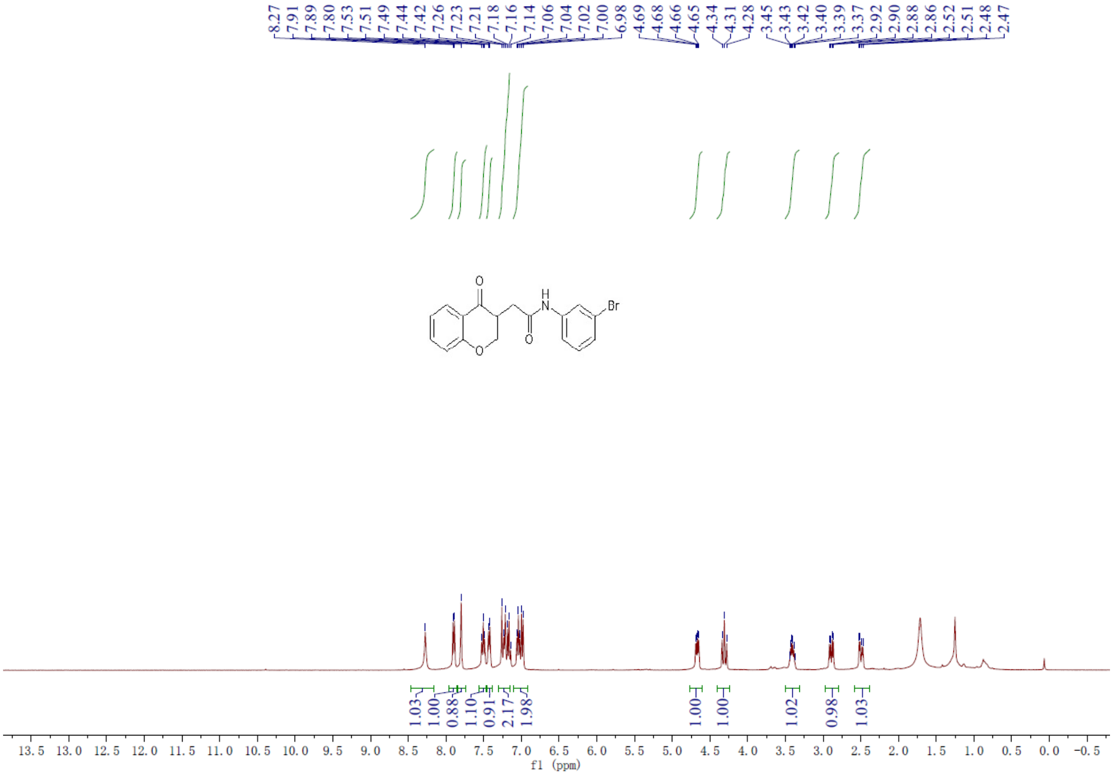

^{1}H spectra of **3ah**

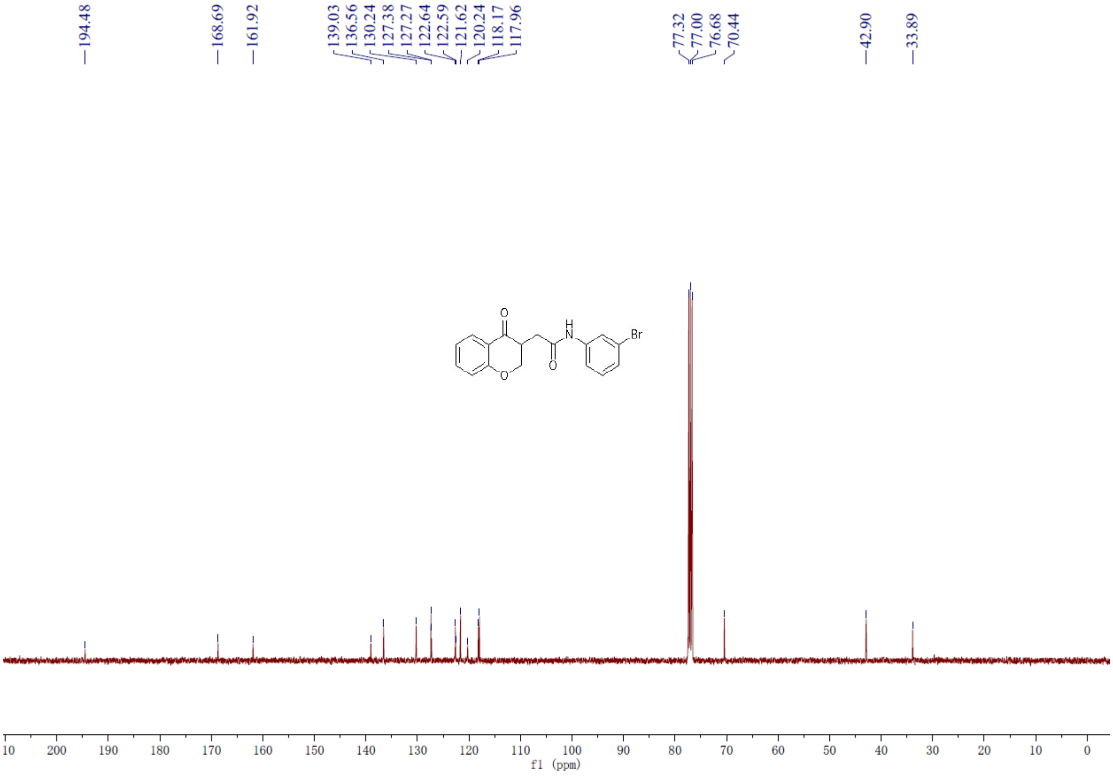

^{13}C spectra of **3ah**

***N*-benzyl-2-(4-oxochroman-3-yl)acetamide (3ai)**

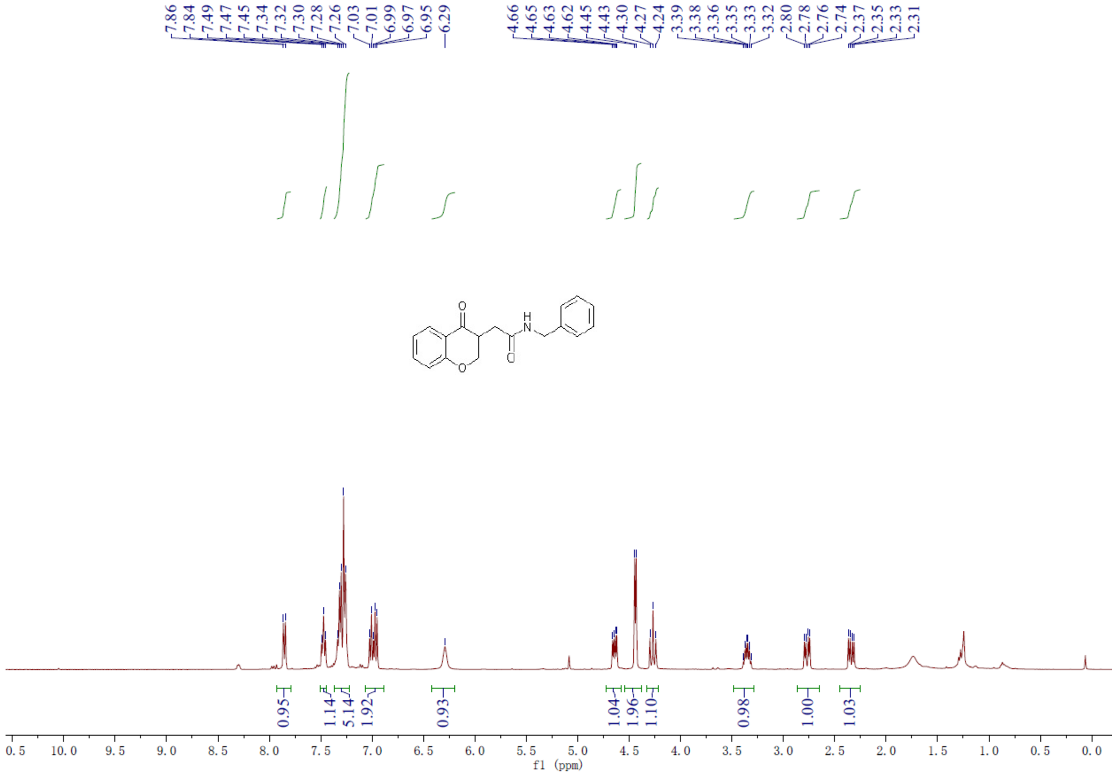

^{1}H spectra of **3ai**

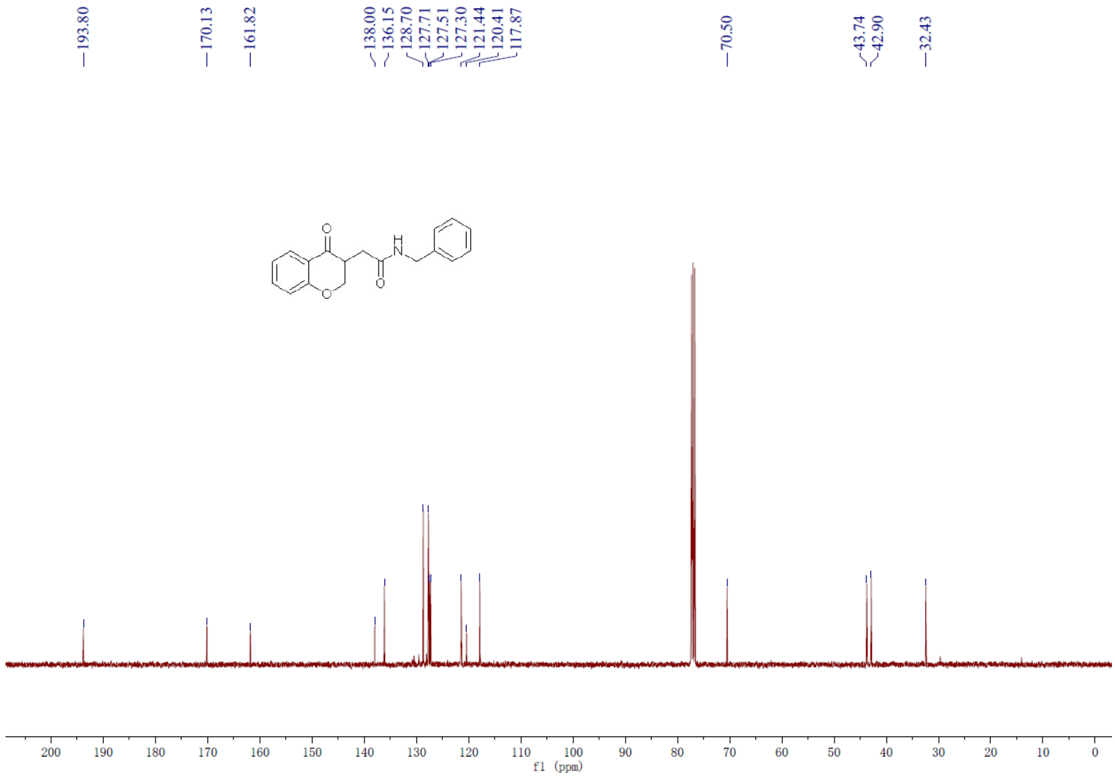

^{13}C spectra of **3ai**

***N*-cyclohexyl-2-(4-oxochroman-3-yl)acetamide (3aj)**

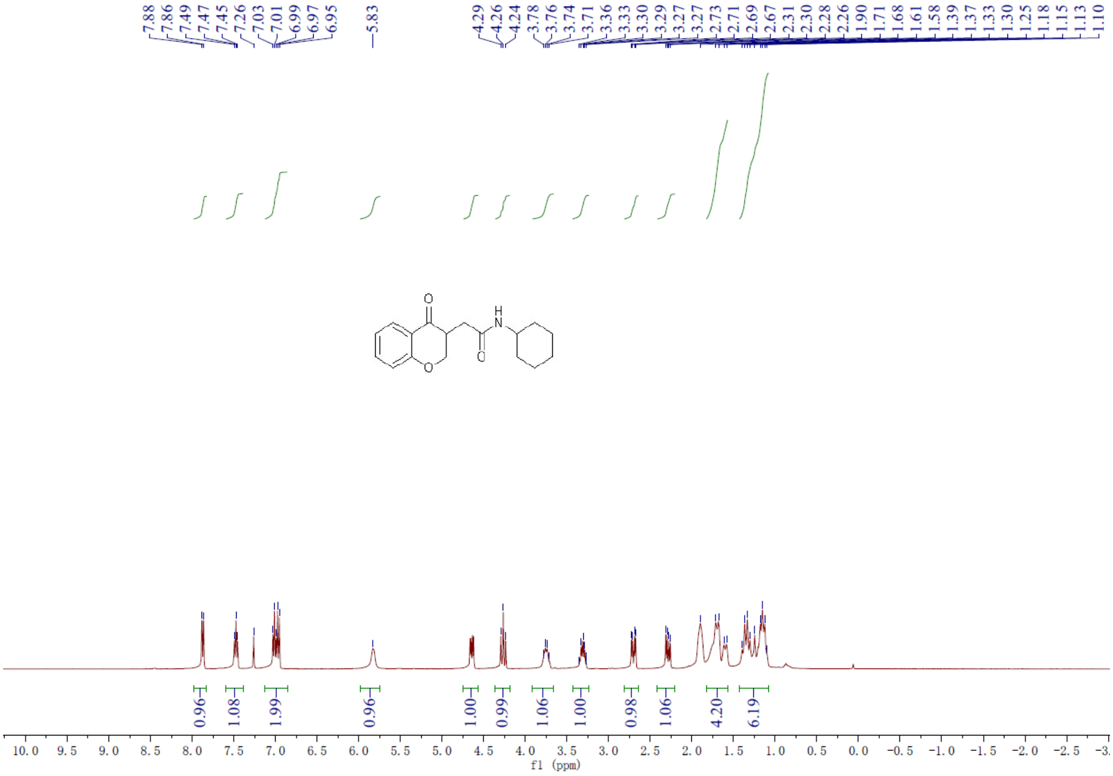

^{1}H spectra of **3aj**

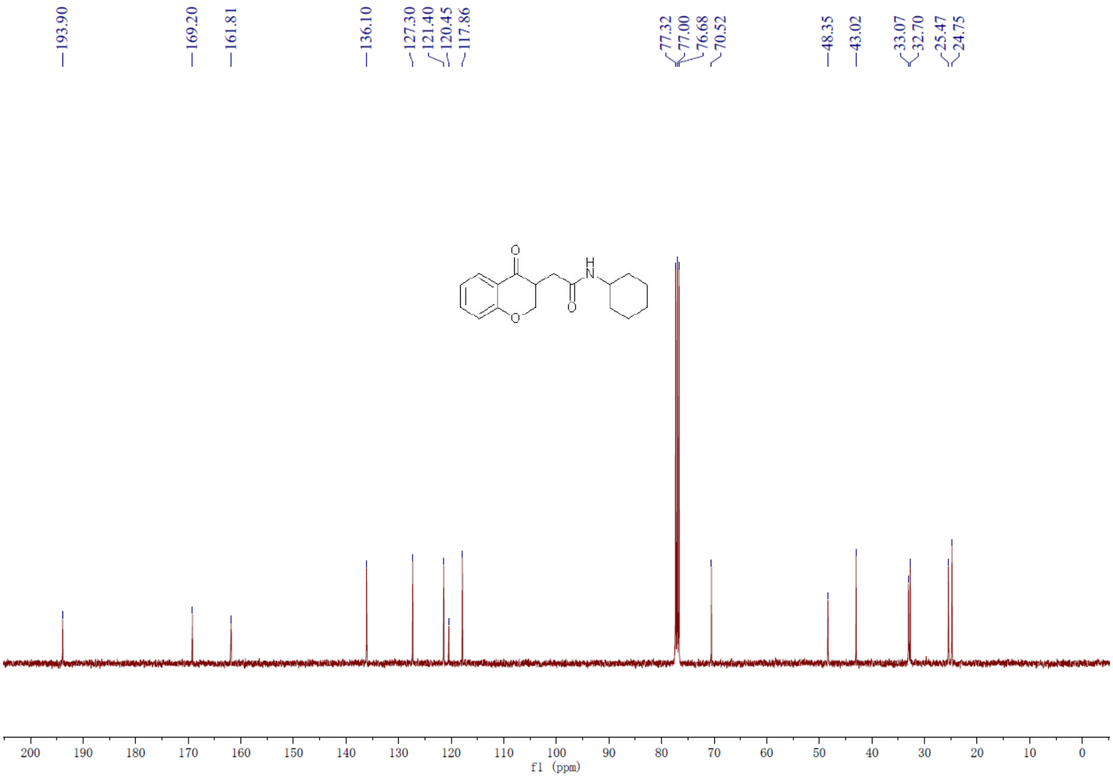

^{13}C spectra of **3aj**

N-cyclopentyl-2-(4-oxochroman-3-yl)acetamide (3ak)

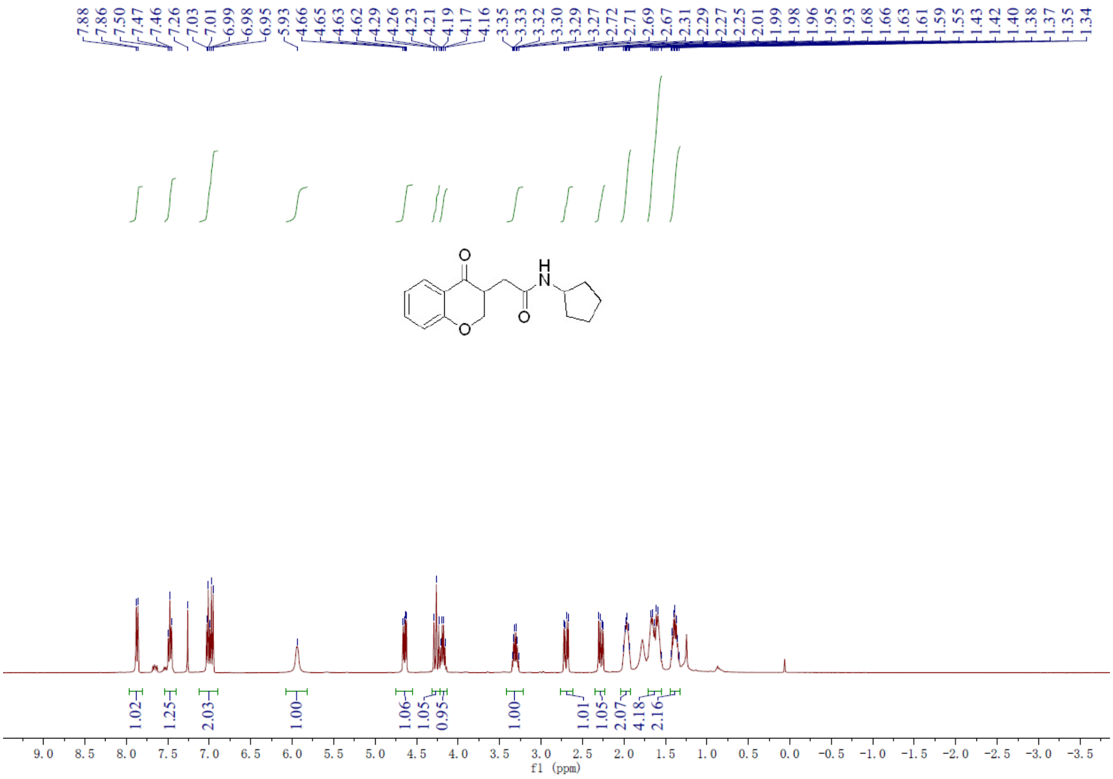

^{1}H spectra of **3ak**

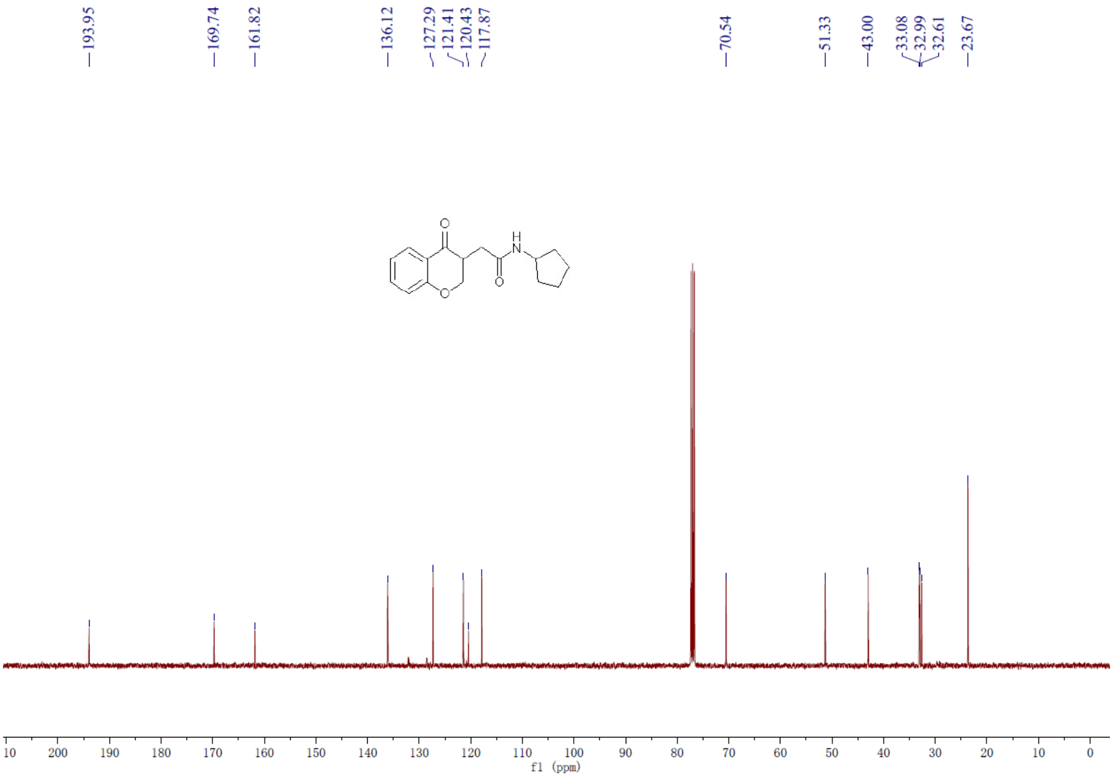

^{13}C spectra of **3ak**

N-butyl-2-(4-oxochroman-3-yl)acetamide (3al)

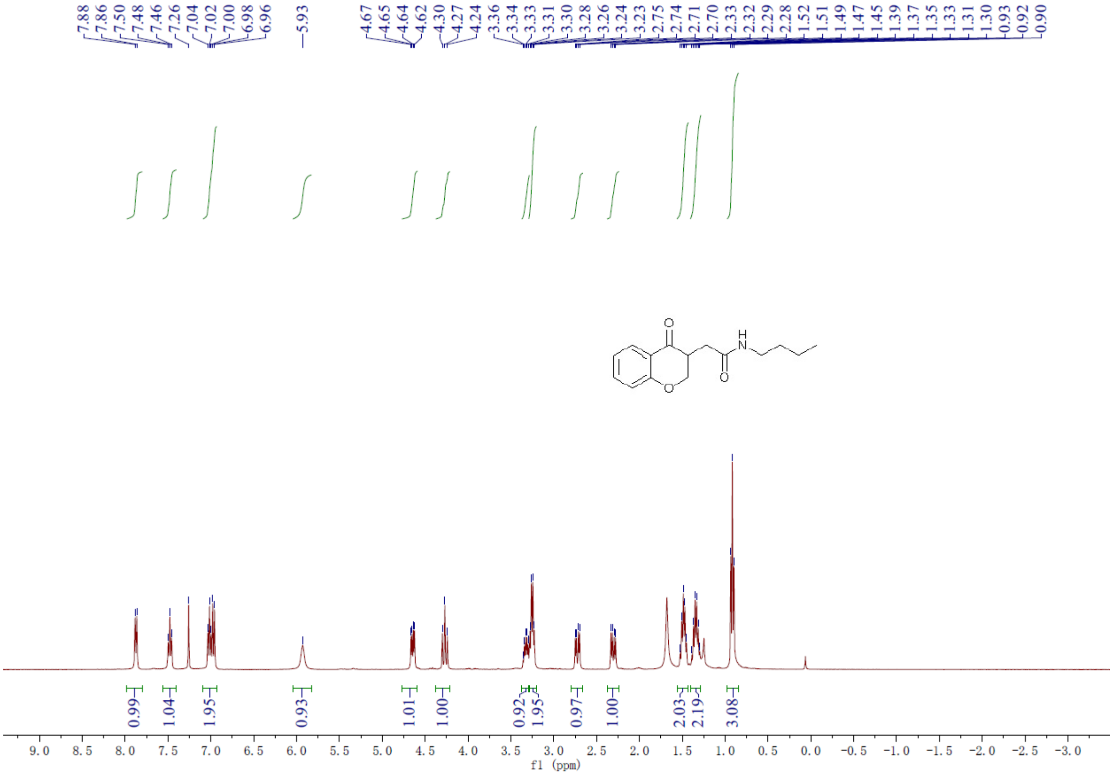

^{1}H spectra of **3al**

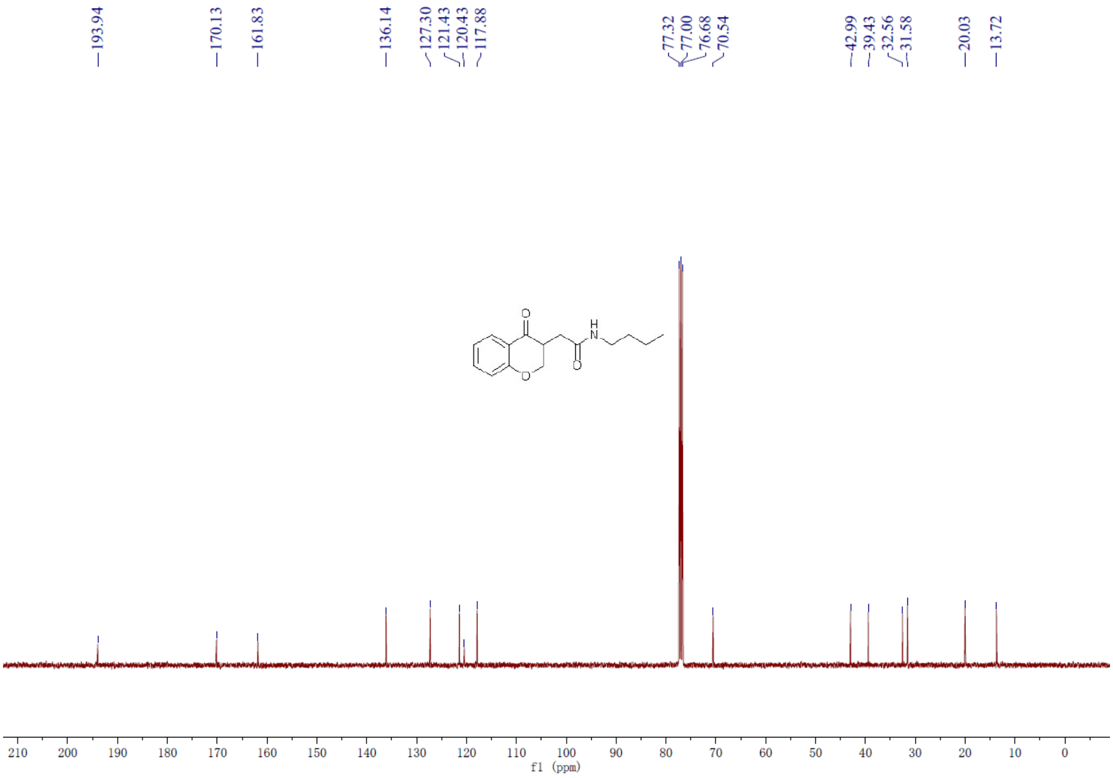

^{13}C spectra of **3al**

3-(2-morpholino-2-oxoethyl)chroman-4-one (3am)

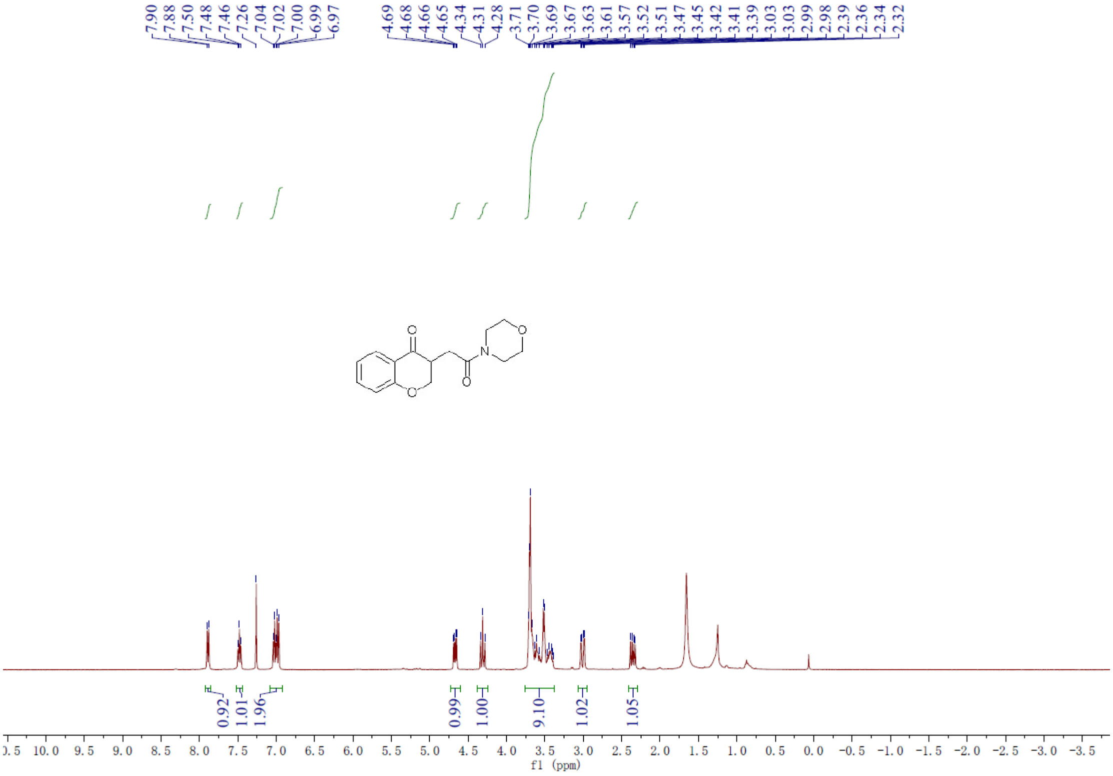

^{1}H spectra of **3am**

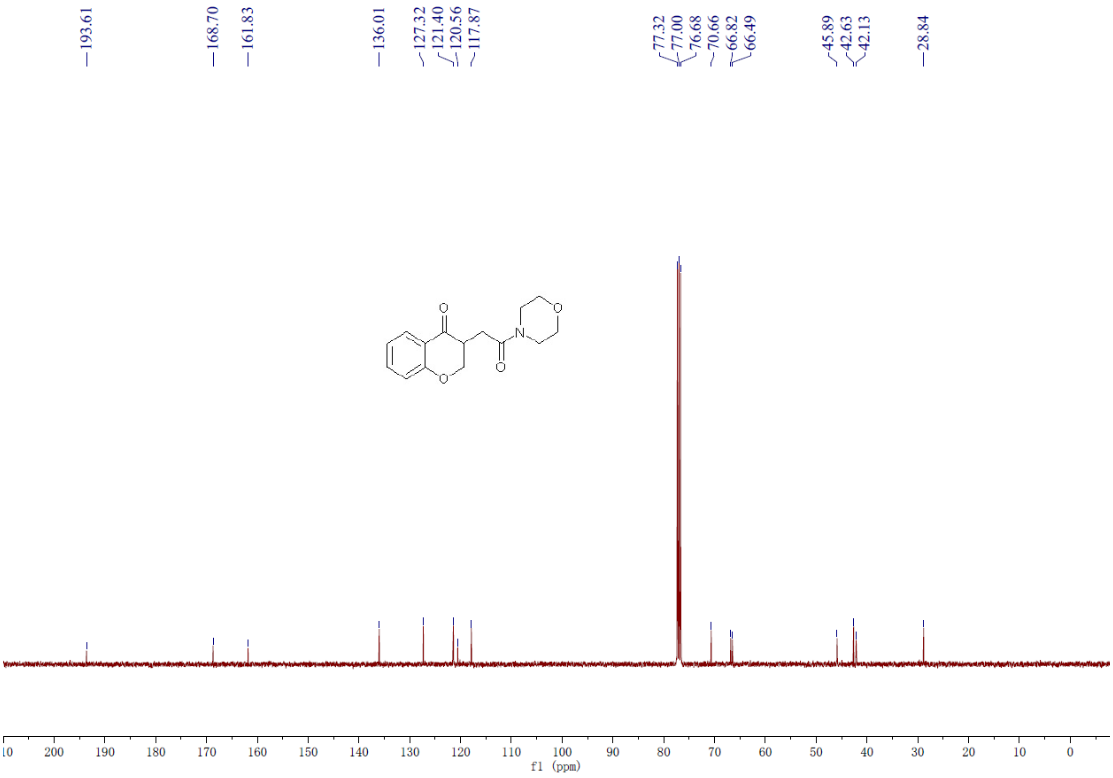

^{13}C spectra of **3am**

***N*-(adamantan-1-yl)-2-(4-oxochroman-3-yl)acetamide (3an)**

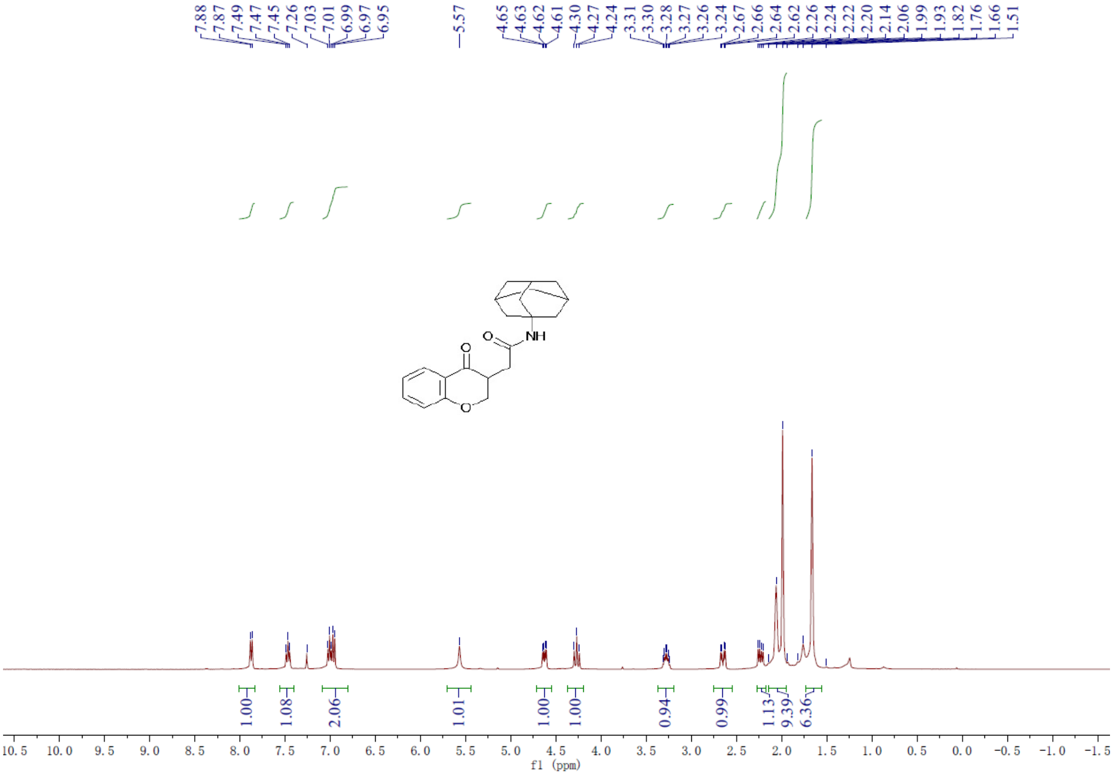

^{1}H spectra of **3an**

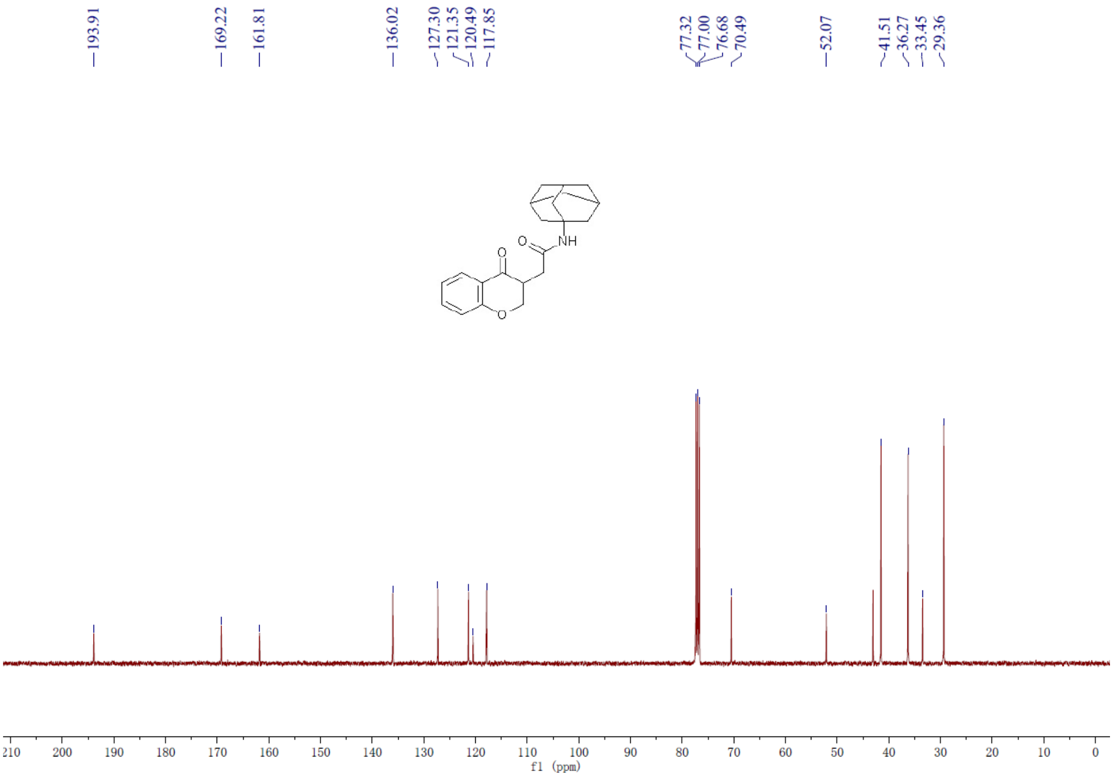

^{13}C spectra of **3an**
